# Novel multi-target ligands of dopamine and serotonin receptors for the treatment of schizophrenia based on indazole and piperazine scaffolds–synthesis, biological activity, and structural evaluation

**DOI:** 10.1080/14756366.2023.2209828

**Published:** 2023-05-15

**Authors:** Piotr Stępnicki, Olga Wronikowska-Denysiuk, Agata Zięba, Katarzyna M. Targowska-Duda, Agata Bartyzel, Martyna Z. Wróbel, Tomasz M. Wróbel, Klaudia Szałaj, Andrzej Chodkowski, Karolina Mirecka, Barbara Budzyńska, Emilia Fornal, Jadwiga Turło, Marián Castro, Agnieszka A. Kaczor

**Affiliations:** a Department of Synthesis and Chemical Technology of Pharmaceutical Substances, Faculty of Pharmacy, Medical University of Lublin, Lublin, Poland; b Independent Laboratory of Behavioral Studies, Chair of Biomedical Sciences, Faculty of Biomedicine, Medical University of Lublin, Lublin, Poland; c Department of Biopharmacy, Faculty of Pharmacy, Medical University of Lublin, Lublin, Poland; d Department of General and Coordination Chemistry and Crystallography, Institute of Chemical Sciences, Faculty of Chemistry, Maria Curie-Sklodowska University in Lublin, Lublin, Poland; e Department of Drug Technology and Pharmaceutical Biotechnology, Faculty of Pharmacy, Medical University of Warsaw, Warsaw, Poland; f Department of Bioanalytics, Faculty of Biomedicine, Medical University of Lublin, Lublin, Poland; g Department of Pharmacology, Center for Research in Molecular Medicine and Chronic Diseases (CIMUS), Universidade de Santiago de Compostela, Santiago de Compostela, Spain; h Instituto de Investigación Sanitaria de Santiago de Compostela (IDIS), Santiago de Compostela, Spain; i School of Pharmacy, University of Eastern Finland, Kuopio, Finland

**Keywords:** Antipsychotic, schizophrenia, multi-target ligands, GPCR

## Abstract

Schizophrenia is a chronic mental disorder that is not satisfactorily treated with available antipsychotics. The presented study focuses on the search for new antipsychotics by optimising the compound D2AAK3, a multi-target ligand of G-protein-coupled receptors (GPCRs), in particular D_2_, 5-HT_1A_, and 5-HT_2A_ receptors. Such receptor profile may be beneficial for the treatment of schizophrenia. Compounds **1**–**16** were designed, synthesised, and subjected to further evaluation. Their affinities for the above-mentioned receptors were assessed in radioligand binding assays and efficacy towards them in functional assays. Compounds **1** and **10**, selected based on their receptor profile, were subjected to *in vivo* tests to evaluate their antipsychotic activity, and effect on memory and anxiety processes. Molecular modelling was performed to investigate the interactions of the studied compounds with D_2_, 5-HT_1A_, and 5-HT_2A_ receptors on the molecular level. Finally, X-ray study was conducted for compound **1**, which revealed its stable conformation in the solid state.

## Graphical Abstract

**Figure:**
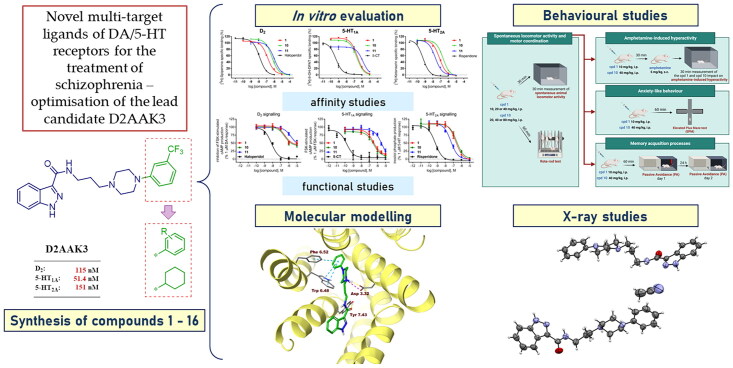

## Introduction

Schizophrenia is defined as a severe, chronic mental disorder characterised by disturbances in behaviour and thought processes, and impaired perception, emotional responsiveness, and cognitive functioning. The most common symptoms of schizophrenia include hallucinations, delusions, hearing voices, social withdrawal, anhedonia, and disorganised speech^1^. The onset of the disease is usually noted in late adolescence; however, the first symptoms may be observed with a delay of even several years, preceded by a period of subtle changes in behaviour^2–4^.

Certain environmental and genetic factors may predispose to development of schizophrenia; however, its exact causes remain unclear. There are several hypotheses aimed at explaining the underlying causes of the disease. The dominant one is based on the disturbances of dopaminergic neurotransmission in the mesolimbic circuit in the central nervous system, which leads to positive symptoms, such as hallucinations and delusions, and in the mesocortical circuit, which is suggested to produce negative symptoms, such as asociality or anhedonia^5^. Attempts are being made to develop antipsychotic drugs acting through a non-dopaminergic mechanism. In particular, the trace amine-associated receptor 1 (TAAR1) has come under the spotlight in recent years as a potential target for new drugs against schizophrenia^6^. TAAR1 belongs to the family of G-protein-coupled receptors (GPCRs) and is involved in modulation of dopaminergic, serotonergic, and glutamatergic neurotransmissions^7^. Two TAAR1 agonists, ulotaront and ralmitaront, currently undergo clinical trials^6^^,^^8^. In particular, the first one shows effectiveness in relation to positive, negative, and cognitive symptoms, and does not cause side effects characteristic of marketed antipsychotics, resulting mainly from the interaction with dopamine D_2_ receptor^8^.

Based on the mechanism of action, drugs approved for the treatment of schizophrenia are divided into three categories. The first-generation, so-called classical drugs are antagonists of dopamine D_2_ receptor, whereas second-generation drugs, often referred to as atypical antipsychotics, in majority block both serotonin 5-HT_2A_ and dopamine D_2_ receptors, with greater affinity for the former. Drugs that fall into third-generation, sometimes termed “dopamine stabilizers”, are partial agonists of D_2_ and 5-HT_1A_ receptors. Classical drugs show good efficacy against positive symptoms of schizophrenia but usually fail in treating negative symptoms and cognitive deficits. The additional interaction with serotonin receptors of newer drugs may be beneficial in the context of relieving symptoms that are resistant to treatment with classical drugs^9^, as well as may contribute to improved safety profile of those drugs, in particular with regard to extrapyramidal symptoms, which are one of the most frequent and severe adverse reactions associated with the use of first-generation antipsychotics^10^. Thus, such drugs with a multi-receptor profile are generally considered superior to selective dopamine D_2_ receptor antagonists in terms of effectiveness against all groups of schizophrenia symptoms and their safety profile. Given the complex mechanisms underlying the pathogenesis of schizophrenia, which involve multiple neurotransmitter systems, such multi-target approach currently predominates in drug discovery campaigns aimed at identifying novel antipsychotics^11^.

**Figure 1.**
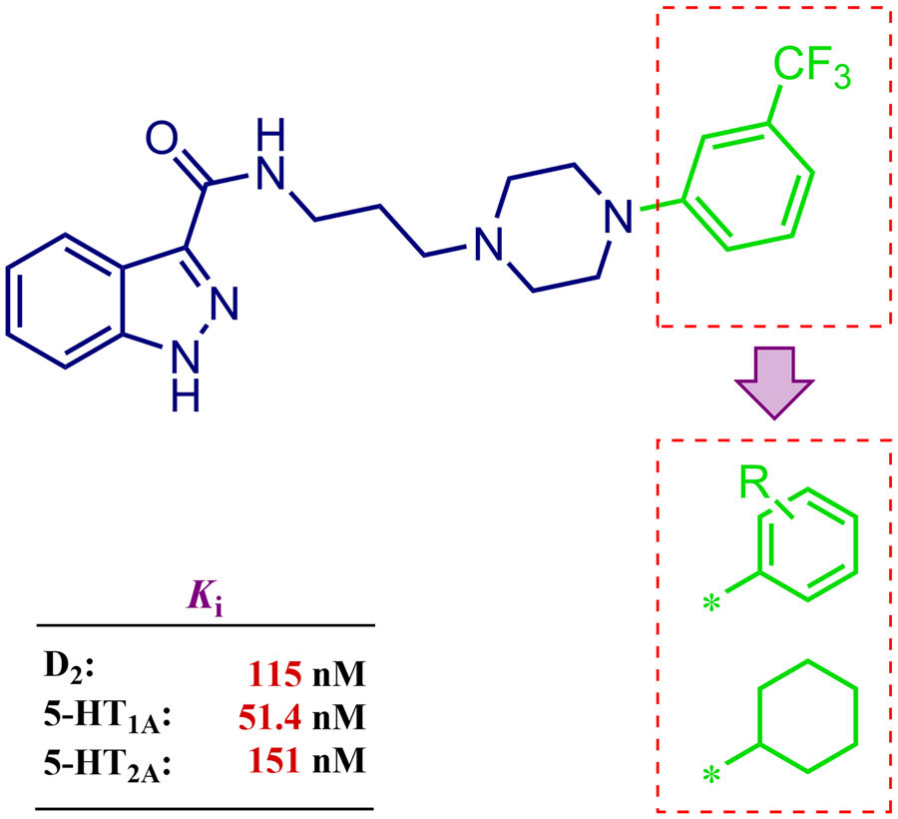
Chemical structure of D2AAK3 and its structural modifications designed in the optimisation process.

Considering the above, in this work, we attempted to obtain new multi-target compounds as potential drugs against schizophrenia. As part of the previously conducted research, compound D2AAK3 was identified and evaluated. It shows an affinity for the molecular targets important for the treatment of schizophrenia, in particular D_2_, 5-HT_1A_, and 5-HT_2A_ receptors (Figure 1), and also reduces amphetamine-induced hyperactivity in mice, an experimental model predictive of antipsychotic activity *in vivo*. Moreover, it improves memory processes as indicated in passive avoidance (PA) test, and shows anxiogenic activity 30 min after acute treatment but exerts no effect on anxiety processes after another 30 min in elevated plus maze (EPM) assay^12^. Given the favourable pharmacological properties, compound D2AAK3 may be considered a good candidate for the lead structure and starting point for the optimisation campaign aimed at fine-tuning its receptor profile, especially by increasing 5-HT_2A_/D_2_ receptors affinity ratio, in order to obtain molecules that more closely resemble atypical antipsychotics. For this purpose, modifications of D2AAK3 were designed, which include an exchange of the aryl substituent attached to the piperazine ring, as shown in Figure 1. The designed derivatives (compounds **1**–**16**) were synthesised and then subjected to detailed *in vitro* and *in vivo* pharmacological evaluation, as well as structural characterisation with the use of molecular modelling and crystallographic techniques.

## Results and discussion

### Chemistry

**Scheme 1.**
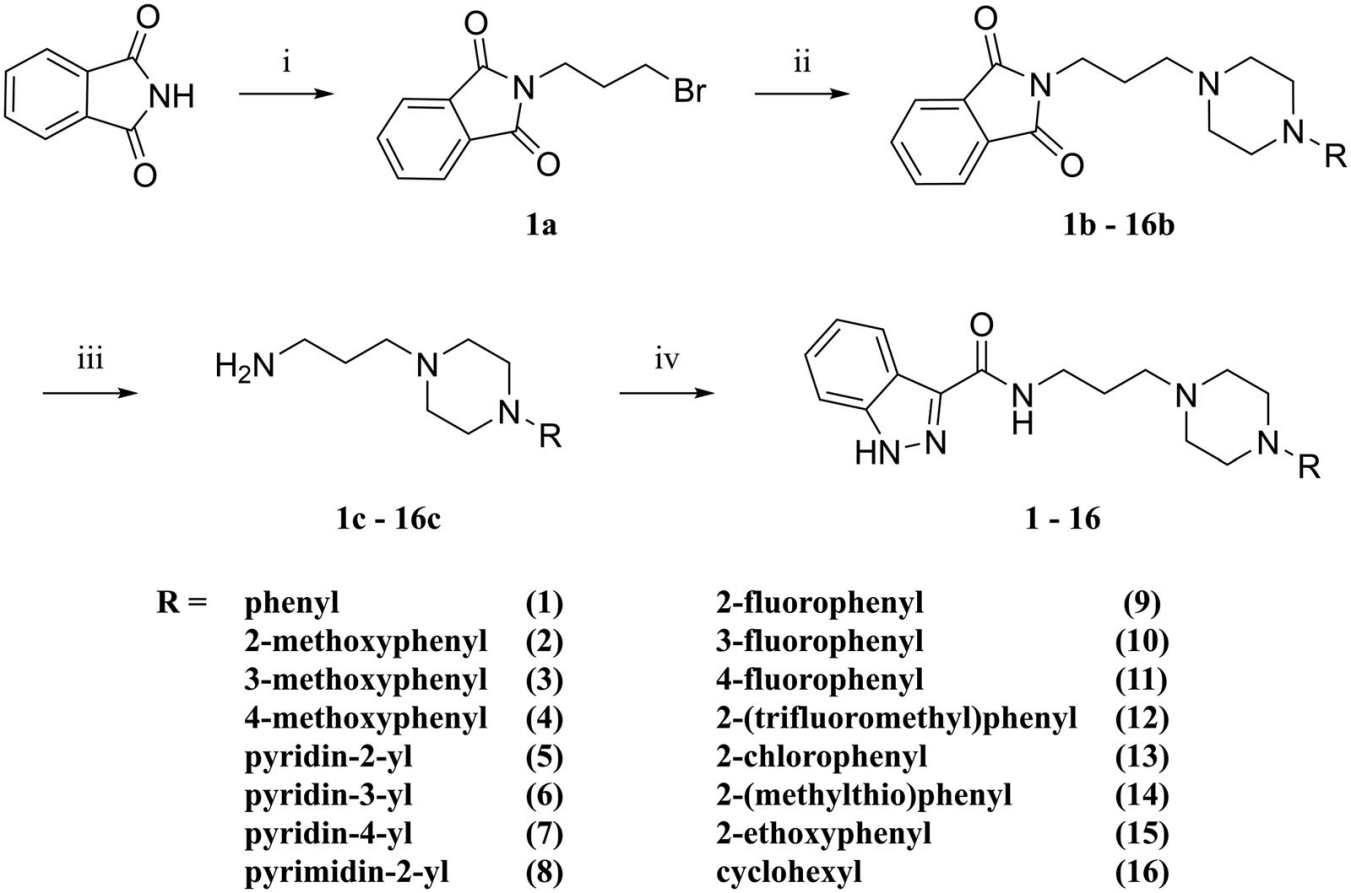
Synthetic route for compounds **1**–**16**. Reagents and conditions: (i) 1,3-dibromopropane, K_2_CO_3_, butan-2-one, reflux, 10 h; yield: 78%; (ii) *N*-substituted piperazine, K_2_CO_3_, KI_(cat.)_, ACN, reflux, 5–22 h; yield: 35–98%; (iii) NH_2_NH_2_·H_2_O, EtOH 96%, reflux, 3 h; yield: 18–95%; (iv) 1*H*-indazole-3-carboxylic acid, CDI, DMF, 60 °C, 2 h → 4.5 h; yield: 20–80%.

The designed derivatives of D2AAK3 (compounds **1**–**16**) were synthesised according to the synthetic pathway presented in Scheme 1. The final compounds have a secondary amide moiety, therefore in order to obtain them, the corresponding primary amines were first synthesised. For this purpose, the Gabriel synthesis was used^13^. Briefly, the phthalimide was first alkylated with 1,3-dibromopropane, and then the resulting intermediate was used to alkylate corresponding substituted piperazines. Subsequent hydrazinolysis yielded the desired primary amines, which then reacted with indazole-3-carboxylic acid to give the final compounds.

### Affinity of compounds at D_2_, 5-HT_1A_, and 5-HT_2A_ receptors and structure–activity relationship

**Figure 2.**
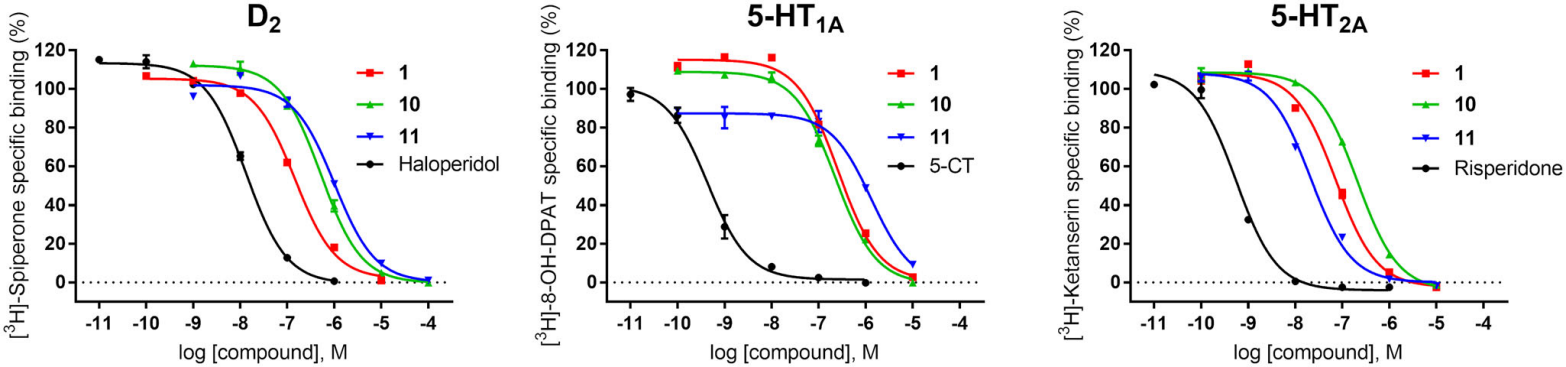
Competition radioligand binding curves for compounds **1**, **10**, and **11** and standard competitor ligands at human cloned D_2_, 5-HT_1A_, and 5-HT_2A_ receptors. The graphs show the data (mean ± SEM) of a representative experiment out of 2–5 (D_2_), 2–3 (5-HT_1A_), or 2 (5-HT_2A_) independent experiments performed in duplicate.

The compound series was evaluated in competition radioligand binding assays to determine the affinity of the compounds for dopamine D_2_ (D_2S_), serotonin 5-HT_1A_, and serotonin 5-HT_2A_ receptors. Experiments were carried out in membranes from cell lines stably expressing the human cloned receptors. A first screen at a single compound concentration of 10^−5^ M was followed by concentration–response curves for compounds showing % of inhibition of the specific radioligand binding over 60% in the first screen. Affinity values, expressed as p*K*_i_ and *K*_i_ (nM) or as % of inhibition of specific radioligand binding at 10 µM compound concentration (% inh.), are given in Table 1. Compounds for which the affinities are given, fully displaced the specific radioligand binding in a concentration-dependent manner in the range of concentrations assayed (from 10^−10^ or 10^−9^ M to 10^−5^ or 10^−4^ M). Figure 2 shows the competition radioligand binding curves for representative compounds **1**, **10**, and **11**, which were selected based on their affinities and D_2_/5-HT_2A_ activity ratios, at D_2_, 5-HT_1A_, and 5-HT_2A_ receptors, respectively, as well as for the corresponding reference standard haloperidol (D_2_), 5-carboxamidotryptamine (5-CT) (5-HT_1A_), and risperidone (5-HT_2A_). The competition radioligand binding curves for the remaining compounds may be found in Supplementary Information (Figs. S1, S2, and S3).

**Table 1.** Competitive radioligand binding data at human D_2_, 5-HT_1A_, and 5-HT_2A_ receptors.

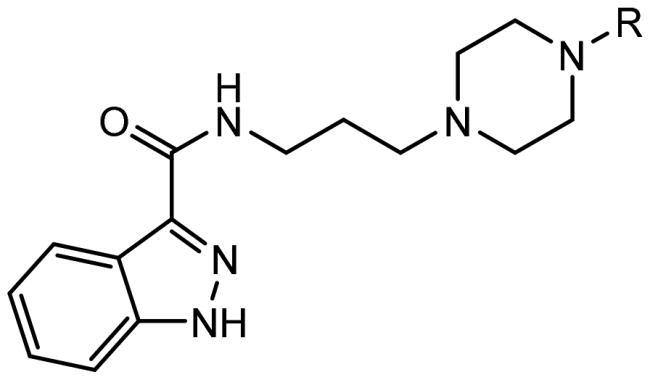					
Compound	R	p*K*_i_ (*K*_i_, nM) or % inh. at 10 μM			p*K*_i_ 5-HT_2A_/p*K*_i_ D_2_
		D_2_	5-HT_1A_	5-HT_2A_	
**1**	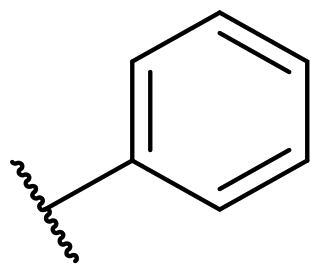	7.16 ± 0.02 (69.0)	6.81 ± 0.08 (155)	7.45 ± 0.02 (35.6)	1.04
**2**	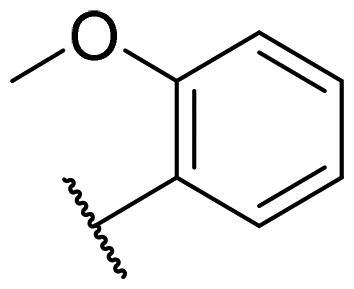	7.64 ± 0.02 (22.7)	7.64 ± 0.03 (22.9)	6.43 ± 0.04 (372)	0.84
**3**	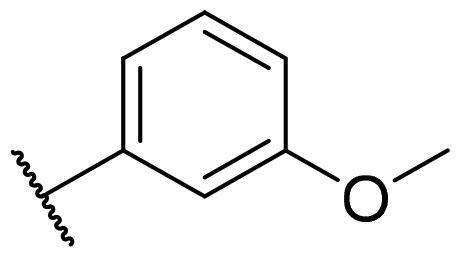	6.50 ± 0.01 (315)	6.96 ± 0.06 (110)	6.74 ± 0.08 (184)	1.04
**4**	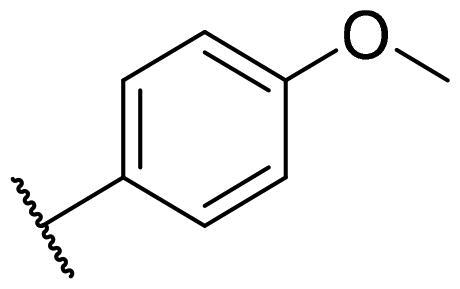	46 ± 6%	58.4 ± 1.8%	6.31 ± 0.05 (491)	ND
**5**	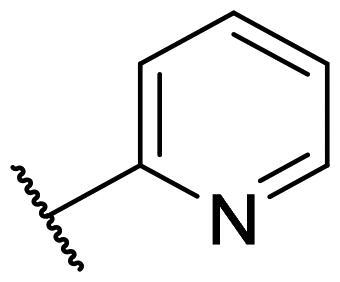	6.47 ± 0.06 (339)	6.95 ± 0.03 (113)	6.88 ± 0.03 (132)	1.06
**6**	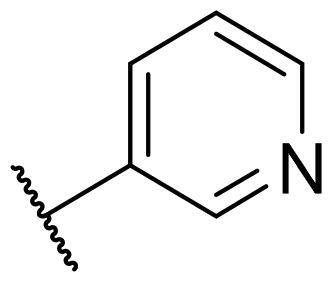	5.99 ± 0.06 (1014)	5.86 ± 0.01 (1384)	5.58 ± 0.04 (2649)	0.93
**7**	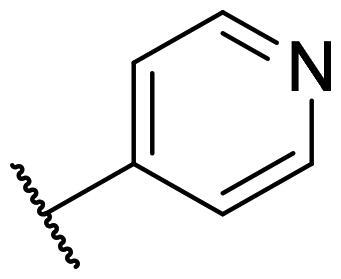	0%	27.9 ± 2.7%	0%	ND
**8**	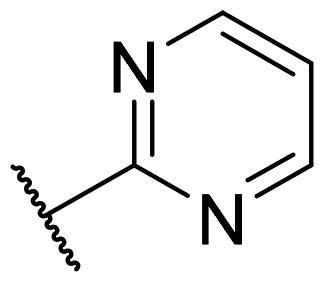	6.32 ± 0.01 (480)	6.62 ± 0.05 (238)	6.09 ± 0.04 (807)	0.96
**9**	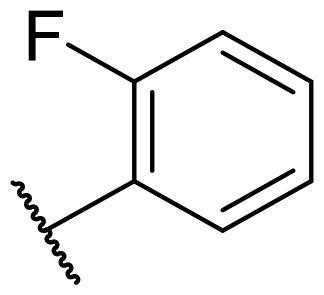	7.38 ± 0.06 (42.2)	6.87 ± 0.04 (135)	6.93 ± 0.02 (117)	0.94
**10**	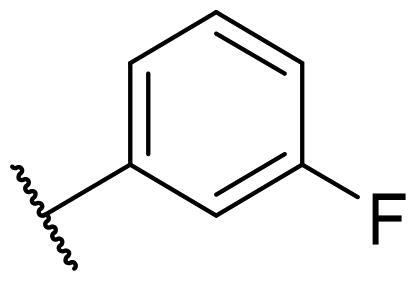	6.55 ± 0.09 (284)	6.95 ± 0.01 (114)	7.03 ± 0.07 (94.4)	1.07
**11**	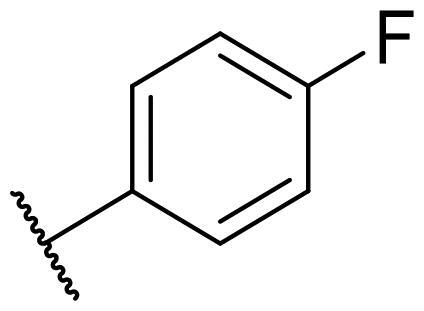	6.35 ± 0.03 (450)	6.22 ± 0.04 (607)	7.90 ± 0.07 (12.6)	1.24
**12**	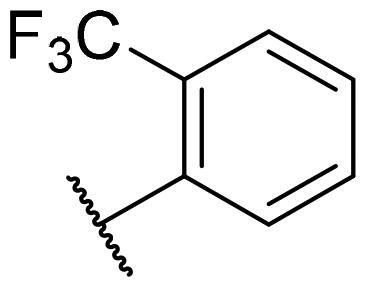	7.35 ± 0.05 (45.2)	6.37 ± 0.01 (423)	6.08 ± 0.08 (832)	0.83
**13**	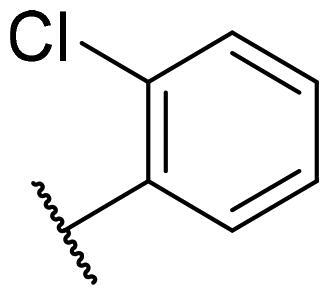	7.16 ± 0.15 (69.7)	6.97 ± 0.05 (107)	6.87 ± 0.01 (134)	0.96
**14**	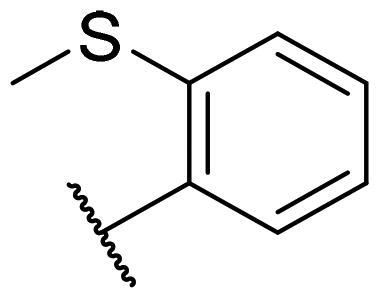	8.15 ± 0.06 (7.05)	7.91 ± 0.04 (12.4)	6.66 ± 0.01 (217)	0.82
**15**	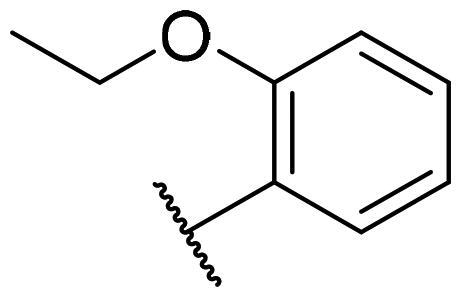	7.70 ± 0.07 (19.8)	7.33 ± 0.03 (46.9)	6.55 ± 0.00 (280)	0.85
**16**	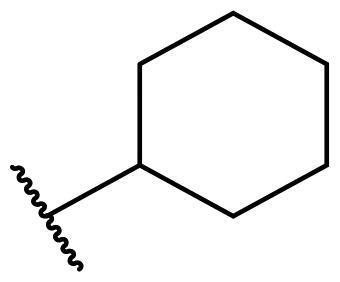	19.0 ± 6.8%	56.7 ± 1.6%	25.6 ± 9.0%	ND
D2AAK3		6.94 ± 0.06 (115)	7.34 ± 0.14 (51.4)	6.88 ± 0.23 (151)	0.99
Haloperidol		8.13 ± 0.05 (7.41) (*n* = 11)			
5-CT			9.59 ± 0.07 (0.26) (*n* = 5)		
Risperidone				9.65 ± 0.04 (0.23) (*n* = 5)	

ND: not determined.

Data are expressed as % inh. at 10 μM, or p*K*_i_ and *K*_i_ (nM) when full displacement was achieved (mean ± SEM of 2–5 for D_2_, 2–3 for 5-HT_1A_, or 2–5 for 5-HT_2A_ independent experiments performed in duplicate).

The binding affinities of the synthesised compounds were analysed in terms of the influence of the introduction of various substituents in the aryl part of the piperazine moiety on the activity of these derivatives towards dopamine D_2_, and serotonin 5-HT_1A_ and 5-HT_2A_ receptors.

The most preferred modification affecting binding to the dopamine D_2_ receptor is the introduction of a substituent at the *ortho* position of the phenyl ring (**2**, **9**, **12**, **13**, **14**, and **15** vs. **1**). Electron-donating substituents such as ethers (**2** and **15**) and thioethers (**14**) appear to contribute more to the increase in activity than electron-withdrawing substituents such as halogens (**9** and **13**) and trifluoromethyl (**12**). In particular, the installation of the methylthio substituent at the *ortho* position of the phenyl leads to a strong binding to the receptor, which is about three times higher than that of the methoxy and ethoxy groups at the same position (**14** vs. **2** and **15**). Clearing the phenyl of any substituents results in a similar activity to the *ortho* positioning of the electron-withdrawing substituent (**1** vs. **9**, **12** and **13**). Shifting the substituents on the phenyl ring from *ortho* to *meta* positions results in a decrease in activity, but with a reversed tendency compared to *ortho* substitution–trifluoromethyl and fluoro substituents contribute to a higher affinity than electron-donating groups (D2AAK3 and **10** vs. **2**). *Para* positioning of the substituents is the least favourable and leads to an even greater decrease in binding affinity compared to *meta* substitution (**4** and **11** vs. **3** and **10**), and almost a loss of activity in the case of installing a larger substituent such as methoxy group (**4**). Replacing the phenyl ring with a heterocyclic system containing nitrogen atoms is generally disadvantageous (**5**, **6**, **7**, and **8** vs. **1**). While the introduction of the pyridin-2-yl and pyrimidin-2-yl groups maintains moderate activity (**5** and **8** vs. **1**), the substitution of the C3 carbon with a nitrogen atom leads to a drastic decrease in activity (**6** vs. **1**), and its shift to the C4 carbon causes the compound to become completely inactive (**7** vs. **1**).

Similar tendencies can be noticed in the case of the influence of the introduced substituents on the activity towards the serotonin 5-HT_1A_ receptor. Compounds that have *ortho*-phenyl electron-donating substituents (**2**, **14**, and **15** vs. **1**), especially a methylthio substituent (**14**), are the most active. The introduction of electron-withdrawing substituents into this position leads to a decrease in binding affinity (**9**, **12**, and **13** vs. **1**), this effect being most pronounced in the case of the strong electron-withdrawing substituent, i.e. trifluoromethyl (**12**). *Meta* substitution of the phenyl ring similarly affects activity as with *ortho* substituents (**3** and **10** vs. **2** and **9**), while *para* substitution (**4** and **11**), as in the case of D_2_ receptor, causes a decrease in activity or even its loss with the introduction of a larger substituent, in this case methoxy (**4**). The presence of a heterocyclic nitrogen atom in an aryl system influences the activity of the compounds in a similar way as in the case of D_2_ receptor (**5**, **6**, **7**, and **8** vs. **1**). Replacing the C2 carbon atom with nitrogen leads to a compound activity comparable to the introduction of an *ortho* or *meta*-substituted phenyl group (**5** vs. **2**, **9**, **12**, **13**, **14**, and **15** vs. **3** and **10**). Nitrogen in the C3 position (**6** vs. **1**) leads to several times lower binding affinity, and in the C4 position to its loss (**7** vs. **1**).

In the case of the serotonin 5-HT_2A_ receptor, the introduction of a fluorine atom into the phenyl ring has the greatest beneficial effect on the activity of the compound (**9**, **10**, and **11** vs. **1**). The compound in which the fluorine is located in the *para*-phenyl position (**11**) shows the highest affinity. The shift of the fluorine atom to the *meta* position causes an almost eightfold reduction in activity (**10** vs. **11**), and to the *ortho* position almost 10-fold (**9** vs. **11**). Interestingly, the lack of substitution in the phenyl ring leads to a much higher activity compared to the *meta* and *ortho* substitution with fluorine (**1** vs. **10** and **9**), and only three times decreased activity than with the *para*-phenyl fluorine introduction (**1** vs. **11**). A similar effect to the installation in the *ortho* position of the fluorine atom has the introduction of the chlorine atom as well as the replacement of C2 carbon with nitrogen (**9** vs. **13** and **5**). The size of the substituent at the *ortho* and *meta*-phenyl positions appears to have a significant effect on the binding affinity, as is seen in the case of ether and thioether substituents, which are less preferred than the halogens at these positions (**2**, **15**, and **14** vs. **9** and **13**). As with the D_2_ and 5-HT_1A_ receptors, introducing a pyridin-3-yl substituent results in a compound with low activity towards 5-HT_2A_ receptor (**6** vs. **1**), whereas installing a pyridin-4-yl substituent leads to an inactive compound (**7** vs. **1**).

For each of the above receptors, replacement of the aryl substituent at the 4-position of the piperazine with an alicyclic substituent, in this case cyclohexyl (**16** vs. **1**), results in a lack of affinity for these receptors, indicating that the preservation of the aromatic system at this site is necessary for obtaining the desired activity.

### Extended affinity profiling of selected compounds over additional GPCRs of interest

Compounds **1**, **10**, and **11** were selected from the series for an extension of their affinity profiling at dopaminergic D_1_ and D_3_, and serotonergic 5-HT_7_ receptors. Compounds **1** and **10** showed D_1_ receptor affinities consistent with their improved dopaminergic D_2_ receptor affinity compared to D2AAK3, while preserving D_3_ and 5-HT_7_ receptors affinities in the high nanomolar range (Table 2). Compound **11** displayed improved 5-HT_7_ receptor affinity compared to D2AAK3, a profile that might favour benefits in cognition and mitigate metabolic side effects^14^. The selectivity of the three selected compounds over histaminergic and muscarinic receptors was also investigated, which revealed low affinity of the three compounds for human H_1_ receptors (*K*_i_ values in the high nanomolar to micromolar range) and lack of affinity for human M_1_ receptors (Table 2).

**Table 2.** Competitive radioligand binding data for compounds **1**, **10**, and **11** at human D_1_, D_3_, 5-HT_7_, H_1_, and M_1_ receptors.

Compound	p*K*_i_ (*K*_i_, nM) or % inh. at 10 μM				
	D_1_	D_3_	5-HT_7_	H_1_	M_1_
**1**	6.62 ± 0.08 (241)	6.15 ± 0.00 (711)	6.27 ± 0.00 (540)	6.32 ± 0.01 (474)	21 ± 6%
**10**	6.74 ± 0.08 (181)	6.14 ± 0.03 (719)	6.23 ± 0.06 (589)	6.00 ± 0.07 (1002)	0%
**11**	(6.92 ± 0.05) (119)	5.30 ± 0.01 (5012)	6.71 ± 0.03 (196)	6.44 ± 0.05 (364)	0%
Haloperidol	8.31 ± 0.01 (4.95)	8.24 ± 0.06 (5.81)			
Clozapine			7.59 ± 0.04 (26.0)		
Doxepin				9.07 ± 0.07 (0.86)	
Ipratropium					8.68 ± 0.01 (2.11)

Data (mean ± SEM of two independent experiments performed in duplicate) are expressed as % inh. at 10 μM, or p*K*_i_ and *K*_i_ (nM) when full displacement was achieved.

### Efficacy of selected compounds at D_2_, 5-HT_1A_, and 5-HT_2A_ receptors

**Figure 3.**
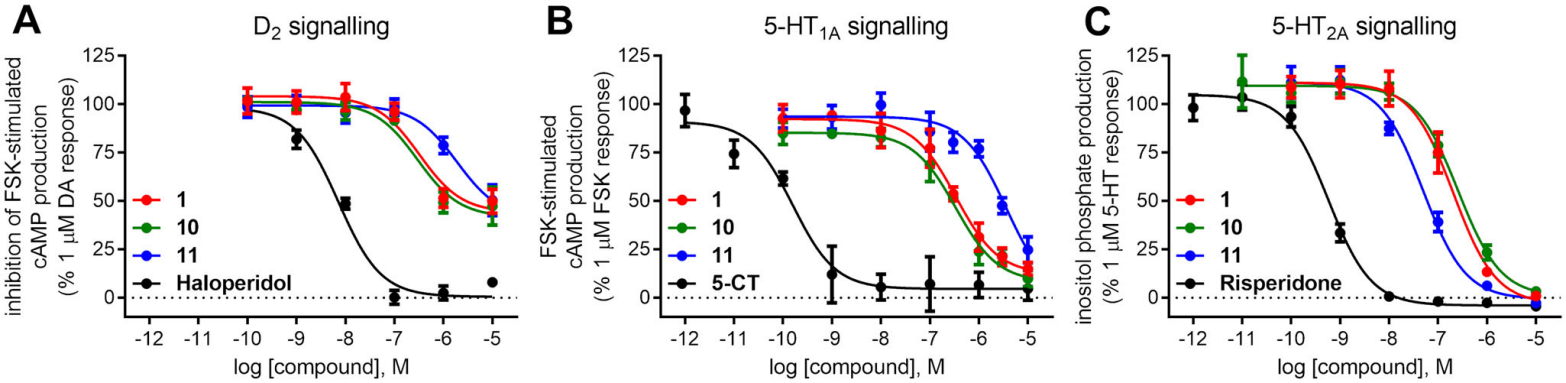
Concentration–response curves of compounds **1**, **10**, **11**, and reference ligands haloperidol (as D_2_ antagonist), 5-CT (as 5-HT_1A_ agonist), and risperidone (as 5-HT_2A_ antagonist) in functional assays of cAMP signalling (D_2_, 5-HT_1A_) and IP production (5-HT_2A_). Data are expressed as % of 1 µM dopamine (DA) response (A), % of 1 µM forskolin (FSK)-stimulated cAMP production (B), and % of 1 µM serotonin (5-HT) response (C). The graph shows data (mean ± SEM) of 2–3 (D_2_, 5-HT_1A_) or 4–5 (5-HT_2A_) independent experiments performed in duplicate.

Compounds **1**, **10**, **11**, and reference D_2_ antagonist haloperidol inhibited 1 µM dopamine response in functional assays of cAMP signalling in CHO-K1 cells expressing human dopamine D_2_ receptors (Figure 3(A)). The % of inhibition of 1 µM dopamine response achieved by compounds **1**, **10**, and **11** at 1 µM concentration was 48.5 ± 4.9%, 50.8 ± 5.4%, and 21.3 ± 4.3%, respectively (Figure 3(A)). These results indicate antagonistic efficacy of the three compounds at dopamine D_2_ receptors in agreement with their rank of affinities for this receptor target. Increasing the concentration of the compound to 10 µM (the highest concentration assayed in functional cell-based assays to avoid interference of vehicle), further increased the % of inhibition of dopamine response achieved by the three selected compounds, although full inhibition was not achieved in these assay conditions (Figure 3(A), Table 3). Due to this limitation, only an estimation of the potency of compounds **1**, **10**, and **11** was extracted from these assays, expressed as p*K*_b_ values (–log of the equilibrium dissociation constant of a competitive antagonist determined by means of a functional assay, *K*_b_) (Table 3).

**Table 3.** Potency and efficacy of selected compounds at D_2_, 5-HT_1A_, and 5-HT_2A_ receptor signalling.

	D_2_ antagonism (cAMP signalling)			5-HT_1A_ agonism (cAMP signalling)			5-HT_2A_ antagonism (IP signalling)		
Compound	p*K*_b_	*K*_b_ (nM)	%inh. of DA response	pEC_50_	EC_50_ (nM)	%inh. of FSK response	p*K*_b_	*K*_b_ (nM)	%inh. of 5-HT response
**1**	7.65 ± 0.09	22.5	50.3 ± 6.3%	6.49 ± 0.09	324 ± 69	85.1 ± 3.3%	7.10 ± 0.11	80.2 ± 20.1	99.1 ± 2.5%
**10**	7.51 ± 0.15	31.2	52.6 ± 9.8%	6.45 ± 0.10	356 ± 83	90.2 ± 4.1%	7.00 ± 0.13	101 ± 31	96.8 ± 2.0%
**11**	7.00 ± 0.11	99.1	49.7 ± 8.0%	5.25 ± 0.09	5598 ± 1196	75.3 ± 6.8%	7.72 ± 0.16	19.2 ± 7.2	102.7 ± 2.2%
Haloperidol	9.53 ± 0.07	0.29	92.1 ± 0.6%	n.d.	n.d.	n.d.	n.d.	n.d.	n.d.
5-CT	n.d.	n.d.	n.d.	9.57 ± 0.19	0.27 ± 0.10	95.3 ± 6.0%	n.d.	n.d.	n.d.
Risperidone	n.d.	n.d.	n.d.	n.d.	n.d.	n.d.	9.77 ± 0.09	0.17 ± 0.03	104.5 ± 1.3%

n.d.: not determined.

Compounds were evaluated in cell-based functional assays of second messenger production (inhibition of forskolin-stimulated cAMP signalling for D_2_ and 5-HT_1A_ receptors, stimulation of IP production for 5-HT_2A_ receptors) in cell lines stably overexpressing the cloned human receptors. D_2_ antagonistic activity of the compounds was quantified as inhibition of 1 µM dopamine (DA) response; 5-HT_1A_ agonistic activity was quantified as inhibition of 1 µM forskolin (FSK)-stimulated cAMP production; and 5-HT_2A_ antagonistic activity was quantified as inhibition of 1 µM serotonin (5-HT) response. The table shows potency values, expressed as p*K*_b_ (–log*K*_b_) and *K*_b_ (nM) for antagonists and as pEC_50_ (–logEC_50_) and EC_50_ (nM) for agonists, as well as efficacy values at 10 µM concentration of the compounds, expressed as % of inhibition (%inh.) of the indicated response. *K*_b_ values of antagonists were calculated from the inhibition concentration–response curves of the compounds against a single agonist concentration of 1 µM. Average EC_50_ values for DA and 5-HT in these assays were 116 nM and 569 nM, respectively (pEC_50_ DA (mean ± SEM) = 6.94 ± 0.16; pEC_50_ 5-HT (mean ± SEM) = 6.25 ± 0.06). Data shown in the table are mean ± SEM of 2–3 (D_2_, 5-HT_1A_) or 4–5 (5-HT_2A_) independent experiments performed in duplicate.

As for serotonin 5-HT_1A_ receptor, compounds **1**, **10**, and **11** and reference 5-HT_1A_ agonist 5-CT inhibited the forskolin-stimulated cAMP production in a concentration-dependent manner in HEK293 cells stably expressing 5-HT_1A_ receptors (Figure 3(B), Table 3), indicative of their agonistic activity towards 5-HT_1A_ receptor.

Compounds **1**, **10**, **11**, and reference 5-HT_2A_ antagonist risperidone inhibited 1 µM serotonin response in a concentration-dependent manner in functional assays of inositol phosphate (IP) production in CHO-K1 cells stably expressing the cloned human 5-HT_2A_ receptor (Figure 3(C), Table 3), achieving the three selected compounds full inhibition of 1 µM serotonin response at the maximal concentration assayed (10 µM) (Figure 3(C), Table 3). Potency values of the compounds extracted from these assays, expressed as p*K*_b_ and *K*_b_ (nM), showed good agreement with the rank of 5-HT_2A_ affinity of the compounds (Figure 3(C), Table 3).

### Molecular modelling

**Figure 4.**
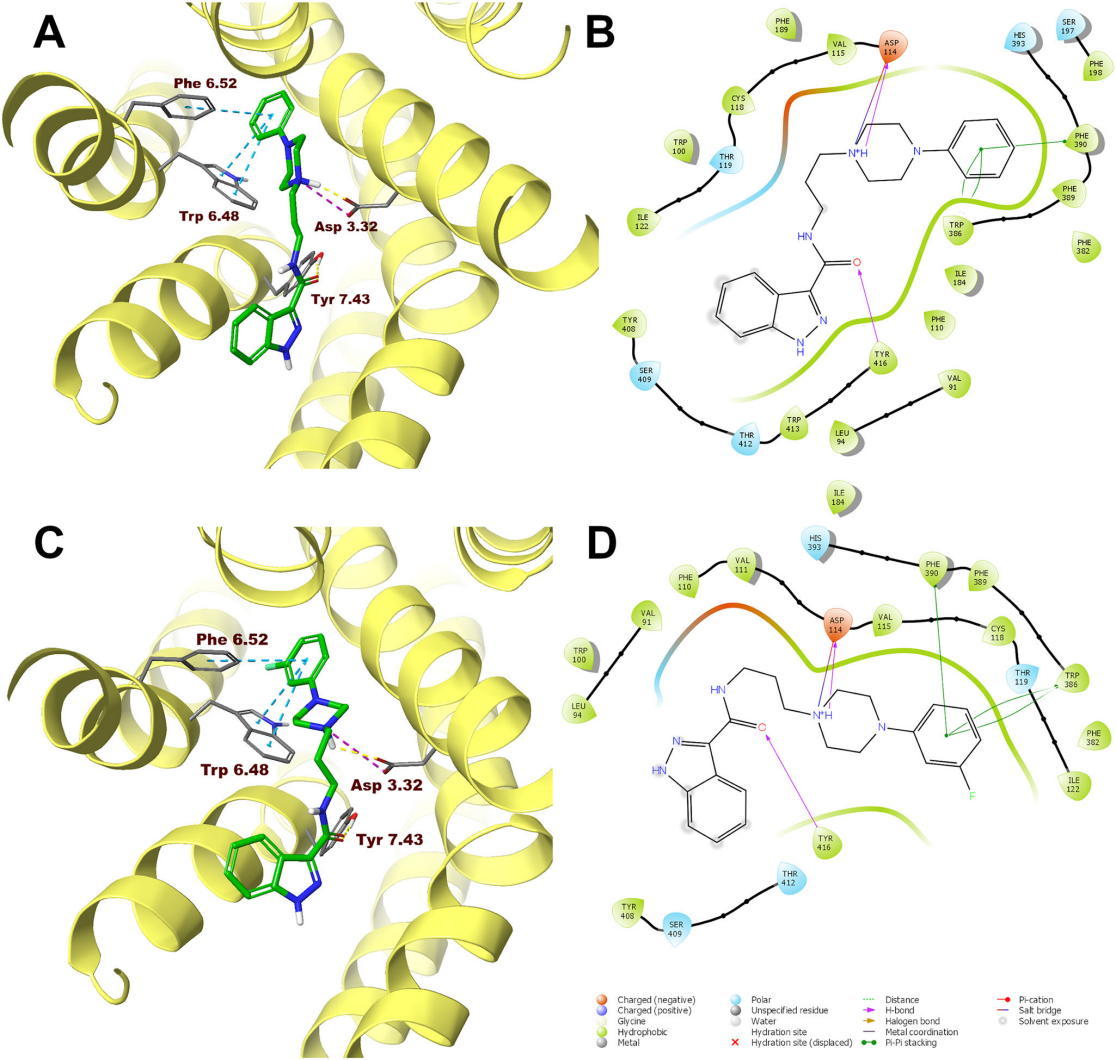
Compounds **1** (A, B) and **10** (C, D) in the binding pocket of the human dopamine D_2_ receptor. (A, C) 3D view of the binding site. Ligands are represented as sticks with green carbon atoms. Protein is represented as yellow ribbons, main interacting residues are shown as sticks with grey carbon atoms. Electrostatic interactions are shown as pink dashed lines, hydrogen bonds as yellow dashed lines, π–π stacking as light blue dashed lines. Non-polar hydrogen atoms were omitted for clarity. (B, D) 2D view of the binding site.

**Figure 5.**
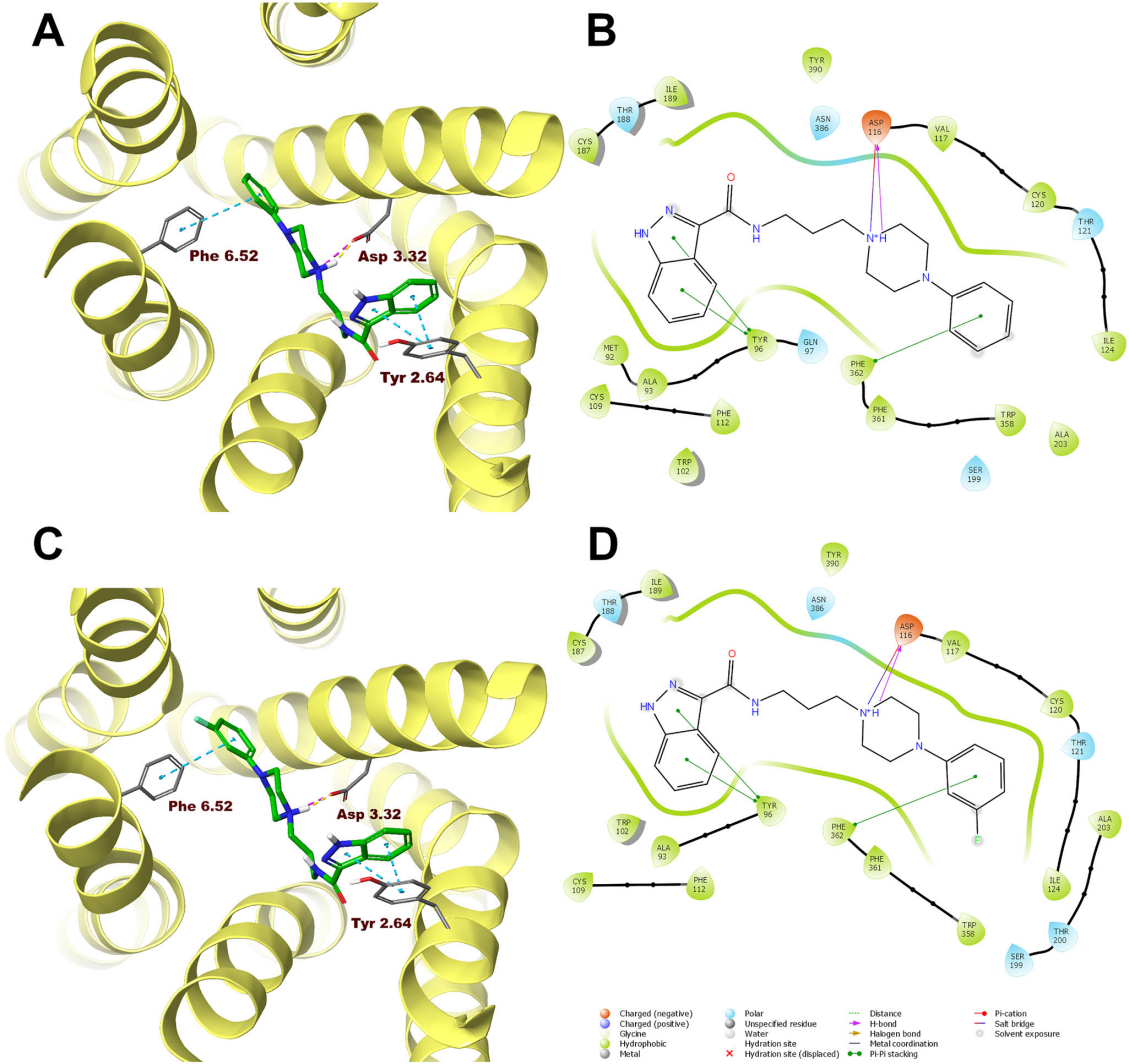
Compounds **1** (A, B) and **10** (C, D) in the binding pocket of the human serotonin 5-HT_1A_ receptor. (A, C) 3D view of the binding site. Ligands are represented as sticks with green carbon atoms. Protein is represented as yellow ribbons, main interacting residues are shown as sticks with grey carbon atoms. Electrostatic interactions are shown as pink dashed lines, hydrogen bonds as yellow dashed lines, π–π stacking as light blue dashed lines. Non-polar hydrogen atoms were omitted for clarity. (B, D) 2D view of the binding site.

**Figure 6.**
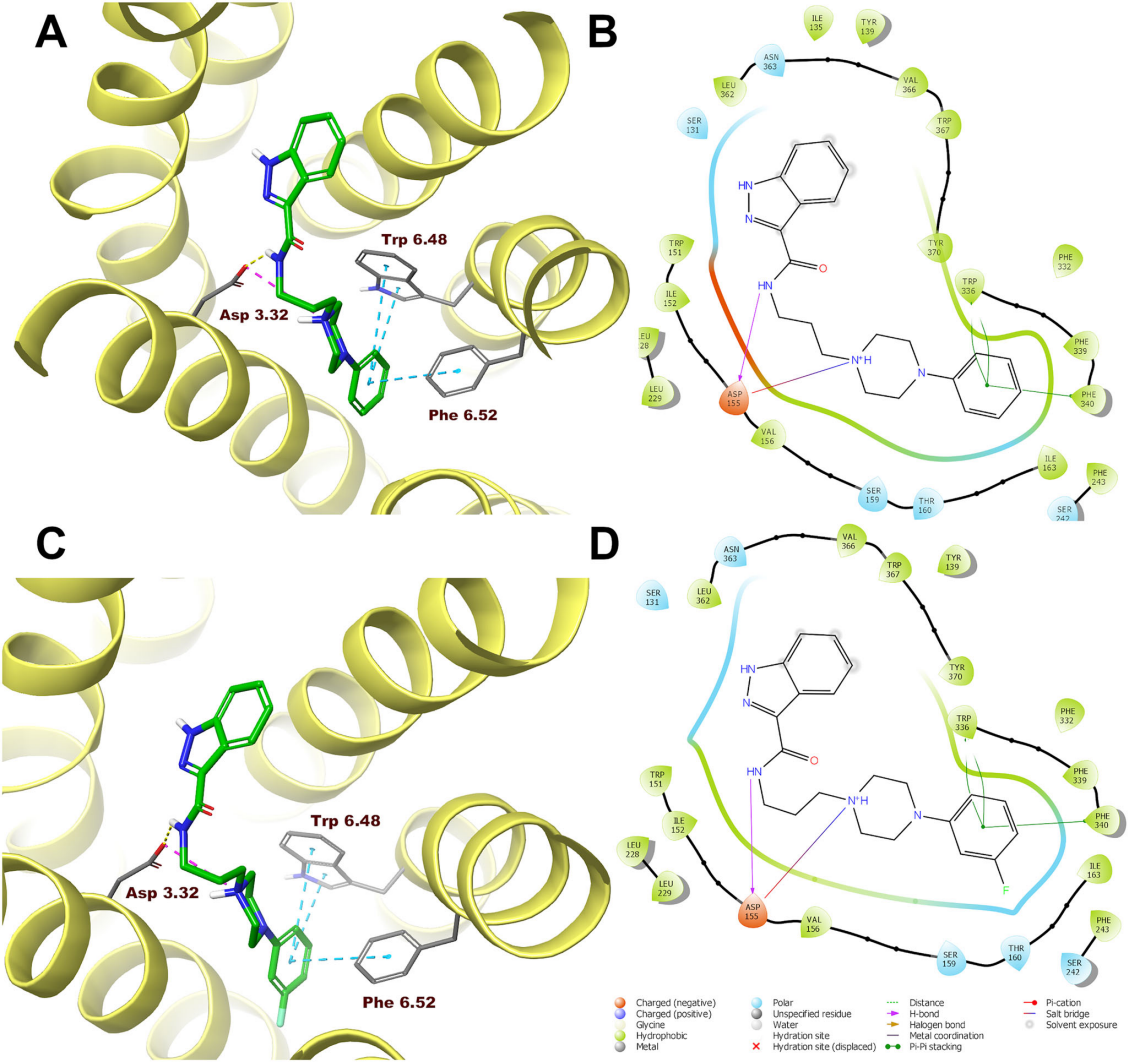
Compounds **1** (A, B) and **10** (C, D) in the binding pocket of the human serotonin 5-HT_2A_ receptor. (A, C) 3D view of the binding site. Ligands are represented as sticks with green carbon atoms. Protein is represented as yellow ribbons, main interacting residues are shown as sticks with grey carbon atoms. Electrostatic interactions are shown as pink dashed lines, hydrogen bonds as yellow dashed lines, π–π stacking as light blue dashed lines. Non-polar hydrogen atoms were omitted for clarity. (B, D) 2D view of the binding site.

In order to analyse ligand–protein interactions for the designed and synthesised compounds and to study their binding mode with given molecular targets, molecular docking was performed. Compounds **1**–**16**, after appropriate preparation, were docked to the binding pockets of the D_2_ and 5-HT_2A_ receptors in the inactive state, and the 5HT_1A_ receptor in the active state. The obtained compounds were designed to show antagonism to the D_2_ and 5-HT_2A_ receptors, and agonism to the 5-HT_1A_ receptor, which for some of them was confirmed in functional studies, and on this basis, the above receptor conformations were chosen. Compounds **1** and **10**, which were selected for detailed behavioural evaluation, and their interactions with the D_2_, 5-HT_1A_, and 5-HT_2A_ receptors are shown in Figures 4–6, respectively. The structures of these compounds contain features characteristic of a typical pharmacophore model for aminergic GPCRs; hence, the main site of contact of studied ligands with orthosteric binding sites of the receptors is the electrostatic interaction between the protonatable nitrogen atom of the ligand and aspartic acid located in the third helix of the receptor–Asp 3.32 (according to the Ballesteros–Weinstein nomenclature^15^).

Both compounds **1** and **10** show a very similar arrangement in the binding sites of the D_2_, 5-HT_1A_, and 5-HT_2A_ receptors; therefore, in addition to interacting with Asp 3.32, they interact with the same amino acid residues of the binding pockets. In the case of the D_2_ receptor, there are π–π stacking interactions between the phenyl group attached to the piperazine and the aromatic systems Phe 6.52 and Trp 6.48. In addition, the hydroxyl group present in Tyr 7.43 forms a hydrogen bond with the carbonyl group of the ligands. Considering the 5-HT_1A_ receptor, π–π stacking interactions of the phenyl group of the ligands with Phe 6.52 are also observed, and in addition interactions of the same type between the indazole fragment and Tyr 2.64. Similarly, in the binding pocket of the 5-HT_2A_ receptor, the tested ligands are also stabilised by interactions of the π–π stacking type between aryl substituent at the piperazine moiety and the side chains of the aromatic amino acids Phe 6.52 and Trp 6.48.

In the case of the D_2_ receptor, the designed compounds show different conformations in the binding pocket depending on the position of the substituent on the phenyl ring of the arylpiperazine. Compounds having a substituent in the *ortho* position of this system adopt a position in which the indazole moiety faces the interior of the receptor. This conformation seems to be preferred for ligands with high affinity for the D_2_ receptor as these *ortho*-substituted derivatives show the highest activity at this receptor of the series. Also, the *meta*- and *para*-methoxy derivatives are arranged in a similar way at the binding site; however, this is probably due to the size of the substituent being too large, as these compounds have a lower affinity for the D_2_ receptor than their *ortho*-substituted analogues. The remaining compounds in the binding pocket adopt a conformation similar to the previously reported compound D2AAK3, with the indazole moiety facing the outside of the receptor. At the 5-HT_1A_ receptor binding site, most of the docked compounds, especially those with the highest affinity for this receptor, are arranged similarly to D2AAK3, with the indazole moiety facing the extracellular space. However, unlike D2AAK3, they are arranged shallower in the binding pocket and deviated from the direction of the helices, in some cases, even arranged almost perpendicular to this direction. The arrangement of docked compounds at the 5-HT_2A_ receptor binding site is more consistent throughout the series than with previous receptors. In this case, most of the compounds are arranged in such a way that the arylpiperazine group faces the inside of the receptor, what is consistent with the previously reported binding pose of D2AAK3.

Compounds **1**–**16** were also filtered against pan-assay interference compounds (PAINS). Compound **4** was caught by PAINS3 filter, while all other compounds passed all filters.

### X-ray studies

**Figure 7.**
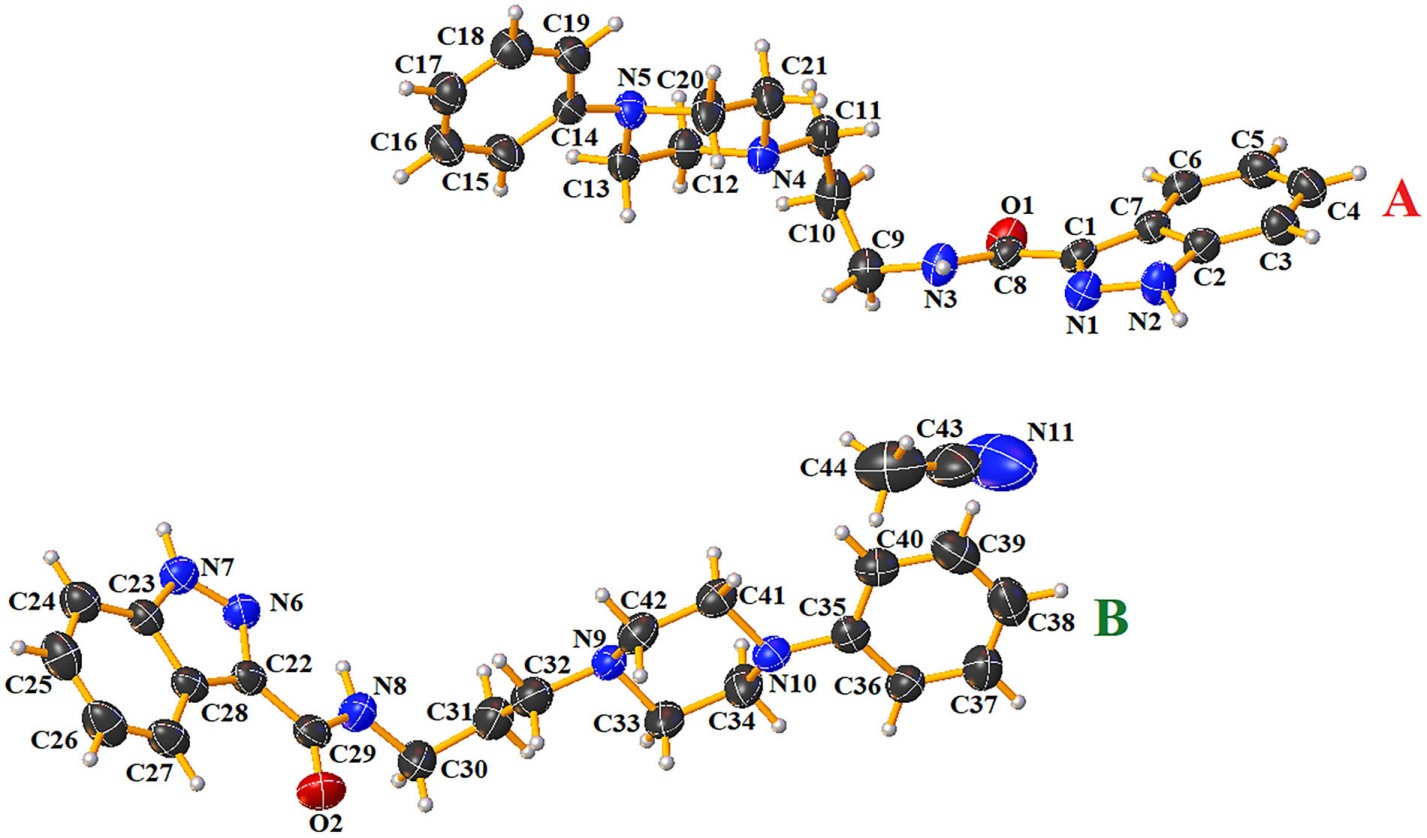
Molecular structure of compound **1** with atoms and molecules labelling scheme.

After recrystallisation of compound **1** from acetonitrile, crystals of sufficient quality suitable for X-ray analysis were obtained. This compound crystallises in the centrosymmetric triclinic space group *P*$\bar{1}.$ The molecular structure of compound **1** is illustrated in Figure 7. The asymmetric unit consists of two independent molecules **A** and **B** of *N*-(3-(4-phenylpiperazin-1-yl)propyl)-1*H*-indazole-3-carboxamide and one molecule of acetonitrile solvent. In the Cambridge Structural Database (CSD version 5.43 with updates November 2022)^16^, there is no similar compound building with the *N*-substituted-1*H*-indazole-3-carboxamide and 4-phenylpiperazine units. Bond distances and angles are in the expected ranges^17^ and agree with values reported for compounds containing similar building units^18–25^. Interatomic distances and selected bond angles for compound **1** are given in Table S1 (Supplementary Information). The molecules **A** and **B** of compound **1** are non-planar. The dihedral angles between the planes formed by non-hydrogen atoms of the 1*H*-indazole-3-carboxamide fragment and 1-propyl-4-phenylpiperazine unit are 61.01(8)° and 77.78(6)° for **A** and **B**, respectively. The molecules **A** and **B** are connected via N − H···N hydrogen bonds in homodimers (Table 4, Figure S4 in Supplementary Information). The molecules **A** are linked together by N(2)−H(2N)···N(4) and N(3)–H(3N)···N(1), which generate $R_{2}^{2}(20)$ and $R_{2}^{2}(10)$ rings motifs^26^. In the case of two molecules, B the intermolecular N(7)-H(7N)···N(9) hydrogen bonds form a $R_{2}^{2}(20)$ ring motif^26^. The acetonitrile is linked to molecule **B** by weak C(24)–H(24)···N(11) interaction. The piperazine rings display a distorted chair conformation, as indicated by the following respective puckering parameters: *Q* = 0.563(3)°, *θ* = 4.7(3)°, *φ* = 357(5)° for **A** and *Q* = 0.571(4)°, *θ* = 176.6(32)°, *φ* = 180(6)° for **B** molecules^27–29^.

**Table 4.** Hydrogen bonding geometry (Å, °) for compound **1**.

D-H⋯A	d(D-H)	d(H···A)	d(D···A)	∠DHA
N(2)-H(2N)⋯N(4)^i^	0.98(3)	1.97(3)	2.928(4)	163(3)
N(3)-H(3N)⋯N(1)^i^	0.83(3)	2.42(3)	3.179(4)	152(2)
N(7)-H(7N)⋯N(9)^ii^	0.92(3)	1.93(4)	2.846(4)	169(3)
C(24)-H(24)⋯N(11)^iii^	0.93	2.53	3.351(6)	147
C(34)-H(34A)⋯O(1)^iv^	0.97	2.41	3.317(4)	156
C(42)-H(42A)⋯O(1)^v^	0.97	2.49	3.455(4)	176

Symmetry codes: (i) –*x*, –*y*+1, –*z*+1; (ii) –*x*+2, –*y*, –*z*; (iii) *x*+1, *y*–1, *z*–1; (iv) –*x*+1, –*y*+1, –*z*+1; (v) *x*+1, *y*–1, *z*.

As mentioned above, the presence of N–H···N hydrogen bonds joins the molecules into homodimers A–A and B–B (Figure S4 in Supplementary Information). Additionally, the weak C–H···O interaction occurs in the structure. As can be seen in Figure S5 (Supplementary Information), these interactions link molecules in layers.

### Behavioural studies

#### Effects of compound 1 and compound 10 on mice’ spontaneous locomotor activity

Animals’ mobility is an important variable that can affect the interpretation of the observed results. Therefore, in order to evaluate the impact of studied compounds on animals’ locomotor activity, an automated activity tracker has been used and animals’ locomotion has been recorded for 30 min (30 min after administered treatment).

**Figure 8.**
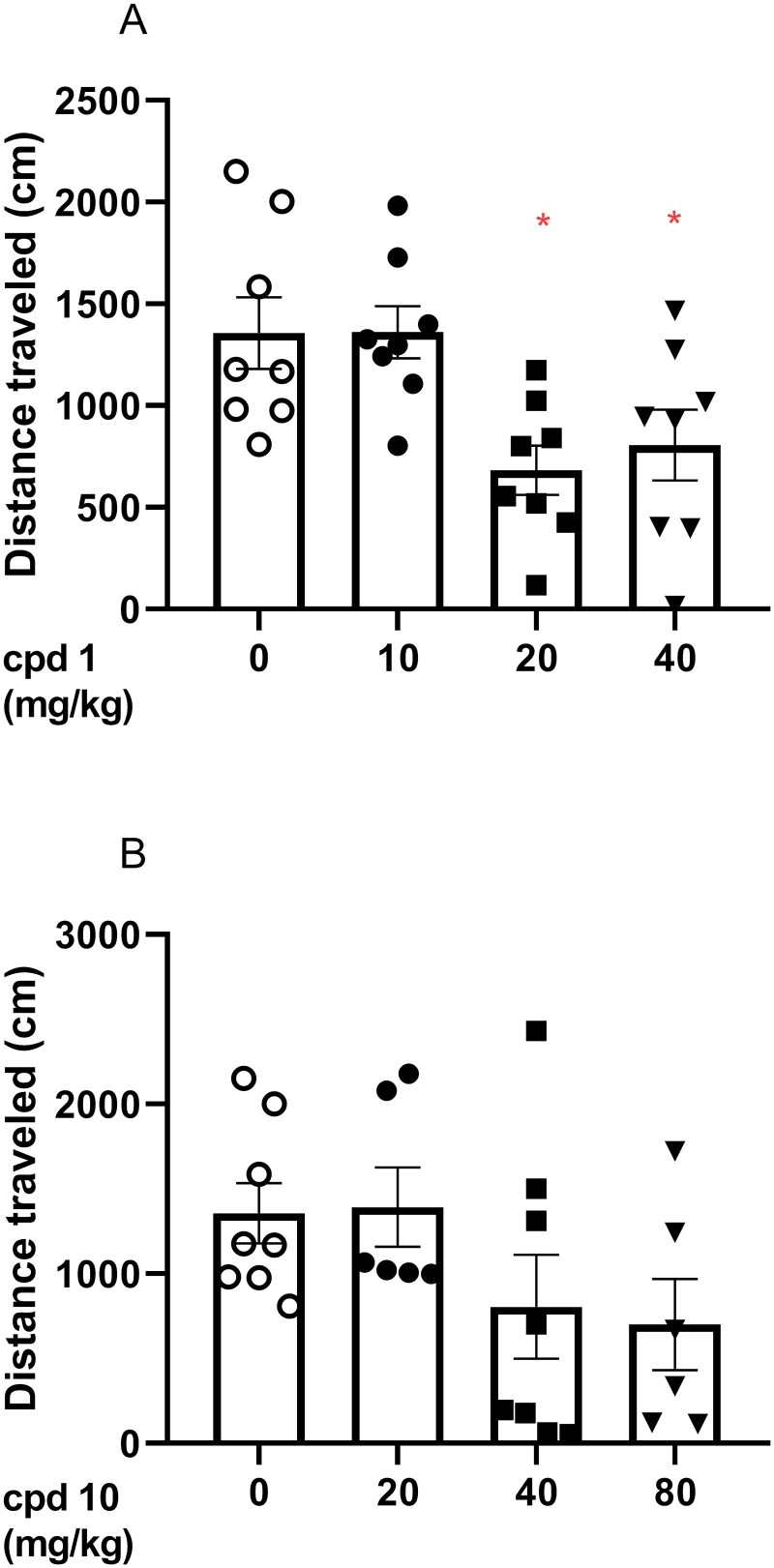
Acute effects of compound **1** (A) and compound **10** (B) on locomotor activity in mice. Compound **1** (10, 20, and 40 mg/kg, *n* = 8, i.p.), compound **10** (20, 40, and 80 mg/kg, i.p., *n* = 6–8), or vehicle (*n* = 8) were administered and after 30 min, the mouse locomotor activity was recorded for 30 min. Bonferroni’s *post hoc* test revealed the decrease in animal locomotor activity after the administration of compound **1** at the dose of 20 and 40 mg/kg (*p* < 0.05).

In the case of compound **1** (10, 20, and 40 mg/kg), one-way ANOVA revealed statistically significant changes in spontaneous locomotor activity in mice after a single injection of compound **1** (*F*(3, 28) = 5.549, *p* = 0.0041). More specifically, Bonferroni’s *post hoc* test indicates that 20 and 40 mg/kg compound **1** induces statistically significant decrease in the locomotor activity of tested animals (*p* < 0.05, Figure 8(A)).

However, one-way ANOVA revealed that compound **10** (20, 40, and 80 mg/kg) did not induce statistically significant changes in the tested doses (*F*(3, 24) = 1.968, *p* = 0.1456) as presented in Figure 8(B).

#### Effects of compounds 1 and 10 on amphetamine-induced hyperactivity in mice

In order to evaluate whether dopaminergic transmission is involved in the observed locomotion effects, the impact of studied compounds has been assessed in amphetamine-induced hyperactivity test. Animals were first administered with ineffective doses of compounds **1** and **10** (doses that did not impair animals’ mobility, 10 and 40 mg/kg, respectively) and second (30 min later) were treated with amphetamine. Animals’ locomotion was recorded for 30 min immediately after amphetamine injection. Amelioration of amphetamine-induced hyperactivity is interpreted as involvement of dopaminergic transmission in the locomotor effects of studied compounds.

**Figure 9.**
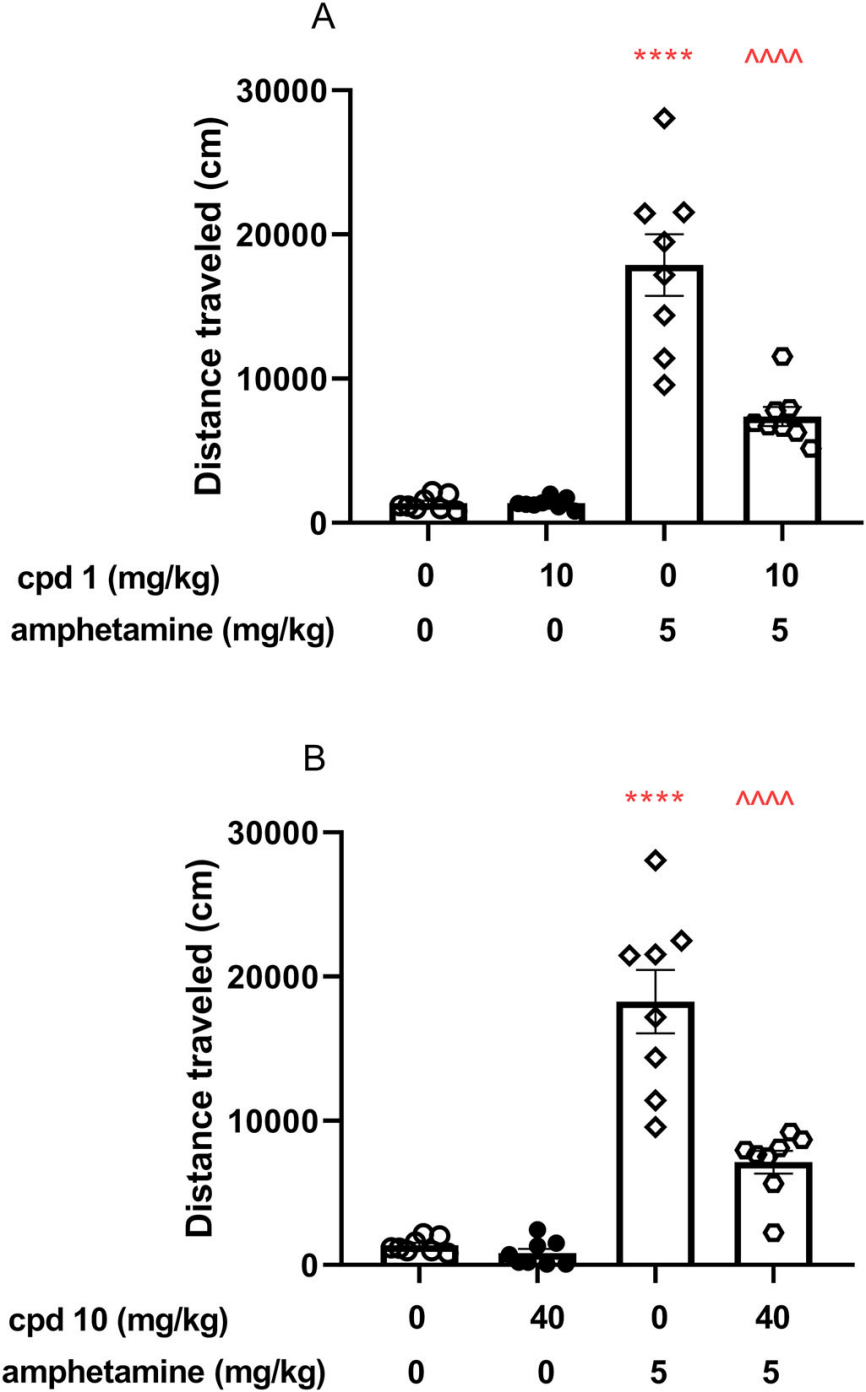
Influence of compounds **1** and **10** on hyperactivity induced by amphetamine in mice. Appropriate treatment groups received 10 mg/kg compound **1** (*n* = 8, i.p.), 40 mg/kg compound **10** (*n* = 8), 5 mg/kg amphetamine (*n* = 8, s.c.), 10 mg/kg compound **1** co-administered with amphetamine (*n* = 8), 40 mg/kg compound **10** co-injected with 5 mg/kg amphetamine (*n* = 8), and vehicle (*n* = 8) indicated as 0. Animals were injected with each compound or vehicle and received the second injection with vehicle or amphetamine 30 min after the first injection. The spontaneous locomotor activity was recorded for 30 min after the second injection. Data are presented as the mean ± SEM of the distance travelled (cm) by the mouse. Bonferroni’s *post hoc* test results: ****p < 0.0001 amphetamine vs. the control group and ^^^^*p* < 0.0001 for compound **1** or compound **10** co-administered with amphetamine vs. amphetamine-treated group (presented in panels A and B, respectively).

Two-way ANOVA analysis revealed that co-injection of 10 mg/kg compound **1** and 40 mg/kg compound **10** with 5 mg/kg amphetamine induces statistically significant pre-treatment effect (compound **1**: *F*(1, 28) = 21.79, *p* < 0.0001; compound **10**: *F*(1, 28) = 24.04, *p* < 0.0001), treatment effect (compound **1**: *F*(1, 28) = 100.2, *p* < 0.0001; compound **10**: *F*(1, 28) = 98.11, *p* < 0.0001), and statistically significant interaction effect between treatment and pre-treatment (compound **1**: *F*(1, 28) = 21.82, *p* < 0.0001; compound **10**: *F*(1, 28) = 19.58, *p* = 0.0001). In addition, Bonferroni’s *post hoc* test indicated increased locomotor activity after amphetamine treatment when compared to the vehicle group (*p* < 0.0001, Figure 9(A,B)) and statistically decreased the mouse activity after co-injection of both studied compounds (*p* < 0.0001 when compared to compound **1** or compound **10** co-administered with amphetamine vs. amphetamine alone) as presented in Figure 9(A,B), respectively.

The assessment of *in vivo* antipsychotic properties of novel drug candidates is most commonly performed with the use of the amphetamine model of schizophrenia^30^. Amphetamine causes locomotor hyperactivity in rodents by increasing dopaminergic activity in the mesolimbic circuit^31^^,^^32^. Antipsychotics that show antagonism towards dopamine D_2_ receptor reverse this effect. Both studied compounds **1** and **10**, which were shown to be D_2_ receptor antagonists, significantly reduced hyperactivity induced by amphetamine in mice at the studied doses, therefore, displaying antipsychotic-like properties mediated by this receptor.

#### Influence of compound 1 and compound 10 on mice’ motor coordination

In order to make sure that the studied compounds do not affect animal’s motor coordination, this parameter has been assessed in the rota-rod test for 3 min, 60 min after the animals received the treatment.

Rota-rod test indicated that neither compound **1** (10, 20, and 40 mg/kg) nor compound **10** (20, 40, and 80 mg/kg) influenced the mouse’s motor coordination. More specifically, one-way ANOVA indicates the lack of statistically significant difference (compound **1**: *F*(3, 26) = 0.5457, *p* = 0.6554; compound **10**: *F*(3, 25) = 2.42, *p* = 0.0898).

#### Effects of compound 1 and compound 10 on mice’ memory performance

**Figure 10.**
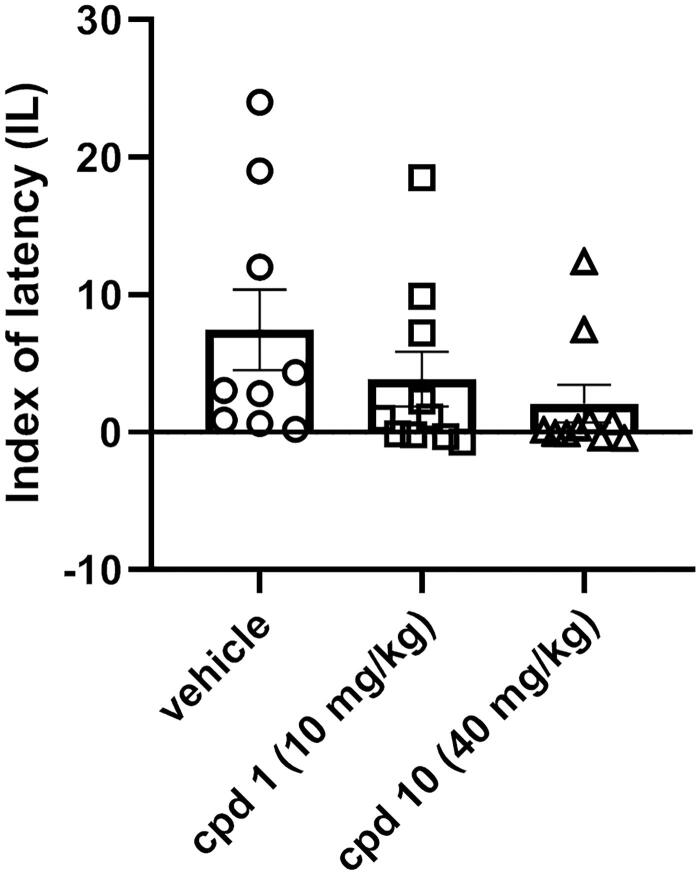
Influence of compounds **1** and **10** on memory acquisition in mice. Appropriate groups were injected with 10 mg/kg compound **1** (*n* = 10, i.p.), 40 mg/kg compound **10** (*n* = 10, i.p.), and vehicle (*n* = 9, i.p.) 60 min before the PA test. There is a lack of statistically significant difference between groups (one-way ANOVA, *p* > 0.05).

The influence of studied compounds **1** and **10** on memory processes was evaluated using the PA test after acute injection. This test is commonly used for the assessment of memory performance and is based on the association between acute negative experience (electric foot shock) and the environment in which this stressor is given. Animals were treated with compound **1** (10 mg/kg), compound **10** (40 mg/kg) or vehicle and after 60 min were tested in the PA test. After the introduction of the electric shock in the dark compartment on day 1 (60 min after treatment), the lack of the avoidance of this compartment on day 2 is interpreted as a deterioration of memory performance. One-way ANOVA revealed that there is a lack of statistically significant difference between control-treated group and tested compounds (*F*(2, 26) = 1.590, *p* = 0.2232) as presented in Figure 10.

Cognitive impairments are frequently observed in the course of schizophrenia. These types of symptoms are often resistant to treatment, and moreover, the use of some antipsychotics may even lead to their occurrence or exacerbation, which is reflected in the impairment of PA performance^33^. The effect of antipsychotics on memory processes is suggested to be mediated through the serotonin 5-HT_2A_ receptor. Activation of this receptor leads to an improvement in memory acquisition, while blocking it reverses this effect^34^. Compounds **1** and **10** at the studied doses show no statistically significant effect on PA performance, which may be considered beneficial, given that many antipsychotics have a negative effect on cognitive functioning. Compared to studied compounds **1** and **10**, compound D2AAK3 shows improvement of PA performance^12^. This may be explained by the fact that D2AAK3 displays a lower affinity for the 5-HT_2A_ receptor than both of its derivatives, **1** and **10**.

#### Effects of compound 1 and compound 10 on mice’ anxiety level

**Figure 11.**
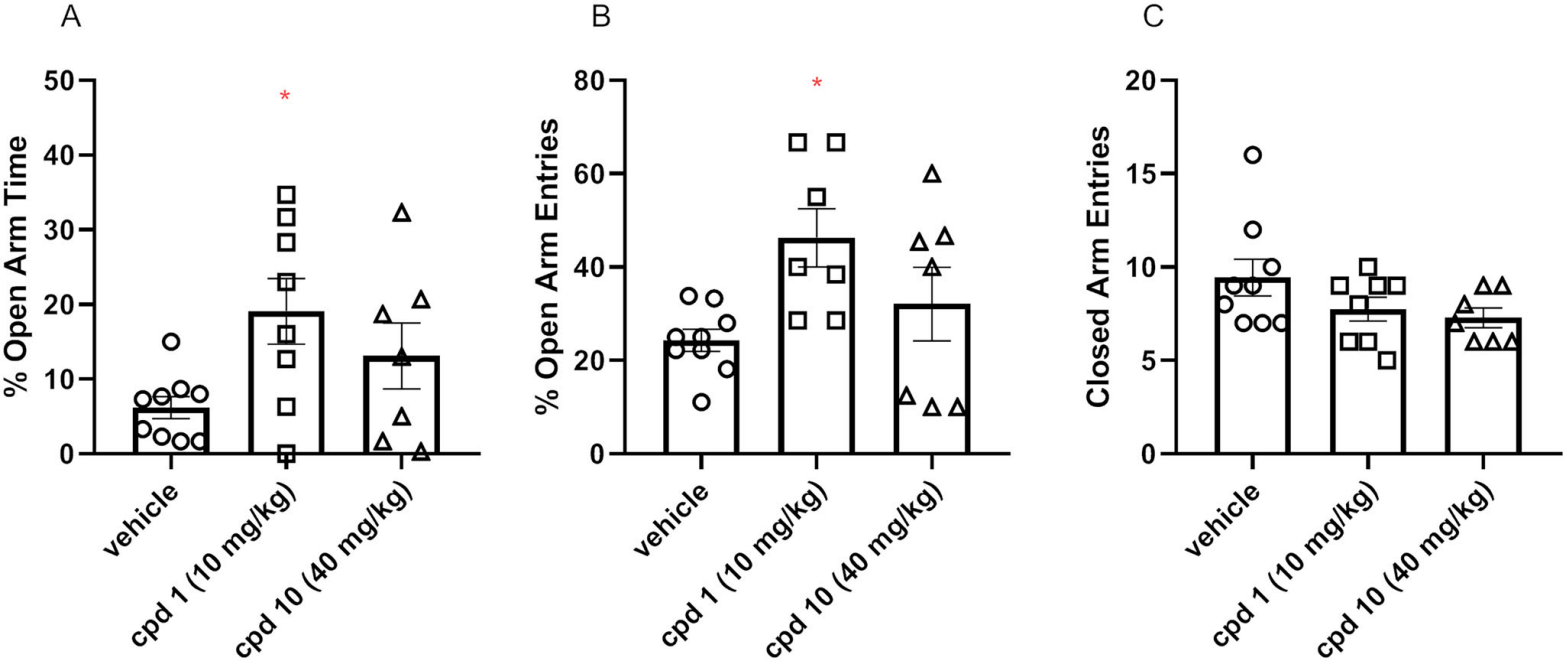
Acute effects of compounds **1** and **10** on anxiety-like behaviour in mice. Appropriate groups were administered with 10 mg/kg compound **1** (*n* = 8, i.p.), 40 mg/kg compound **10** (*n* = 7, i.p.), and vehicle (*n* = 9) 60 min before the EPM test. The values represent the mean ± SEM of percentage of open arms time (A), percentage of open arm entries (B), and closed arm entries (C).

EPM test was used to assess both compound **1** and compound **10** acute effects on anxiety-like behaviour in mice. Animals were injected with compound **1** (10 mg/kg), compound **10** (40 mg/kg), or vehicle and, after 60 min, were subjected to the EPM test. An increase in the time spent in the open arms and/or an increased frequency of entries to the open arms are the variables interpreted as an anxiolytic effect in the EPM test. One-way ANOVA revealed statistically significant influence on the percentage of the open arms time (*F*(2, 21) = 3.624, *p* = 0.0444), the percentage of open arms entries (*F*(2, 20) = 4.130, *p* = 0.0315) as presented in Figure 11. More specifically, Bonferroni’s *post hoc* test indicated that compound **1** elicited the increase in both percentages of open arm time (Figure 11(A)) and open arm entries (Figure 11(B)) when compared to the control respective group. Moreover, the number of entries to closed arms was measured (Figure 11(C)) and there was no difference between tested groups (*F*(2, 21) = 2.132, *p* = 0.1436).

As suggested, influencing serotonin neurotransmission via activation of the 5-HT_1A_ receptor can be involved in the regulation of anxiety processes^35^. Depending on the location of this receptor, this effect may have a different character. An anxiolytic effect may result from activating presynaptic 5-HT_1A_ receptors in structures such as dorsal or median raphe nuclei, while stimulating postsynaptic 5-HT_1A_ receptors located in the hippocampus or amygdala may constitute the mechanism underlying an anxiogenic effect^36^^,^^37^. Despite the similar affinity of compounds **1** and **10** to the 5-HT_1A_ receptor, only compound **1** produces anxiolytic effect in the EPM test, while compound **10** shows no statistically significant effect on the EPM performance. Furthermore, compound D2AAK3, which shows higher 5-HT_1A_ affinity than **1** and **10**, displays no impact on anxiety 60 min after administration in the EPM test^12^. It may be partially explained by the use of different doses of compounds **1**, **10**, and D2AAK3, and the complex effect of 5-HT_1A_ receptor agonists on anxiety processes; however, further investigation is required to explain in detail the different effects of D2AAK3 and its derivatives on 5-HT_1A_ receptor-mediated anxiety processes.

## Conclusions

The aim of our work was to develop novel molecules, similar to atypical neuroleptics in terms of mechanism of action, with antipsychotic potential. For this purpose, attempts were made to optimise compound D2AAK3, which was identified in virtual screening in previous studies as one of the candidates for the lead structure with antipsychotic activity. This compound is a multi-target ligand of aminergic GPCRs, which play an important role in schizophrenia, mainly dopamine D_2_ and serotonin 5-HT_1A_ and 5-HT_2A_ receptors. In the optimisation process of D2AAK3, 16 new derivatives were designed and synthesised by modifying the aromatic ring in the arylpiperazine system. As a result of *in vitro* pharmacological evaluation, the affinities of the obtained compounds for D_2_, 5-HT_1A_, and 5-HT_2A_ receptors were determined. Moreover, molecular docking was performed for the obtained compounds, which allowed us to determine their mode of binding to the receptors of interest. One of the goals of optimising D2AAK3 was to fine tune the activity at D_2_ and 5-HT_2A_ receptors in order to obtain molecules with a more atypical profile, i.e. having higher affinity for 5-HT_2A_ than for D_2_ receptor. Complementary, functional tests were performed for selected compounds **1**, **10**, and **11**, which revealed that the tested compounds are D_2_ and 5-HT_2A_ receptor antagonists and 5-HT_1A_ receptor agonists. Such receptor profile is beneficial in the context of the search for new drugs for the treatment of schizophrenia. In addition, receptor profiles of these compounds were extended by determining affinities for D_1_, D_3_, and 5-HT_7_ receptors. Selectivity studies of compounds **1**, **10**, and **11** over histamine H_1_ and muscarinic M_1_ receptors, which are associated with the occurrence of side effects, were performed and showed that these compounds have low affinity for the H_1_ receptor and no affinity for the M_1_ receptor, which is favourable in terms of the safety profile of potential drugs. Compounds **1** and **10** were selected for further *in vivo* evaluation, which revealed that these compounds exhibit predicted antipsychotic activity in the classic test of reducing amphetamine-induced hyperactivity in mice. Moreover, the tested compounds do not affect memory performance in PA test, indicating no effect on memory processes after acute administration. In turn, in the EPM test, compound **1** shows an anxiolytic effect. Finally, crystallographic studies were performed for compound **1**, revealing its stable conformation in the solid state. Future optimisation campaigns of the lead candidate D2AAK3 should focus especially on further improvement of its receptor profile. The presence of a fluorine substituent in the *para* position of the phenyl ring is most favourable for the activity on the 5-HT_2A_ receptor, as well as for increasing 5-HT_2A_/D_2_ affinity ratio. However, it also decreases the affinity towards 5-HT_1A_ receptor. On the other hand, fluorine in the *meta* position or unsubstituted phenyl translate into compounds with a more balanced receptor profile. Therefore, it seems reasonable to further investigate the impact of the different fluorine substitutions in the arylpiperazine moiety with the simultaneous modification of other fragments of the molecule, in particular the indazole part. Improvement in *in vivo* parameters, in particular PA performance, should also be considered. It may be mediated through 5-HT_7_ receptor, therefore, next steps of optimisation should be aimed at increasing affinity also for this target.

## Materials and methods

### Chemistry

All reagents used for the chemical synthesis were obtained from commercial suppliers or were in-house available, and were sufficiently pure, thus no prior purification was needed. NMR spectra were recorded on a Bruker AVANCE III 600 MHz or Bruker AVANCE III HD 500 MHz (Billerica, MA) apparatus with the use of DMSO-*d*_6_ or CDCl_3_ as deuterated solvents. Chemical shifts are presented in parts per million (*δ*), and are referenced to the residual proton signal in the respective solvent. Coupling constants (*J*) are reported in Hertz (Hz). High-resolution mass spectra (HRMS) were acquired on a Bruker microTOF-Q II mass spectrometer, using the positive mode of electrospray ionisation and a time-of-flight (TOF) mass analyser. Acetonitrile was used as a solvent. Collected spectral data were processed with the use of MestReNova v.14.0.0 and Compass Data Analysis software. For the purity determination of compounds **1** and **10**, samples were analysed using an Agilent 1290 Infinity HPLC system equipped with diode array detector (Agilent Technologies, Santa Clara, CA). Zorbax Extend C18 RRHD 2.1 × 100 mm, 1.8 µm (Agilent Technologies, Santa Clara, CA) was used. Mobile phase (A) 0.1% formic acid, (B) 0.1% formic acid in acetonitrile, linear gradient from 5 to 95% B in 30 min, flow rate 0.4 mL/min, and injection volume 1 µL. UV spectra were acquired from 190 to 400 nm. Samples were dissolved in methanol (1 mg/mL). Compound purities were calculated at 254 nm. Data processing was carried out using Mass Hunter Qualitative B0.07 software (Agilent Technologies, Santa Clara, CA). NMR and HRMS spectra for the synthesised compounds, as well as HPLC-DAD chromatograms of compounds **1** and **10** can be found in Supplementary Information.

#### Synthesis of compound 1a

Phthalimide (0.2 mol), 1,3-dibromopropane (0.6 mol), anhydrous potassium carbonate (0.2 mol), and butan-2-one (250 mL) were placed in a round bottom flask, and the mixture was heated to the boiling point of the solvent and stirred for 10 h. After cooling to ambient temperature, the solids were filtered off and washed with butan-2-one. The filtrates were combined and the solvent was removed on a rotavap. The obtained oil was crystallised from *n*-heptane, giving the desired product as a white solid.

#### *General procedure for the synthesis of compounds* 1b–16b

A round bottom flask was charged with 2-(3-bromopropyl)isoindoline-1,3-dione (3 mmol), obtained in the previous step, the corresponding piperazine derivative (3 mmol), anhydrous potassium carbonate (6 mmol), and potassium iodide (catalytic amount). Acetonitrile (9 mL) was added to the flask and the mixture was stirred and refluxed for 5–22 h (monitored by TLC). Then, the reaction mixture was cooled down and the precipitates of inorganic salts were filtered off and washed with the solvent. After combining the filtrates, acetonitrile was removed under reduced pressure. The obtained solid product was used in a subsequent step without further purification.

#### *General procedure for the synthesis of compounds* 1c–16c

The solution of the appropriate intermediate compound (**1b**–**16b**) in ethanol 96% was placed in a round bottom flask and 1.3 equiv. of hydrazine monohydrate was added. The mixture was refluxed for 3 h and then cooled to ambient temperature. Water was added to the flask and ethanol was removed on rotary evaporator. Hydrochloric acid was added to the obtained water solution to pH = 1. The resulting precipitate was filtered off and the filtrate was alkalised to pH = 10 with sodium hydroxide solution. The mixture was then extracted with three portions of methylene chloride. The organic layers were combined and dried over anhydrous magnesium sulphate. Subsequent evaporation of the solvent gave the product that was used in the final step without further purification.

#### *General procedure for the synthesis of compounds* 1–16

A reaction vial was charged with 1*H*-indazole-3-carboxylic acid (1 mmol) and 1 equiv. of 1,1′-carbonyldiimidazole (CDI). Dimethylformamide (3 mL) was added to the reaction and it was heated at 60 °C and stirred for 2 h. Next, the solution of the corresponding intermediate **1c**–**16c** (1 equiv.) in dimethylformamide (3 mL) was added to the vial and heated at 60 °C for the next 2.5 h. In the case of precipitate formation upon cooling down after reaction completion, it was filtered off and purified by washing with dimethylformamide and water. Otherwise, the solvent was distilled off, methylene chloride was added to the obtained residue and the resulting solution was washed with water, 1% sodium hydroxide solution, and saturated sodium chloride solution. The organic layer was then dried over anhydrous magnesium sulphate and the solvent was removed under reduced pressure. The crude product was purified by crystallisation from acetonitrile or washing with acetonitrile, or by dry column vacuum chromatography on silica gel with MeOH/DCM used as an eluent, in 2–6% gradient.

##### N-(3-(4-phenylpiperazin-1-yl)propyl)-1H-indazole-3-carboxamide (1)

Compound purified by crystallisation from ACN: white solid. Overall yield: 50%. ^1^H NMR (600 MHz, DMSO) *δ* 13.52 (s, 1H), 8.53 (t, *J* = 5.8 Hz, 1H), 8.19 (d, *J* = 8.2 Hz, 1H), 7.60 (d, *J* = 8.4 Hz, 1H), 7.44–7.37 (m, 1H), 7.26–7.18 (m, 3H), 6.93 (d, *J* = 7.8 Hz, 2H), 6.77 (t, *J* = 7.2 Hz, 1H), 3.41–3.37 (m, 2H), 3.19–3.12 (m, 4H), 2.55–2.52 (m, 4H), 2.43 (t, *J* = 6.9 Hz, 2H), and 1.76 (p, *J* = 6.9 Hz, 2H). ^13^C NMR (151 MHz, DMSO) *δ* 162.66, 151.51, 141.58, 138.90, 129.38, 126.89, 122.38, 122.10, 121.92, 119.13, 115.77, 111.09, 56.62, 53.30, 48.62, 37.87, and 26.69. HRMS (ESI) *m/z* [M + H]^+^ calculated for C_21_H_25_N_5_O: 364.2132, found: 364.2131.

##### N-(3-(4-(2-methoxyphenyl)piperazin-1-yl)propyl)-1H-indazole-3-carboxamide (2)

Compound purified by DCVC (MeOH/DCM, 2–6% gradient): white solid. Overall yield: 52%. ^1^H NMR (500 MHz, DMSO) *δ* 13.53 (s, 1H), 8.52 (t, *J* = 5.8 Hz, 1H), 8.19 (dt, *J* = 8.2, 1.1 Hz, 1H), 7.60 (dt, *J* = 8.4, 0.9 Hz, 1H), 7.40 (ddd, *J* = 8.3, 6.9, 1.1 Hz, 1H), 7.23 (ddd, *J* = 7.9, 6.9, 0.9 Hz, 1H), 6.95–6.89 (m, 2H), 6.89–6.84 (m, 2H), 3.76 (s, 3H), 3.38 (q, *J* = 6.7 Hz, 2H), 2.98 (s, 4H), 2.58–2.50 (m, 4H), 2.42 (t, *J* = 6.9 Hz, 2H), and 1.75 (p, *J* = 6.9 Hz, 2H). ^13^C NMR (126 MHz, DMSO) *δ* 162.18, 151.96, 141.29, 141.10, 138.43, 126.41, 122.26, 121.90, 121.64, 121.45, 120.86, 117.90, 111.87, 110.62, 56.22, 55.30, 53.11, 50.03, 37.41, and 26.21. HRMS (ESI) *m/z* [M + H]^+^ calculated for C_22_H_27_N_5_O_2_: 394.2238, found: 394.2227.

##### N-(3-(4-(3-methoxyphenyl)piperazin-1-yl)propyl)-1H-indazole-3-carboxamide (3)

Compound purified by washing with ACN: white solid. Overall yield: 43%. ^1^H NMR (600 MHz, DMSO) *δ* 13.51 (s, 1H), 8.53 (t, *J* = 5.8 Hz, 1H), 8.20 (dd, *J* = 8.2, 1.1 Hz, 1H), 7.60 (d, *J* = 8.4 Hz, 1H), 7.41 (ddd, *J* = 8.3, 6.8, 1.2 Hz, 1H), 7.26–7.21 (m, 1H), 7.10 (t, *J* = 8.2 Hz, 1H), 6.51 (dd, *J* = 8.2, 2.3 Hz, 1H), 6.44 (t, *J* = 2.4 Hz, 1H), 6.36 (dd, *J* = 8.1, 2.3 Hz, 1H), 3.71 (s, 3H), 3.40–3.39 (m, 2H), 3.15 (t, *J* = 5.1 Hz, 4H), 2.51–2.50 (m, 4H), 2.42 (t, *J* = 6.9 Hz, 2H), and 1.76 (p, *J* = 6.9 Hz, 2H). ^13^C NMR (151 MHz, DMSO) *δ* 162.67, 160.67, 152.86, 141.58, 138.90, 130.05, 126.89, 122.39, 122.11, 111.09, 108.44, 104.47, 101.83, 56.62, 55.31, 53.26, 48.57, 37.88, and 26.67. HRMS (ESI) *m/z* [M + H]^+^ calculated for C_22_H_27_N_5_O_2_: 394.2238, found: 394.2239.

##### N-(3-(4-(4-methoxyphenyl)piperazin-1-yl)propyl)-1H-indazole-3-carboxamide (4)

Compound purified by washing with ACN: white solid. Overall yield: 30%. ^1^H NMR (600 MHz, DMSO) *δ* 13.49 (s, 1H), 8.51 (t, *J* = 5.8 Hz, 1H), 8.19 (dt, *J* = 8.2, 1.1 Hz, 1H), 7.62–7.58 (m, 1H), 7.41 (ddd, *J* = 8.2, 6.8, 1.1 Hz, 1H), 7.24 (ddd, *J* = 7.9, 6.8, 0.9 Hz, 1H), 6.91–6.85 (m, 2H), 6.84–6.78 (m, 2H), 3.68 (s, 3H), 3.41–3.39 (m, 2H), 3.03 (t, *J* = 4.9 Hz, 4H), 2.54–2.51 (m, 4H), 2.42 (t, *J* = 6.9 Hz, 2H), and 1.75 (p, *J* = 6.9 Hz, 2H). ^13^C NMR (151 MHz, DMSO) *δ* 162.68, 153.28, 145.93, 141.58, 138.90, 126.89, 122.39, 122.10, 121.92, 117.74, 114.72, 111.09, 56.60, 55.65, 53.40, 50.02, 37.86, and 26.71. HRMS (ESI) *m/z* [M + H]^+^ calculated for C_22_H_27_N_5_O_2_: 394.2238, found: 394.2238.

##### N-(3-(4-(pyridin-2-yl)piperazin-1-yl)propyl)-1H-indazole-3-carboxamide (5)

Compound purified by DCVC (MeOH/DCM, 2–6% gradient): white solid. Overall yield: 44%. ^1^H NMR (500 MHz, CDCl_3_) *δ* 11.89 (s, 1H), 8.49 (t, *J* = 5.4 Hz, 1H), 8.42 (dt, *J* = 8.2, 1.0 Hz, 1H), 8.16 (ddd, *J* = 5.0, 2.0, 0.9 Hz, 1H), 7.45–7.39 (m, 2H), 7.39–7.33 (m, 1H), 7.26–7.22 (m, 1H), 6.61–6.55 (m, 2H), 3.66–3.59 (m, 6H), 2.60–2.53 (m, 6H), and 1.83 (p, *J* = 6.2 Hz, 2H). ^13^C NMR (126 MHz, CDCl_3_) *δ* 162.98, 159.31, 147.63, 141.46, 139.49, 137.63, 126.94, 122.66, 122.48, 122.08, 113.15, 109.84, 107.29, 57.86, 53.11, 45.11, 39.12, and 25.37. HRMS (ESI) *m/z* [M + H]^+^ calculated for C_20_H_24_N_6_O: 365.2084, found: 365.2068.

##### N-(3-(4-(pyridin-3-yl)piperazin-1-yl)propyl)-1H-indazole-3-carboxamide (6)

Compound purified by washing with ACN: white solid. Overall yield: 5%. ^1^H NMR (600 MHz, DMSO) *δ* 13.52 (s, 1H), 8.55 (t, *J* = 5.7 Hz, 1H), 8.30 (d, *J* = 2.9 Hz, 1H), 8.19 (d, *J* = 8.1 Hz, 1H), 8.01–7.97 (m, 1H), 7.60 (d, *J* = 8.4 Hz, 1H), 7.45–7.38 (m, 1H), 7.33–7.28 (m, 1H), 7.26–7.18 (m, 2H), 3.41–3.39 (m, 2H), 3.22 (t, *J* = 5.0 Hz, 4H), 2.53 (t, *J* = 5.0 Hz, 4H), 2.43 (t, *J* = 6.8 Hz, 2H), and 1.76 (p, *J* = 6.9 Hz, 2H). ^13^C NMR (151 MHz, DMSO) *δ* 162.67, 147.13, 141.58, 140.00, 138.90, 138.02, 126.90, 123.96, 122.39, 122.10, 122.07, 121.91, 111.09, 56.61, 53.02, 47.93, 37.89, and 26.61. HRMS (ESI) *m/z* [M + H]^+^ calculated for C_20_H_24_N_6_O: 365.2084, found: 365.2082.

##### N-(3-(4-(pyridin-4-yl)piperazin-1-yl)propyl)-1H-indazole-3-carboxamide (7)

Compound filtered from the reaction mixture and washed with DMF and water: white solid. Overall yield: 4%. ^1^H NMR (600 MHz, DMSO) *δ* 13.53 (s, 1H), 8.57 (t, *J* = 5.7 Hz, 1H), 8.18 (d, *J* = 8.1 Hz, 1H), 8.15 (d, *J* = 6.4 Hz, 2H), 7.59 (d, *J* = 8.4 Hz, 1H), 7.41 (t, *J* = 7.6 Hz, 1H), 7.24 (t, *J* = 7.5 Hz, 1H), 6.82 (d, *J* = 6.5 Hz, 2H), 3.41–3.38 (m, 6H), 2.55–2.52 (m, 4H), 2.43 (t, *J* = 6.9 Hz, 2H), and 1.76 (p, *J* = 6.7 Hz, 2H). ^13^C NMR (151 MHz, DMSO) *δ* 162.64, 154.96, 150.27, 141.57, 138.88, 126.90, 122.39, 122.09, 121.90, 111.09, 108.78, 56.59, 52.80, 45.87, 37.91, and 26.54. HRMS (ESI) *m/z* [M + H]^+^ calculated for C_20_H_24_N_6_O: 365.2084, found: 365.2082.

##### N-(3-(4-(pyrimidin-2-yl)piperazin-1-yl)propyl)-1H-indazole-3-carboxamide (8)

Compound purified by washing with ACN: white solid. Overall yield: 18%. ^1^H NMR (600 MHz, DMSO) *δ* 13.53 (s, 1H), 8.62 (t, *J* = 5.7 Hz, 1H), 8.35 (d, *J* = 4.6 Hz, 2H), 8.21–8.17 (m, 1H), 7.61–7.57 (m, 1H), 7.40 (ddd, *J* = 8.2, 6.8, 1.2 Hz, 1H), 7.26–7.21 (m, 1H), 6.61 (t, *J* = 4.7 Hz, 1H), 3.79–3.75 (m, 4H), 3.41–3.39 (m, 2H), 2.47–2.40 (m, 6H), and 1.76 (p, *J* = 6.8 Hz, 2H). ^13^C NMR (151 MHz, DMSO) *δ* 162.64, 161.66, 158.36, 141.57, 138.90, 126.89, 122.39, 122.09, 121.90, 111.08, 110.46, 56.81, 53.14, 43.75, 38.04, and 26.46. HRMS (ESI) *m/z* [M + H]^+^ calculated for C_19_H_23_N_7_O: 366.2037, found: 366.2036.

##### N-(3-(4-(2-fluorophenyl)piperazin-1-yl)propyl)-1H-indazole-3-carboxamide (9)

Compound purified by washing with ACN: white solid. Overall yield: 23%. ^1^H NMR (600 MHz, DMSO) *δ* 13.52 (s, 1H), 8.53 (t, *J* = 5.8 Hz, 1H), 8.18 (d, *J* = 8.1 Hz, 1H), 7.60 (d, *J* = 8.4 Hz, 1H), 7.41 (t, *J* = 7.6 Hz, 1H), 7.23 (t, *J* = 7.5 Hz, 1H), 7.14–7.08 (m, 2H), 7.06–7.00 (m, 1H), 6.99–6.93 (m, 1H), 3.40–3.38 (m, 2H), 3.10–2.98 (m, 4H), 2.58–2.52 (m, 4H), 2.44 (t, *J* = 6.9 Hz, 2H), and 1.75 (p, *J* = 6.9 Hz, 2H). ^13^C NMR (151 MHz, DMSO) *δ* 162.09, 154.84 (d, *J* = 244.2 Hz), 141.01, 139.81 (d, *J* = 8.3 Hz), 138.33, 126.32, 124.73 (d, *J* = 3.4 Hz), 122.04 (d, *J* = 8.0 Hz), 121.81, 121.53, 121.34, 119.07 (d, *J* = 3.1 Hz), 115.77 (d, *J* = 20.5 Hz), 110.52, 56.01, 52.76, 49.98 (d, *J* = 3.2 Hz), 37.30, and 26.07. HRMS (ESI) *m/z* [M + H]^+^ calculated for C_21_H_24_FN_5_O: 382.2038, found: 382.2038.

##### N-(3-(4-(3-fluorophenyl)piperazin-1-yl)propyl)-1H-indazole-3-carboxamide (10)

Compound purified by washing with ACN: white solid. Overall yield: 31%. ^1^H NMR (600 MHz, DMSO) *δ* 13.52 (s, 1H), 8.54 (t, *J* = 5.8 Hz, 1H), 8.18 (dt, *J* = 8.2, 1.1 Hz, 1H), 7.60 (dt, *J* = 8.4, 0.9 Hz, 1H), 7.41 (ddd, *J* = 8.3, 6.8, 1.1 Hz, 1H), 7.26–7.18 (m, 2H), 6.77–6.70 (m, 2H), 6.56–6.50 (m, 1H), 3.40–3.37 (m, 2H), 3.22–3.18 (m, 4H), 2.53–2.51 (m, 4H), 2.43 (t, *J* = 6.9 Hz, 2H), and 1.76 (p, *J* = 6.9 Hz, 2H). ^13^C NMR (151 MHz, DMSO) *δ* 163.78 (d, *J* = 240.2 Hz), 162.65, 153.26 (d, *J* = 10.2 Hz), 141.58, 138.89, 130.75 (d, *J* = 10.3 Hz), 126.89, 122.38, 122.10, 121.91, 111.22 (d, *J* = 2.0 Hz), 111.09, 104.94 (d, *J* = 21.2 Hz), 102.08 (d, *J* = 25.2 Hz), 56.58, 53.08, 48.08, 37.88, and 26.64. HRMS (ESI) *m/z* [M + H]^+^ calculated for C_21_H_24_FN_5_O: 382.2038, found: 382.2036.

##### N-(3-(4-(4-fluorophenyl)piperazin-1-yl)propyl)-1H-indazole-3-carboxamide (11)

Compound purified by washing with ACN: white solid. Overall yield: 14%. ^1^H NMR (600 MHz, DMSO) *δ* 13.52 (s, 1H), 8.53 (t, *J* = 5.8 Hz, 1H), 8.18 (dt, *J* = 8.2, 1.1 Hz, 1H), 7.60 (dt, *J* = 8.4, 1.0 Hz, 1H), 7.41 (ddd, *J* = 8.3, 6.8, 1.1 Hz, 1H), 7.23 (ddd, *J* = 8.0, 6.8, 0.9 Hz, 1H), 7.06–7.01 (m, 2H), 6.96–6.92 (m, 2H), 3.40–3.38 (m, 2H), 3.12–3.07 (m, 4H), 2.54–2.51 (m, 4H), 2.42 (t, *J* = 6.9 Hz, 2H), and 1.75 (p, *J* = 6.9 Hz, 2H). ^13^C NMR (151 MHz, DMSO) *δ* 162.09, 155.82 (d, *J* = 235.4 Hz), 147.85, 141.00, 138.32, 126.32, 121.82, 121.52, 121.34, 116.90 (d, *J* = 7.5 Hz), 115.12 (d, *J* = 21.7 Hz), 110.52, 55.99, 52.69, 48.83, 37.28, and 26.11. HRMS (ESI) *m/z* [M + H]^+^ calculated for C_21_H_24_FN_5_O: 382.2038, found: 382.2038.

##### N-(3-(4-(2-(trifluoromethyl)phenyl)piperazin-1-yl)propyl)-1H-indazole-3-carboxamide (12)

Compound purified by washing with ACN: white solid. Overall yield: 53%. ^1^H NMR (600 MHz, DMSO) *δ* 13.70 (s, 1H), 8.56 (t, *J* = 5.7 Hz, 1H), 8.18 (d, *J* = 8.2 Hz, 1H), 7.69–7.64 (m, 2H), 7.62 (d, *J* = 8.4 Hz, 1H), 7.56 (d, *J* = 7.9 Hz, 1H), 7.43–7.39 (m, 1H), 7.33 (t, *J* = 7.6 Hz, 1H), 7.25–7.21 (m, 1H), 3.41–3.38 (m, 2H), 2.92–2.86 (m, 4H), 2.58–2.51 (m, 4H), 2.45 (t, *J* = 6.8 Hz, 2H), and 1.75 (p, *J* = 6.8 Hz, 2H). ^13^C NMR (151 MHz, DMSO) *δ* 162.68, 152.93, 141.59, 138.86, 134.14, 127.35 (q, *J* = 5.7 Hz), 126.84, 126.02 (q, *J* = 27.9 Hz), 125.51, 124.96, 124.48 (q, *J* = 274.0 Hz), 122.35, 122.09, 121.91, 111.16, 56.66, 53.68, 53.61, 37.93, and 26.59. HRMS (ESI) *m/z* [M + H]^+^ calculated for C_22_H_24_F_3_N_5_O: 432.2006, found: 432.2005.

##### N-(3-(4-(2-chlorophenyl)piperazin-1-yl)propyl)-1H-indazole-3-carboxamide (13)

Compound purified by washing with ACN: white solid. Overall yield: 33%. ^1^H NMR (600 MHz, DMSO) *δ* 13.57 (s, 1H), 8.54 (t, *J* = 5.8 Hz, 1H), 8.19 (d, *J* = 8.2 Hz, 1H), 7.61 (d, *J* = 8.4 Hz, 1H), 7.43–7.38 (m, 2H), 7.33–7.28 (m, 1H), 7.26–7.21 (m, 1H), 7.18–7.14 (m, 1H), 7.06–7.01 (m, 1H), 3.41–3.39 (m, 2H), 3.05–2.96 (m, 4H), 2.65–2.51 (m, 4H), 2.45 (t, *J* = 6.8 Hz, 2H), and 1.76 (p, *J* = 6.9 Hz, 2H). ^13^C NMR (151 MHz, DMSO) *δ* 162.67, 149.54, 141.60, 138.92, 130.77, 128.56, 128.06, 126.89, 124.24, 122.38, 122.10, 121.92, 121.32, 111.10, 56.60, 53.44, 51.30, 37.90, and 26.64. HRMS (ESI) *m/z* [M + H]^+^ calculated for C_21_H_24_ClN_5_O: 398.1742, found: 398.1742.

##### N-(3-(4-(2-(methylthio)phenyl)piperazin-1-yl)propyl)-1H-indazole-3-carboxamide (14)

Compound was pure after workup–no additional purification needed: white solid. Overall yield: 60%. ^1^H NMR (600 MHz, DMSO) *δ* 13.56 (s, 1H), 8.55 (t, *J* = 5.8 Hz, 1H), 8.22–8.17 (m, 1H), 7.63–7.59 (m, 1H), 7.41 (ddd, *J* = 8.2, 6.8, 1.1 Hz, 1H), 7.24 (ddd, *J* = 8.0, 6.8, 0.9 Hz, 1H), 7.15–7.06 (m, 4H), 3.41–3.39 (m, 2H), 2.99–2.85 (m, 4H), 2.61–2.52 (m, 2H), 2.44 (t, *J* = 6.9 Hz, 3H), 2.36 (s, 3H), and 1.75 (p, *J* = 6.9 Hz, 2H). ^13^C NMR (151 MHz, DMSO) *δ* 162.67, 149.63, 141.58, 138.93, 134.92, 126.90, 125.27, 124.70, 124.63, 122.39, 122.11, 121.93, 119.98, 111.10, 56.67, 53.68, 51.60, 37.90, 26.65, and 14.03. HRMS (ESI) *m/z* [M + H]^+^ calculated for C_22_H_27_N_5_OS: 410.2009, found: 410.2009.

##### N-(3-(4-(2-ethoxyphenyl)piperazin-1-yl)propyl)-1H-indazole-3-carboxamide (15)

Compound purified by washing with ACN: white solid. Overall yield: 48%. ^1^H NMR (600 MHz, DMSO) *δ* 13.52 (s, 1H), 8.52 (t, *J* = 5.7 Hz, 1H), 8.19 (dt, *J* = 8.2, 1.0 Hz, 1H), 7.60 (dt, *J* = 8.4, 1.0 Hz, 1H), 7.41 (ddd, *J* = 8.3, 6.8, 1.1 Hz, 1H), 7.24 (ddd, *J* = 7.9, 6.8, 0.9 Hz, 1H), 6.92–6.84 (m, 4H), 4.00 (q, *J* = 7.0 Hz, 2H), 3.40–3.38 (m, 2H), 3.01 (s, 4H), 2.60–2.51 (m, 4H), 2.43 (t, *J* = 6.9 Hz, 2H), 1.76 (p, *J* = 6.9 Hz, 2H), and 1.33 (t, *J* = 7.0 Hz, 3H). ^13^C NMR (151 MHz, DMSO) *δ* 162.67, 151.62, 142.02, 141.59, 138.92, 126.88, 122.57, 122.37, 122.11, 121.92, 121.45, 118.38, 113.77, 111.09, 63.77, 56.72, 53.61, 50.43, 37.91, 26.66, and 15.31. HRMS (ESI) *m/z* [M + H]^+^ calculated for C_23_H_29_N_5_O_2_: 408.2394, found: 408.2384.

##### N-(3-(4-cyclohexylpiperazin-1-yl)propyl)-1H-indazole-3-carboxamide (16)

Compound purified by washing with ACN: white solid. Overall yield: 23%. ^1^H NMR (600 MHz, DMSO) *δ* 13.49 (s, 1H), 8.48 (t, *J* = 5.8 Hz, 1H), 8.17 (d, *J* = 8.1 Hz, 1H), 7.60 (d, *J* = 8.4 Hz, 1H), 7.41 (t, *J* = 7.6 Hz, 1H), 7.23 (t, *J* = 7.5 Hz, 1H), 3.35–3.31 (m, 2H), 2.50–2.46 (m, 5H), 2.33 (t, *J* = 6.9 Hz, 4H), 2.20–2.13 (m, 1H), 1.78–1.65 (m, 6H), 1.59–1.53 (m, 1H), and 1.25–1.02 (m, 6H). ^13^C NMR (151 MHz, DMSO) *δ* 162.64, 141.57, 138.91, 126.88, 122.36, 122.09, 121.91, 111.09, 62.95, 56.66, 53.94, 48.85, 37.85, 28.90, 26.73, 26.42, and 25.75. HRMS (ESI) *m/z* [M + H]^+^ calculated for C_21_H_31_N_5_O: 370.2601, found: 370.2601.

### Receptor radioligand binding assays

Competition radioligand binding assays were performed on membranes isolated from cell lines stable expressing the cloned human receptors, which were either in-house or commercially available. CHO-K1 cell lines with stable expression of the cloned human dopamine D_1_^12^, D_2S_^38^ and D_3_^12^, serotonin 5-HT_2A_^39^, histamine H_1_^40^, and muscarinic M_1_ receptors, as well as a HEK293 cell line with stable expression of the cloned human 5-HT_1A_^41^ and 5-HT_7_^12^ receptors, were used. Studied compounds were tested either at a single concentration of 10 μM or in competition binding curves, which were typically constructed with six different concentrations of a given compound. As internal controls, the following reference compounds were included in the assays: haloperidol (D_1_, D_2_, D_3_), 5-carboxamidotryptamine (5-CT) (5-HT_1A_), risperidone (5-HT_2A_), clozapine (5-HT_7_), doxepin (H_1_), and ipratropium (M_1_). Assays (250 μL assay final volume) were carried out in 96-well polypropylene plates in duplicate. In short, suspension of appropriate membrane protein was incubated in assay buffer with addition of the corresponding radioligand, in the presence or absence of tested compound or reference compound. [^3^H]-SCH-23390 (0.7 nM; D_1_ receptor), [^3^H]-spiperone (0.6 nM for D_2_ receptor, 1 nM for D_3_ receptor), [^3^H]-8-OH-DPAT (1 nM; 5-HT_1A_ receptor), [^3^H]-Ketanserin (1.25 nM; 5-HT_2A_), [^3^H]-SB269970 (2 nM; 5-HT_7_ receptor), [^3^H]-pyrilamine (4 nM; H_1_ receptor), and [^3^H]-pirenzepine (7 nM; M_1_ receptor) were employed as radioligands. To assess non-specific binding, membrane protein and radioligand were incubated in the presence of 1 μM (+)-butaclamol (D_1_), 100 μM sulpiride (D_2_), 1 µM haloperidol (D_3_), 10 μM serotonin (5-HT_1A_), 1 μM methysergide (5-HT_2A_), 25 μM clozapine (5-HT_7_), 10 μM triprolidine (H_1_), and 200 μM pirenzepine (M_1_). After the incubation, the content of the test plates was transferred to 96-well plates with glass fibre filter GF/B or GF/C, previously pre-treated with polyethyleneimine (PEI), and rapidly filtered with the use of vacuum, followed by rapid washing with cold (4 °C) wash buffer. After that the filter plates were dried at 60 °C for 1 h. Then, 30 μL of liquid scintillation cocktail (UniverSol-ES, MP Biomedicals, Solon, OH) was added to each well of the filter plates and the radioactivity was measured in a MicroBeta2 microplate scintillation counter (PerkinElmer, Madrid, Spain). Table S2, which can be found in Supplementary Information, shows the detailed conditions of experimental protocols applied in radioligand binding assays for each receptor.

#### Radioligand binding data analysis

Competition binding curves were fitted to a one-site competition binding model (Fit Ki) with the use of Prism 7 software (GraphPad, San Diego, CA). log*K*_i_ (log of the equilibrium dissociation constant (*K*_i_)) values were obtained after constraining the concentration of radioligand employed in the assay and its dissociation constant (*K*_D_) as determined in saturation radioligand binding assays.

### Functional assays of cAMP signalling at D_2_ receptors

The assessment of the activity of selected compounds at D_2_ receptor was performed in cell-based functional assays of cAMP signalling, in the CHO-K1 cell line with stable expression of the cloned human D_2S_ receptor, which was also employed for the radioligand binding assays. For D_2_ receptor antagonism, test compounds were dispensed into an empty 96-well plate (Isoplate-96 Black Frame White Well, PerkinElmer España SL, Madrid, Spain) with the use of the dispensing noncontact Echo 550 acoustic liquid handler (LABCYTE Inc., San Jose, CA). Compounds were handled from frozen 10 mM stock solutions in 100% DMSO, from which serial 1000× solutions were prepared in 100% DMSO at the time of the assay, keeping vehicle concentration (0.1% DMSO) constant in the assay. Vials containing viable frozen cells were quickly thawed in a 37 °C water bath and cells were seeded into the assay plate in assay buffer (kit stimulation buffer containing 500 µM 3-isobutyl-1-methylxanthine (IBMX), which was directly added to the buffer as powder). After 5 min incubation at 37 °C, reference agonist dopamine (dopamine hydrochloride, Sigma-Aldrich, Merck Life Science S.L.U., Madrid, Spain) (1 µM final concentration, prepared from freshly made aqueous stock solution) was added to the corresponding wells. After 10 min incubation at 37 °C, 10 µM forskolin was added to the corresponding wells and incubation was continued for 5 min. After this time, cellular cAMP levels were quantified using the homogeneous time-resolved fluorescence (HTRF)-based cAMP Gs dynamic kit (Cisbio, Bioassays, Codolet, France) according to the manufacturer’s protocol. Haloperidol (10^−10^ to 10^−5^ M) was used as a control antagonist in these assays. Basal cAMP levels were determined in control wells in the absence of compound, agonist, and forskolin. Concentration–response curves (10^−10^ to 10^−4^ M) of dopamine were included in the individual experiments for internal EC_50_ calculation. Individual concentration–response curves of the compounds were fitted to the model of log(inhibitor) vs. response (three parameters) (Hill slope (*n*_H_) = 1; best fit in comparison to log(inhibitor) vs. response–variable slope (four parameters) model, *p* < 0.05, extra sum-of-squares *F* test) described by the equation *Y* = Bottom + (Top – Bottom)/(1 + 10^((*X* – LogIC50))) using Prism 7 software (GraphPad, San Diego, CA) and pIC_50_ (–logIC_50_) values were extracted from the fitting. *K*_b_ value (equilibrium dissociation constant of a competitive antagonist extracted from a functional assay) of the compounds was estimated according to the Leff–Dougall variant of the Cheng–Prusoff equation *K*_b_ = IC_50_/((2 + ([Ag]/[EC_50_])*^n^*)^1/^*^n^* – 1), where IC_50_ is the concentration of antagonist that inhibits agonist response by a 50%; [Ag] is the concentration of agonist employed in the assay, [EC_50_] is the agonist EC_50_ value in the assay, and *n* is the Hill slope of the concentration–response curve of the agonist^42^.

### Functional assays of cAMP signalling at 5-HT_1A_ receptors

The activity of selected compounds at 5-HT_1A_ receptor was investigated in cell-based functional assays of cAMP signalling in the HEK293 cell line stably expressing the cloned human 5-HT_1A_ receptor also employed for the radioligand binding assays. Twenty-four hours before the experiment, cells in culture were seeded in complete growth medium prepared with dialysed foetal bovine serum, in 96-well tissue culture plates (Greiner CELLSTAR^®^ white 96-well plates, Sigma-Aldrich, Merck Life Science S.L.U., Madrid, Spain). At the day of assay, plates were washed with assay buffer (kit stimulation buffer containing 500 µM IBMX) and incubated with test compounds or control agonist 5-carboxamidotryptamine (10^−12^ to 10^−5^ M) (5-carboxamidotryptamine maleate salt, Sigma-Aldrich, Merck Life Science S.L.U., Madrid, Spain) for 10 min at 37 °C. Test compounds were added to the assay plates from 5× working dilution intermediate plates, which were prepared by means of acoustic noncontact dispensing of the compounds, keeping vehicle concentration (0.1% DMSO) constant in the assay. After this time, 1 µM forskolin was added to the corresponding wells and incubation was continued for 5 min before cellular cAMP levels were quantified as described for functional assays of cAMP signalling at D_2_ receptors. Individual concentration–response curves were fitted to the model of log(agonist) vs. response (three parameters) (Hill slope (*n*_H_) = 1; best fit in comparison to log(agonist) vs. response – variable slope (four parameters) model, *p* < 0.05, extra sum-of-squares *F* test) described by the equation *Y* = Bottom + (Top – Bottom)/(1 + 10^((LogEC50 – *X*))) using Prism 7 software (GraphPad, San Diego, CA) and pEC_50_ (–logEC_50_) values were extracted from the fitting.

### Functional assays of inositol phosphate production at 5-HT_2A_ receptors

The activity of selected compounds at 5-HT_2A_ receptors was investigated in cell-based functional assays of IP production in the CHO-K1 cell line stably expressing the cloned human 5-HT_2A_ receptor also employed for the radioligand binding assays. Twenty-four hours before the experiment, cells in culture were seeded in complete growth medium prepared with dialysed foetal bovine serum, in half-area, white, 96-well tissue culture plates (Corning^®^, Fisher Scientific SL, Madrid, Spain). At the day of assay, plates were washed with assay buffer (kit stimulation buffer) and incubated with test compounds for 10 min at 37 °C. Test compounds were added to the assay plates from 7× working dilution intermediate plates that were prepared by means of acoustic noncontact dispensing of the compounds, keeping vehicle concentration (0.1% DMSO) constant in the assay. After this time, reference agonist serotonin (serotonin hydrochloride, Sigma-Aldrich, Merck Life Science S.L.U., Madrid, Spain) (1 µM final concentration, prepared from freshly made aqueous stock solution) was added to the corresponding wells and incubation was continued for 20 min at 37 °C. After this time, cellular IP levels were quantified by using the HTRF-based inositol monophosphate kit IP-One Gq kit (Cisbio, Bioassays, Codolet, France) following the manufacturer’s protocol. Risperidone (10^−12^ to 10^−7^ M) was used as a control antagonist in these assays. Basal IP levels were determined in control wells in the absence of compound and agonist. Concentration–response curves (10^−10^ to 10^−4^ M) of serotonin were included in the individual experiments for internal EC_50_ calculation. Individual concentration–response curves were fitted to the model of log(inhibitor) vs. response (three parameters) (Hill slope (*n*_H_) = 1; best fit in comparison to log(inhibitor) vs. response – variable slope (four parameters) model, *p* < 0.05, extra sum-of-squares *F* test) described by the equation *Y* = Bottom + (Top – Bottom)/(1 + 10^((*X* – LogIC50))) using Prism 7 software (GraphPad, San Diego, CA) and pIC_50_ (–logIC_50_) values were extracted from the fitting. *K*_b_ value of the compounds was estimated as described above for functional assays of cAMP signalling at D_2_ receptors.

### Molecular modelling

Compounds **1**–**16** were modelled using LigPrep protocol within Schrödinger software, version 2021-4^43^. Protonation of the studied ligands was performed with Epik module^44^^,^^45^ of the same suite of software. The crystallographic structures of appropriate receptors were used for docking, as follows: inactive conformation of dopamine D_2_ receptor in complex with antagonist risperidone (PDB ID: 6CM4)^46^, active conformation of serotonin 5-HT_1A_ receptor in complex with aripiprazole (PDB ID: 7E2Z)^47^, and inactive state of serotonin 5-HT_2A_ receptor in complex with antagonist risperidone (PDB ID: 6A93)^48^. The structures were retrieved from the PDB database and were modelled by introducing necessary mutations and incorporating missing residues in extracellular loops. Subsequently, the protein structures were preprocessed with Protein Preparation Wizard of Schrödinger suite^49^^,^^50^, as previously described^51^.

Generated grids were centred on co-crystallised ligands. The hydroxyl groups of the following amino acids were set flexible for running molecular docking: Ser 121 (3.39), Ser 193 (5.43), Ser 197, Ser 409 (7.35), Tyr 408 (7.34), Tyr 416 (7.42), Thr 119 (3.37), and Thr 412 (7.38) for D_2_ receptor, Ser 199 (5.43), Tyr 96 (2.63), Tyr 390 (7.42), Thr 121 (3.37), Thr 188, Thr 200 (5.44), and Thr 379 (7.31) for 5-HT_1A_ receptor, and Ser 131 (2.60), Ser 159 (3.36), Ser 242, Tyr 139, Tyr 370 (7.42), Thr 134 (2.63), and Thr 160 (3.37) for 5-HT_2A_ receptor. Glide module^52^ of the Schrödinger suite was used for docking the ligands to the prepared receptor structures. The standard precision (SP) method was applied, and for each ligand–protein complex, the output file containing 50 poses was generated. The final poses of the ligands in obtained complexes were chosen based on the docking score function of glide and visual assessment. For the visualisation of the molecular docking results, the Maestro environment of the Schrödinger suite was used^53^.

PAINS analysis of compounds **1**–**16** was performed with Canvas v. 4.2^54,^^55^.

### X-ray studies

The single-crystal X-ray analysis was performed on an Oxford Diffraction Xcalibur CCD diffractometer (Abingdon, Oxfordshire, UK) using the graphite-monochromated MoKα radiation (*λ* = 0.71073 Å). The CrysAlis software^56^ was applied for data collection, integration and data reduction. The crystal was kept at 293 K during data collection. Using WinGX^57^, the structure was solved by direct method using SHELXS-2018 and refined with the SHELXL-2018^58^. The CrysAlis software was used for multi-scan absorption correction. All non-H atoms were refined with the anisotropic displacement parameters. The H-atoms attached to carbon were positioned geometrically and allowed to ride on the parent atoms with *U*_iso_(H) = 1.2–1.5*U*_eq_(C). The nitrogen-bound hydrogen atoms were located from the different Fourier maps and refined isotropically except H8N. The final data collection parameters and refinement are reported in Table S3 (Supplementary Information). The molecular plots were drawn with OLEX2^59^ and Mercury^60^. The PLATON program^61^ was used for geometrical calculations. The CIF file refinement can be retrieved from the Cambridge Crystallographic Data Centre (CCDC).

### Behavioural studies

#### Drugs

Drugs that were selected for the behavioural studies (i.e. compound **1** and compound **10**) were dissolved in DMSO (final concentration of 0.5%), and diluted by methylcellulose (0.5%, aqueous solution). Both compounds were administered in the intraperitoneal (i.p.) injections at the volume of 10 mL/kg. Compound **1** was administered at the doses of 10, 20, or 40 mg/kg (for spontaneous locomotor activity and rota-rod motor coordination measurements) and at the dose of 10 mg/kg (for all further behavioural testing). Compound **10** was administered at the doses of 20, 40, or 80 mg/kg (for spontaneous locomotor activity and rota-rod motor coordination measurements) and at the dose of 40 mg/kg (for all further behavioural testing). Amphetamine was dissolved in sterile saline and administered subcutaneously (s.c.) at the dose of 5 mg/kg and at the same volume as compounds **1** and **10**. The selected dose was chosen based on our previously published experiments^12^^,^^40^^,^^62^^,^^63^. All solutions (drugs’ and vehicle) were freshly prepared each day of the experiments. Control groups were administered with vehicle injections at the same volume and by the same route of administration as corresponding drugs (i.p. or s.c., 10 mL/kg).

#### Animals

For all behavioural experiments, naïve Swiss male mice were used. Animals were purchased from the Centre of Experimental Medicine of the Medical University of Lublin and, at the time of testing, were approximately 8 weeks old and weighed 20–30 g. Mice were housed in individually ventilated cages (IVCs) system in groups of 4–5 per cage. The animals were kept and maintained under standard laboratory conditions (12-h light/dark cycle, lights on 8.00 a.m., room temperature of 21 ± 1 °C, relative humidity of 50 ± 5%) with free access to tap water and a laboratory chow (Agropol, Ozarow Mazowiecki, Poland). To comply with the mice circadian rhythm, the experiments were conducted between 8.30 a.m. and 2.30 p.m. All experiments were performed in accordance with the National Institute of Health Guidelines for the Care and Use of Laboratory Animals and the European Community Council Directive for Care and Use of Laboratory Animals (2010/63/EU) and received an approval of the local ethics committee (permission number: 147/2018).

#### Behavioural assay

**Figure 12.**
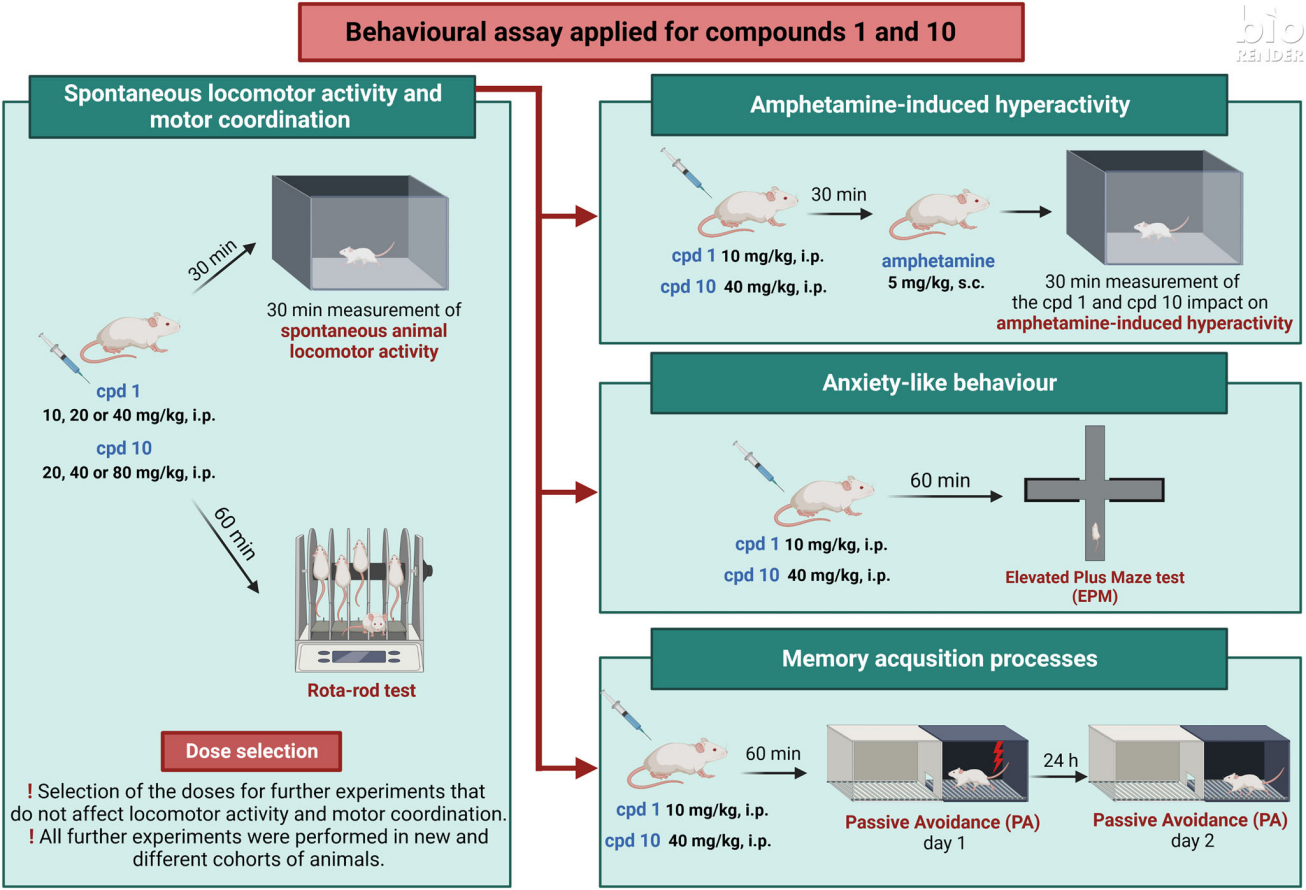
Graphical representation of the applied protocol for behavioural experiments.

All tests performed in the behavioural part of the presented research have been previously performed and validated in our laboratory^12^^,^^40^^,^^63^. The summary of behavioural experiments is depicted in Figure 12.

#### Effects of compound 1 and compound 10 on mice’ spontaneous locomotor activity and amphetamine-induced hyperactivity

To evaluate the effects of compound **1** and compound **10** on mice locomotor activity, an animal activity metre Opto-Varimex-4 Auto-Track (Columbus Instruments, Columbus, OH) was utilised. The measurements of the distance travelled by each mouse were carried out by The Auto-Track System senses motion that was equipped with infra-red photocells. For the assessment of mice’ spontaneous locomotor activity, animals were injected with compound **1** (10, 20, or 40 mg/kg, i.p.), compound **10** (20, 40, or 80 mg/kg, i.p.), or vehicle 30 min prior to testing. After this time, mice were put into the apparatus for 30 min measurement of locomotion. The doses that did not impair animals’ mobility (10 mg/kg, i.p. (compound **1**) and 40 mg/kg, i.p. (compound **10**)) were selected for the assessment of their effects on amphetamine-induced hyperactivity. Briefly, mice were first injected with compound **1** (10 mg/kg, i.p.), compound **10** (40 mg/kg, i.p.), or vehicle. After 30 min, animals received a second injection and were treated with amphetamine (5 mg/kg, s.c.) and were immediately placed in the apparatus for 30 min locomotion evaluation. In both variants of the experiment, the distance travelled by each animal was measured in cm ± SEM.

#### Effects of compound 1 and compound 10 on mice’ motor coordination

To fully assess the impact of selected compounds on animals’ motor coordination, both compounds (at the same doses as were used for spontaneous locomotor activity measurements) were tested also in the rota-rod test. The applied protocol has been already validated in our laboratory^12^^,^^62^^,^^63^. Mice were injected with compound **1** (10, 20, or 40 mg/kg, i.p.), compound **10** (20, 40, or 80 mg/kg, i.p.), or vehicle and, after 60 min, were subjected to the rota-rod test. The time spent by each mouse on the rota-rod was measured for 3 min. The additional aim of the rota-rod test was to confirm the appropriate selection of doses for further behavioural examination. Precisely, it had to be verified whether the doses that did not impair animals’ locomotor activity (compound **1**–10 mg/kg, i.p., compound **10**–40 mg/kg, i.p.) also did not have an influence on animals’ motor coordination. After such confirmation, the above-mentioned doses were chosen for the rest of behavioural tests.

#### Effects of compound 1 and compound 10 on mice’ memory performance

To evaluate the effects of compound **1** and compound **10** on the acquisition of memory processes, the PA test was performed. For the PA test, a two-compartment apparatus (divided by a guillotine door) was used. The compartments differed in colours and in level of illumination–one compartment was white and illuminated with fluorescent light (8 W) and the other was black, fully darkened. The PA apparatus consists also of a grid floor that enables an electric foot shock. To test the impact of compound **1** and compound **10** mice’ on memory performance, animals were injected with compound **1** (10 mg/kg, i.p.), compound **10** (40 mg/kg, i.p.), or vehicle and after 60 min were subjected to PA test. Mice were placed into the illuminated compartment (with guillotine doors closed) for 30 s of habituation time. After this time, the guillotine doors were opened and the time after which a mouse entered a darkened compartment was measured as TL1. Immediately after the entrance to the darkened compartment, the guillotine doors were closed and animals received electric foot shock (0.2 mA for 2 s). Twenty-four hours later, animals (that did not receive any treatment) were put into the illuminated compartment of the apparatus (with guillotine doors opened) and the time to enter the darkened compartment was measured as TL2. The difference in time spent to enter the dark compartment during both days is defined as the latency index and was calculated as IL = (TL2 – TL1)/TL1. An improvement in memory performance is expressed as an increase in the value of the latency index.

#### Effects of compound 1 and compound 10 on mice’ anxiety level

In order to evaluate the impact of compound **1** and compound **10** on anxiety-like behaviour in mice, a classic EPM was performed. The EPM apparatus (38.5 cm high) consists of two open arms (30 × 5 cm) and two closed arms (30 × 5 × 15 cm) that were crossed with a central square platform (5 × 5 cm). The EPM test was performed in the darkened room illuminated only with a red light. Mice were injected with compound **1** (10 mg/kg, i.p.), compound **10** (40 mg/kg, i.p.), or vehicle and, after 60 min, were placed into the central square of EPM apparatus (facing one of the open arms). The test lasted for 5 min and mice’ behaviour was observed and measured by a treatment-blind observer. Precisely, the analysis consisted of the measurements of the number of entries and the time spent in the open and closed arms. The entry into the closed or open arm was defined as a position of the animal with all four paws placed past the central square line. Time spent in open arms and number of entries into open arms were calculated and has been shown as percentages. An anxiolytic-like behaviour was defined as an increase of the time spent in the open arms or/and in the number of entries into the open arms.

## Supplementary Material

Supplemental Material

## Data Availability

The data that support the findings of this study are available in the Supplementary Information of this article.

## References

[1] Bakhshi K, Chance SA. Neuroscience. 2015;303:82–102. 26116523 10.1016/j.neuroscience.2015.06.028

[2] Stępnicki P, Kondej M, Kaczor AA. Current concepts and treatments of schizophrenia. Molecules. 2018;23(8):2087. 30127324 10.3390/molecules23082087 PMC6222385

[3] “Schizophrenia”. World Health Organization; 2016 [accessed 2016 Apr] [Internet]. http://www.who.int/mediacentre/factsheets/fs397/en/.

[4] Marder SR, Cannon TD. Schizophrenia. N Engl J Med. 2019;381(18):1753–1761. 31665579 10.1056/NEJMra1808803

[5] Howes OD, Kapur S. The dopamine hypothesis of schizophrenia: version III–the final common pathway. Schizophr Bull. 2009;35(3):549–562. 19325164 10.1093/schbul/sbp006 PMC2669582

[6] Kane JM. A new treatment paradigm: targeting trace amine-associated receptor 1 (TAAR1) in schizophrenia. J Clin Psychopharmacol. 2022;42(5 Suppl. 1):S1–S13. 36099402 10.1097/JCP.0000000000001596

[7] Miller GM. The emerging role of trace amine-associated receptor 1 in the functional regulation of monoamine transporters and dopaminergic activity. J Neurochem. 2011;116(2):164–176. 21073468 10.1111/j.1471-4159.2010.07109.x PMC3005101

[8] Tonelli M. The breakthrough of TAAR1 agonists for the treatment of neuropsychiatric disorders: one step away. Curr Med Chem. 2022;29(29):4893–4895. 35170404 10.2174/0929867329666220216111512

[9] Ginovart N, Kapur S. Role of dopamine D(2) receptors for antipsychotic activity. Handb Exp Pharmacol. 2012;(212):27–52. 10.1007/978-3-642-25761-2_2 23129327

[10] Lameh J, Burstein ES, Taylor E, Weiner DM, Vanover KE, Bonhaus DW. Pharmacology of N-desmethylclozapine. Pharmacol Ther. 2007;115(2):223–231. 17583355 10.1016/j.pharmthera.2007.05.004

[11] Kondej M, Stępnicki P, Kaczor AA. Multi-target approach for drug discovery against schizophrenia. Int J Mol Sci. 2018;19(10):3105. 30309037 10.3390/ijms19103105 PMC6213273

[12] Kaczor AA, Targowska-Duda KM, Stępnicki P, Silva AG, Koszła O, Kędzierska E, Grudzińska A, Kruk-Słomka M, Biała G, Castro M. N-(3-{4-[3-(trifluoromethyl)phenyl]piperazin-1-yl}propyl)-1H-indazole-3-carboxamide (D2AAK3) as a potential antipsychotic: in vitro, in silico and in vivo evaluation of a multi-target ligand. Neurochem Int. 2021;146:105016. 33722679 10.1016/j.neuint.2021.105016

[13] Gibson MS, Bradshaw RW. The Gabriel synthesis of primary amines. Angew Chem Int Ed Engl. 1968;7(12):919–930.

[14] Fukuyama K, Motomura E, Okada M. Therapeutic potential and limitation of serotonin type 7 receptor modulation. Int J Mol Sci. 2023;24(3):2070. 36768393 10.3390/ijms24032070 PMC9916679

[15] Ballesteros JA, Weinstein H. Integrated methods for the construction of three-dimensional models and computational probing of structure–function relations in G protein-coupled receptors. In: Sealfon SC, editor. Methods in Neurosciences. Vol. 25. Academic Press; 1995. p. 366–428.

[16] Groom CR, Bruno IJ, Lightfoot MP, Ward SC. The Cambridge Structural Database. Acta Crystallogr B Struct Sci Cryst Eng Mater. 2016;72(Pt 2):171–179. 10.1107/S2052520616003954 PMC4822653 27048719

[17] Allen FH, Watson DG, Brammer L, Orpen AG, Taylor R. Typical interatomic distances: organic compounds. In: Prince E, editor. International Tables for Crystallography Volume C: Mathematical, physical and chemical tables. Dordrecht: Springer; 2006. p. 790–811.

[18] Moraski GC, Oliver AG, Markley LD, Cho S, Franzblau SG, Miller MJ. Scaffold-switching: an exploration of 5,6-fused bicyclic heteroaromatics systems to afford antituberculosis activity akin to the imidazo[1,2-a]pyridine-3-carboxylates. Bioorg Med Chem Lett. 2014;24(15):3493–3498. 24909079 10.1016/j.bmcl.2014.05.062 PMC4096046

[19] Kuang S, Zhang P, Dong EZ, Jennings G, Zhao B, Pierce M. Crystal form control and particle size control of RG3487, a nicotinic α7 receptor partial agonist. Int J Pharm. 2016;508(1–2):109–122. 27167333 10.1016/j.ijpharm.2016.04.066

[20] Enguehard-Gueiffier C, Hübner H, El Hakmaoui A, Allouchi H, Gmeiner P, Argiolas A, Melis MR, Gueiffier A. 2-[(4-Phenylpiperazin-1-yl)methyl]imidazo(di)azines as selective D4-ligands. Induction of penile erection by 2-[4-(2-methoxyphenyl)piperazin-1-ylmethyl]imidazo[1,2-a]pyridine (PIP3EA), a potent and selective D4 partial agonist. J Med Chem. 2006;49(13):3938–3947. 16789750 10.1021/jm060166w

[21] Żesławska E, Szymańska E, Nitek W, Handzlik J. Crystallographic studies of piperazine derivatives of 3-methyl-5-spiro­fluorenehydantoin in search of structural features of P-gp inhibitors. Acta Crystallogr C Struct Chem. 2021;77(Pt 8):467–478. 34350844 10.1107/S2053229621006756

[22] Bosc J-J, Jarry C, Léger J-M, Carpy A. NMR and crystallographic evidence for polymorphism of the N-phenyl-N′-[1-(3-(phenyl-4-piperazinyl)propan-2-ol)]urea. J Chem Crystallogr. 1996;26(12):807–814.

[23] Şahin ZS, Yarım M, Köksal M. Density functional computational and X-ray studies on pharmaceutical compound 1-{3-[4-(4-fluorophenyl)piperazin-1-yl]propyl}-1H-indole. Eur J Chem. 2017;8(1):1–7.

[24] Kossakowski J, Hejchman E, Wolska I. Synthesis and structural characterization of aminoalkanol derivatives of 2,3-dihydro-2,2-dimethyl-7-benzofuranol with an expected β-adrenolytic and/or anxiolytic activity. Z Für Naturforschung B. 2002;57(3):285–294.

[25] Xu W, Jiang R, Yuan M. Synthesis, crystal structure, biological evaluation, and molecular docking studies of quinoline-arylpiperazine derivative as potent α1A-adrenoceptor antagonist. J Mol Struct. 2017;1130:895–900.

[26] Bernstein J, Davis RE, Shimoni L, Chang N-L. Patterns in hydrogen bonding: functionality and graph set analysis in crystals. Angew Chem Int Ed Engl. 1995;34(15):1555–1573.

[27] Cremer D, Pople JA. General definition of ring puckering coordinates. J Am Chem Soc. 1975;97(6):1354–1358.

[28] Boeyens JCA. The conformation of six-membered rings. J Cryst Mol Struct. 1978;8(6):317–320.

[29] Haasnoot CAG. The conformation of six-membered rings described by puckering coordinates derived from endocyclic torsion angles. J Am Chem Soc. 1992;114(3):882–887.

[30] Gobira PH, Ropke J, Aguiar DC, Crippa JAS, Moreira FA. Animal models for predicting the efficacy and side effects of antipsychotic drugs. Rev Bras Psiquiatr. 2013;35(Suppl. 2):S132–S139. 24271225 10.1590/1516-4446-2013-1164

[31] Costall B, Domeney AM, Naylor RJ. Locomotor hyperactivity caused by dopamine infusion into the nucleus accumbens of rat brain: specificity of action. Psychopharmacology. 1984;82(3):174–180. 6144126 10.1007/BF00427768

[32] Cools AR. Mesolimbic dopamine and its control of locomotor activity in rats: differences in pharmacology and light/dark periodicity between the olfactory tubercle and the nucleus accumbens. Psychopharmacology. 1986;88(4):451–459. 3085132 10.1007/BF00178506

[33] Kołaczkowski M, Mierzejewski P, Bienkowski P, Wesołowska A, Newman-Tancredi A. Antipsychotic, antidepressant, and cognitive-impairment properties of antipsychotics: rat profile and implications for behavioral and psychological symptoms of dementia. Naunyn Schmiedebergs Arch Pharmacol. 2014;387(6):545–557. 24599316 10.1007/s00210-014-0966-4 PMC4019826

[34] Zhang G, Stackman RW. The role of serotonin 5-HT2A receptors in memory and cognition. Front Pharmacol. 2015;6:225. 26500553 10.3389/fphar.2015.00225 PMC4594018

[35] Overstreet DH, Commissaris RC, De La Garza R, File SE, Knapp DJ, Seiden LS. Involvement of 5-HT1A receptors in animal tests of anxiety and depression: evidence from genetic models. Stress. 2003;6(2):101–110. 12775329 10.1080/1025389031000111311

[36] Heisler LK, Chu H-M, Brennan TJ, Danao JA, Bajwa P, Parsons LH, Tecott LH. Elevated anxiety and antidepressant-like responses in serotonin 5-HT1A receptor mutant mice. Proc Natl Acad Sci U S A. 1998;95(25):15049–15054. 9844013 10.1073/pnas.95.25.15049 PMC24573

[37] Andrews N, Hogg S, Gonzalez LE, File SE. 5-HT1A receptors in the median raphe nucleus and dorsal hippocampus may mediate anxiolytic and anxiogenic behaviours respectively. Eur J Pharmacol. 1994;264(3):259–264. 7698163 10.1016/0014-2999(94)00473-0

[38] Selent J, Marti-Solano M, Rodríguez J, Atanes P, Brea J, Castro M, Sanz F, Loza MI, Pastor M. Novel insights on the structural determinants of clozapine and olanzapine multi-target binding profiles. Eur J Med Chem. 2014;77:91–95. 24631727 10.1016/j.ejmech.2014.02.058

[39] Berg KA, Maayani S, Goldfarb J, Scaramellini C, Leff P, Clarke WP. Effector pathway-dependent relative efficacy at serotonin type 2A and 2C receptors: evidence for agonist-directed trafficking of receptor stimulus. Mol Pharmacol. 1998;54(1):94–104. 9658194

[40] Kaczor AA, Targowska-Duda KM, Silva AG, Kondej M, Biała G, Castro M. N-(2-hydroxyphenyl)-1-[3-(2-oxo-2,3-dihydro-1H-benzimidazol-1-yl)propyl]piperidine-4-carboxamide (D2AAK4), a multi-target ligand of aminergic GPCRs, as a potential antipsychotic. Biomolecules. 2020;10(2):349. 32102432 10.3390/biom10020349 PMC7072648

[41] Kaczor AA, Silva AG, Loza MI, Kolb P, Castro M, Poso A. Structure-based virtual screening for dopamine D2 receptor ligands as potential antipsychotics. ChemMedChem. 2016;11(7):718–729. 26990027 10.1002/cmdc.201500599

[42] Leff P, Dougall IG. Further concerns over Cheng-Prusoff analysis. Trends Pharmacol Sci. 1993;14(4):110–112. 8516953 10.1016/0165-6147(93)90080-4

[43] Schrödinger release 2021–4: LigPrep. New York (NY): Schrödinger, LLC; 2021.

[44] Schrödinger release 2021–4: Epik. New York (NY): Schrödinger, LLC; 2021.

[45] Greenwood JR, Calkins D, Sullivan AP, Shelley JC. Towards the comprehensive, rapid, and accurate prediction of the favorable tautomeric states of drug-like molecules in aqueous solution. J Comput Aided Mol Des. 2010;24(6–7):591–604. 20354892 10.1007/s10822-010-9349-1

[46] Wang S, Che T, Levit A, Shoichet BK, Wacker D, Roth BL. Structure of the D2 dopamine receptor bound to the atypical antipsychotic drug risperidone. Nature. 2018;555(7695):269–273. 29466326 10.1038/nature25758 PMC5843546

[47] Xu P, Huang S, Zhang H, Mao C, Zhou XE, Cheng X, Simon IA, Shen D-D, Yen H-Y, Robinson CV, et al. Structural insights into the lipid and ligand regulation of serotonin receptors. Nature. 2021;592(7854):469–473. 33762731 10.1038/s41586-021-03376-8

[48] Kimura KT, Asada H, Inoue A, Kadji FMN, Im D, Mori C, Arakawa T, Hirata K, Nomura Y, Nomura N, et al. Structures of the 5-HT2A receptor in complex with the antipsychotics risperidone and zotepine. Nat Struct Mol Biol. 2019;26(2):121–128. 30723326 10.1038/s41594-018-0180-z

[49] Schrödinger release 2021–4: protein preparation wizard. New York (NY): Schrödinger, LLC; 2021.

[50] Madhavi Sastry G, Adzhigirey M, Day T, Annabhimoju R, Sherman W. Protein and ligand preparation: parameters, protocols, and influence on virtual screening enrichments. J Comput Aided Mol Des. 2013;27(3):221–234. 23579614 10.1007/s10822-013-9644-8

[51] Patel JZ, Parkkari T, Laitinen T, Kaczor AA, Saario SM, Savinainen JR, Navia-Paldanius D, Cipriano M, Leppänen J, Koshevoy IO, et al. Chiral 1,3,4-oxadiazol-2-ones as highly selective FAAH inhibitors. J Med Chem. 2013;56(21):8484–8496. 24083878 10.1021/jm400923s

[52] Schrödinger release 2021–4: Glide. New York (NY): Schrödinger, LLC; 2021.

[53] Schrödinger release 2021–4: Maestro. New York (NY): Schrödinger, LLC; 2021.

[54] Duan J, Dixon SL, Lowrie JF, Sherman W. Analysis and comparison of 2D fingerprints: insights into database screening performance using eight fingerprint methods. J Mol Graph Model. 2010;29(2):157–170. 20579912 10.1016/j.jmgm.2010.05.008

[55] Sastry M, Lowrie JF, Dixon SL, Sherman W. Large-scale systematic analysis of 2D fingerprint methods and parameters to improve virtual screening enrichments. J Chem Inf Model. 2010;50(5):771–784. 20450209 10.1021/ci100062n

[56] CrysAlis PRO. Version 1.171.37.35g. Yarnton (England): Oxford Diffraction/Agilent Technologies UK Ltd; 2014.

[57] Farrugia LJ. WinGX and ORTEP for Windows: an update. J Appl Crystallogr. 2012;45(4):849–854.

[58] Sheldrick GM. Crystal structure refinement with SHELXL. Acta Crystallogr C Struct Chem. 2015;71(Pt 1):3–8. 25567568 10.1107/S2053229614024218 PMC4294323

[59] Dolomanov OV, Bourhis LJ, Gildea RJ, Howard JAK, Puschmann H. OLEX2: a complete structure solution, refinement and analysis program. J Appl Crystallogr. 2009;42(2):339–341.

[60] Macrae CF, Sovago I, Cottrell SJ, Galek PTA, McCabe P, Pidcock E, Platings M, Shields GP, Stevens JS, Towler M, et al. Mercury 4.0: from visualization to analysis, design and prediction. J Appl Crystallogr. 2020;53(Pt 1):226–235. 32047413 10.1107/S1600576719014092 PMC6998782

[61] Spek AL. Single-crystal structure validation with the program PLATON. J Appl Crystallogr. 2003;36(1):7–13.

[62] Kaczor AA, Targowska-Duda KM, Budzyńska B, Biała G, Silva AG, Castro M. In vitro, molecular modeling and behavioral studies of 3-{[4-(5-methoxy-1H-indol-3-yl)-1,2,3,6-tetrahydropyridin-1-yl]methyl}-1,2-dihydroquinolin-2-one (D2AAK1) as a potential antipsychotic. Neurochem Int. 2016;96:84–99. 26964765 10.1016/j.neuint.2016.03.003

[63] Kondej M, Wróbel TM, Targowska-Duda KM, Leandro Martínez A, Koszła O, Stępnicki P, Zięba A, Paz A, Wronikowska-Denysiuk O, Loza MI, et al. Multitarget derivatives of D2AAK1 as potential antipsychotics: the effect of substitution in the indole moiety. ChemMedChem. 2022;17(15):e202200238. 35610178 10.1002/cmdc.202200238

