# Supplementary material for: Novel multi-target ligands of dopamine and serotonin receptors for the treatment of schizophrenia based on indazole and piperazine scaffolds–synthesis, biological activity, and structural evaluation
Source: J Enzyme Inhib Med Chem. 2023 May 15;38(1):2209828. doi: 10.1080/14756366.2023.2209828 (PMC10187112; doi:10.1080/14756366.2023.2209828)
Supplement: Supplemental Material [file IENZ_A_2209828_SM4266.pdf]

Supplementary Information

Novel multi-target ligands of dopamine and serotonin receptors for the treatment of schizophrenia based on indazole and piperazine scaffolds – synthesis, biological activity and structural evaluation

Piotr Stępnicki[1*], Olga Wronikowska-Denysiuk[2], Agata Zięba[1], Katarzyna M. Targowska-Duda[3], Agata Bartyzel[4], Martyna Z. Wróbel[5], Tomasz M. Wróbel[1], Klaudia Szałaj[6], Andrzej Chodkowski[5], Karolina Mirecka[5], Barbara Budzyńska[2], Emilia Fornal[6], Jadwiga Turło[5], Marián Castro[7,8], Agnieszka A. Kaczor[1,9*]

[1]*Department of Synthesis and Chemical Technology of Pharmaceutical Substances, Faculty of Pharmacy, Medical University of Lublin, 4A Chodźki St., PL-20093 Lublin, Poland*

[2]*Independent Laboratory of Behavioral Studies, Chair of Biomedical Sciences, Faculty of Biomedicine, Medical University of Lublin, 4A Chodźki St., PL-20093 Lublin, Poland*

[3]*Department of Biopharmacy, Faculty of Pharmacy, Medical University of Lublin, 4A Chodźki St., PL-20093 Lublin, Poland*

[4]*Department of General and Coordination Chemistry and Crystallography, Institute of Chemical Sciences, Faculty of Chemistry, Maria Curie-Sklodowska University in Lublin, Maria Curie-Sklodowska Sq. 2, 20-031 Lublin, Poland*

[5]*Department of Drug Technology and Pharmaceutical Biotechnology, Faculty of Pharmacy, Medical University of Warsaw, 1 Banacha Street, 02-097, Warszawa, Poland*

[6]*Department of Bioanalytics, Chair of Dietetics and Bioanalytics, Faculty of Biomedicine, Medical University of Lublin, Jaczewskiego 8b St., 20-090 Lublin, Poland*

[7]Department of Pharmacology, Universidade de Santiago de Compostela, Center for Research in Molecular Medicine and Chronic Diseases (CIMUS), Avda de Barcelona, E-15782 Santiago de Compostela, Spain

[8]Instituto de Investigación Sanitaria de Santiago de Compostela (IDIS), Travesía da Choupana s/n, E-15706 Santiago de Compostela, Spain

[9]School of Pharmacy, University of Eastern Finland, P.O. Box 1627, FI-70211 Kuopio, Finland

**Correspondence: piotrstepnicki93@gmail.com (P.S.), agnieszka.kaczor@umlub.pl (A.A.K.)*

Content:

Fig. S1. Competition radioligand binding curves for compounds **2** – **3**, **5**, **6**, **8**, **9**, **12** – **15**, and reference competitor haloperidol on membranes from CHO-K1 cells stably expressing cloned human D_{2S} receptors.

Fig. S2. Competition radioligand binding curves for compounds **2** – **3**, **5**, **6**, **8**, **9**, **12** – **15**, and reference competitor 5-carboxamidotryptamine (5-CT) on membranes from HEK293 cells stably expressing cloned human 5-HT_{1A} receptors.

Fig. S3. Competition radioligand binding curves for compounds **2** – **6**, **8**, **9**, **12** – **15**, and reference competitor risperidone on membranes from CHO-K1 cells stably expressing cloned human 5-HT_{2A} receptors.

Fig. S4. A fragment of the crystal structure of compound **1** emphasizing the connection of molecules A and B in homodimers A-A and B-B.

Fig. S5. A fragment of the crystal structure of compound **1** showing formation of layers.

Table S1. Interatomic distances and selected bond angles for compound **1**.

Table S2. Detailed experimental conditions employed in radioligand binding assays.

Table S3. Details of data collection and structure refinement parameters for compound **1**.

NMR and HRMS spectra of reported compounds, and HPLC-DAD chromatograms of compounds **1** and **10**.

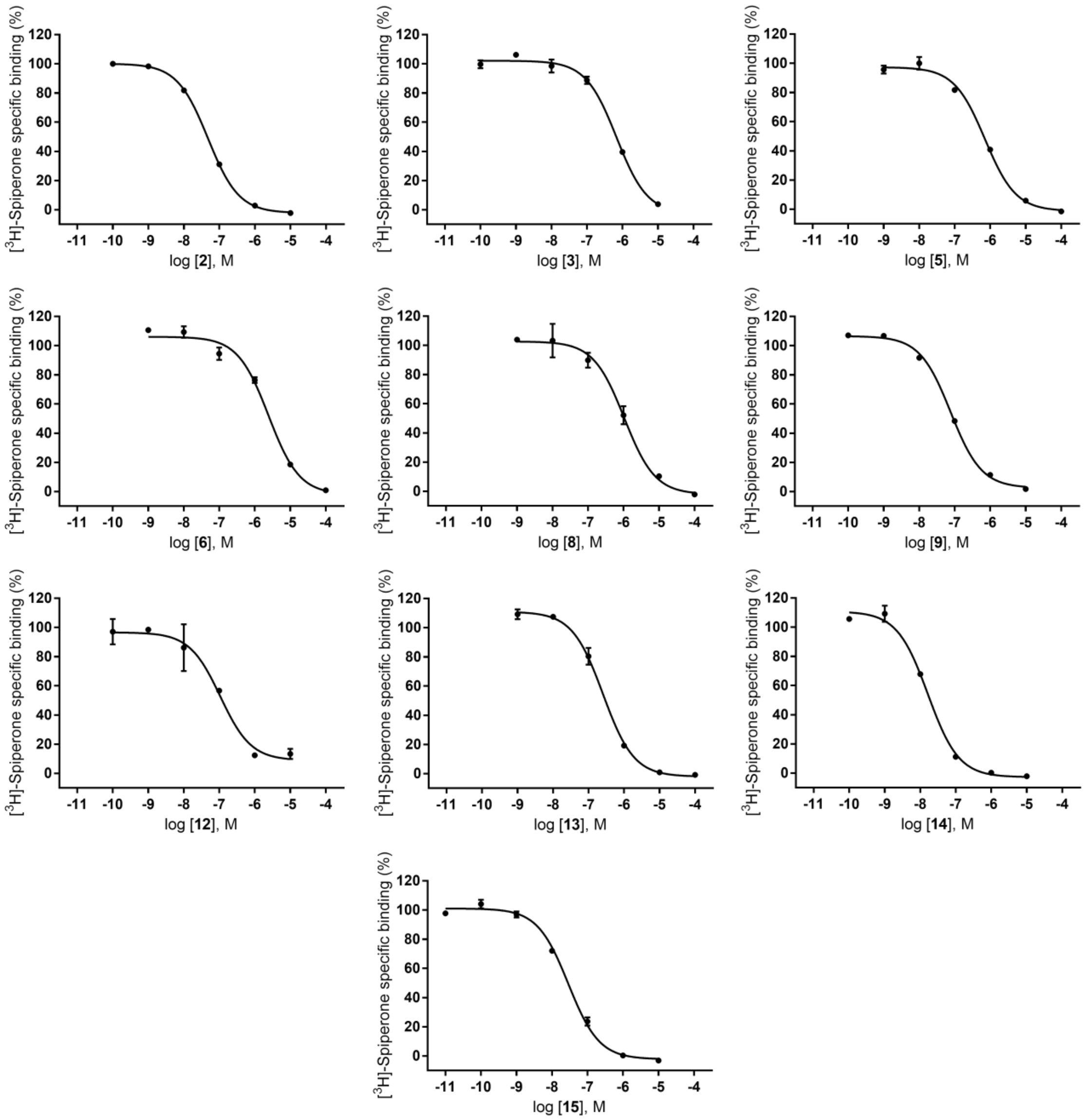

Fig. S1. Competition radioligand binding curves for compounds **2** – **3**, **5**, **6**, **8**, **9**, **12** – **15**, and reference competitor haloperidol on membranes from CHO-K1 cells stably expressing cloned human D_{2S} receptors. Data are expressed as % of radioligand ($[^3H]$-Spiperone) specific binding and curves were fitted to a one-site competition binding model. The graphs show data (mean ± SEM) of a representative experiment out of 2 - 5 independent experiments performed in duplicate.

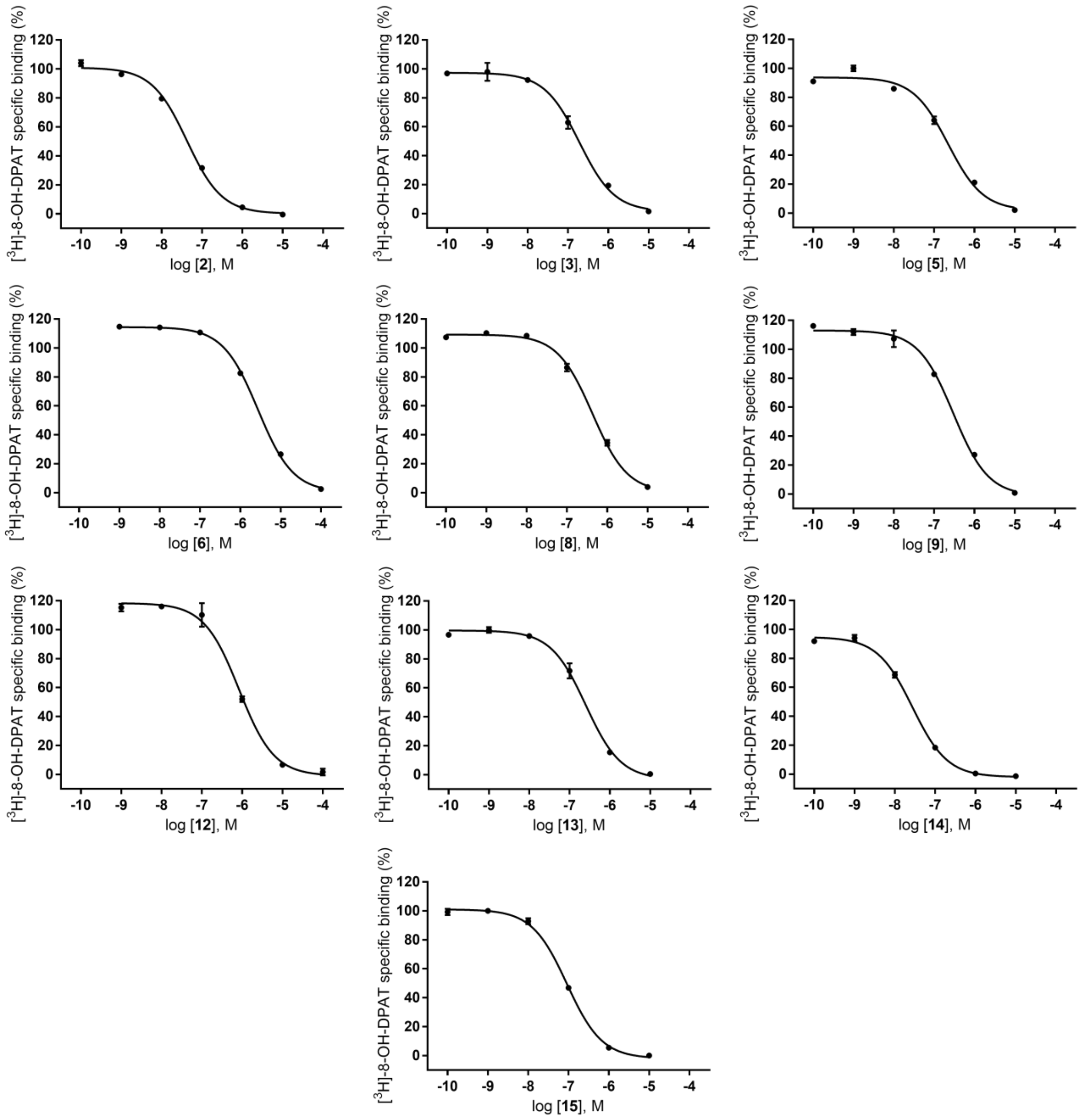

Fig. S2. Competition radioligand binding curves for compounds **2** – **3**, **5**, **6**, **8**, **9**, **12** – **15**, and reference competitor 5-carboxamidotryptamine (5-CT) on membranes from HEK293 cells stably expressing cloned human 5-HT_{1A} receptors. Data are expressed as % of radioligand ([^{3}H]-8-OH-DPAT) specific binding and curves were fitted to a one-site competition binding model. The graphs show data (mean ± SEM) of a representative experiment out of 2 - 3 independent experiments performed in duplicate.

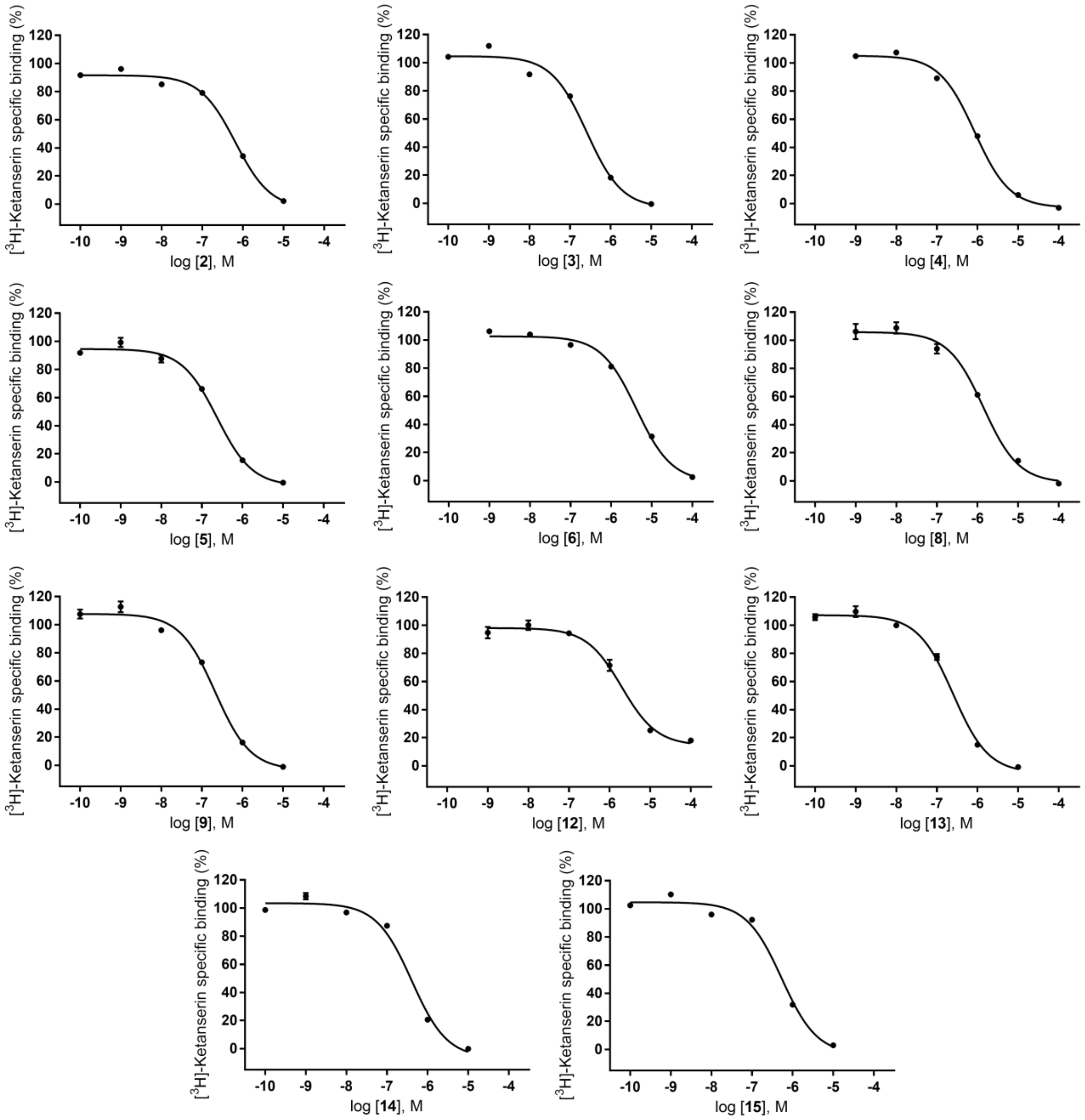

Fig. S3. Competition radioligand binding curves for compounds **2** – **6**, **8**, **9**, **12** – **15**, and reference competitor risperidone on membranes from CHO-K1 cells stably expressing cloned human 5-HT_{2A} receptors. Data are expressed as % of radioligand ([^{3}H]- Ketanserin) specific binding and curves were fitted to a one-site competition binding model. The graphs show data (mean ± SEM) of a representative experiment out of 2 - 5 independent experiments performed in duplicate.

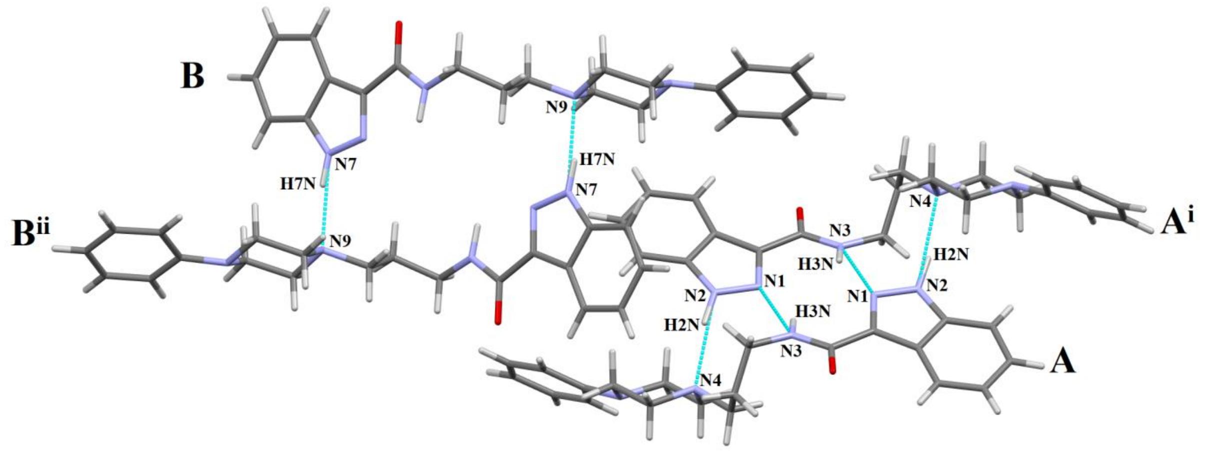

Fig. S4. A fragment of the crystal structure of compound **1** emphasizing the connection of molecules A and B in homodimers A-A and B-B. The molecules of solvent were omitted for clarity.

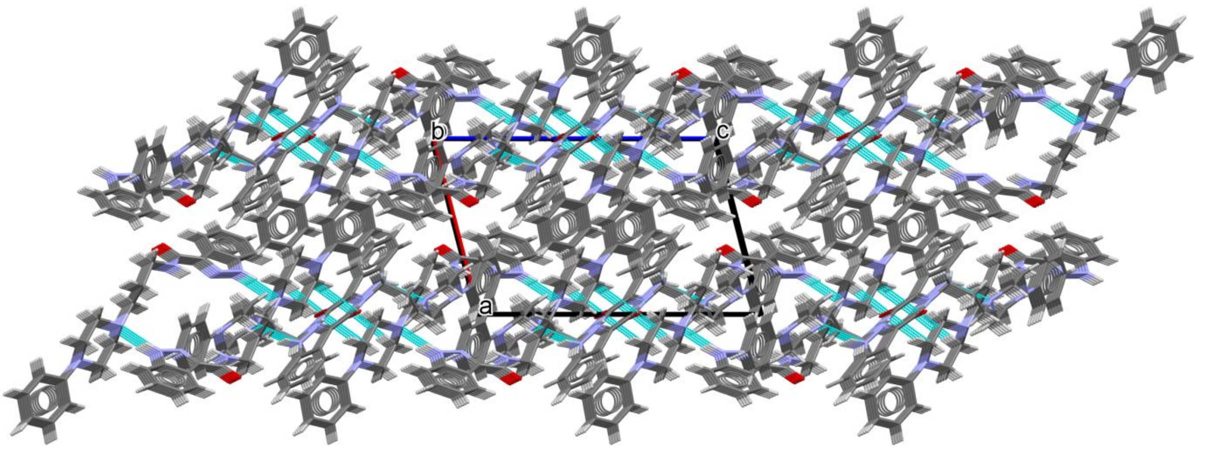

Fig. S5. A fragment of the crystal structure of compound **1** showing formation of layers. The molecules of solvent and hanging contacts were omitted for clarity.

Table S1. Interatomic distances and selected bond angles for compound **1**.

Bond lengths (Å)			
Molecule A			
C(1)-N(1)	1.338(3)	C(11)-N(4)	1.475(3)
C(1)-C(7)	1.426(4)	C(12)-N(4)	1.464(3)
C(1)-C(8)	1.477(4)	C(12)-C(13)	1.508(3)
C(2)-N(2)	1.370(3)	C(13)-N(5)	1.453(3)
C(2)-C(7)	1.392(4)	C(14)-C(15)	1.378(4)
C(2)-C(3)	1.400(4)	C(14)-C(19)	1.396(4)
C(3)-C(4)	1.363(4)	C(14)-N(5)	1.402(3)
C(4)-C(5)	1.390(4)	C(15)-C(16)	1.376(4)
C(5)-C(6)	1.368(4)	C(16)-C(17)	1.364(4)
C(6)-C(7)	1.402(4)	C(17)-C(18)	1.370(4)
C(8)-O(1)	1.224(3)	C(18)-C(19)	1.367(4)
C(8)-N(3)	1.341(3)	C(20)-N(5)	1.457(3)
C(9)-N(3)	1.445(4)	C(20)-C(21)	1.503(4)
C(9)-C(10)	1.507(4)	C(21)-N(4)	1.461(4)
C(10)-C(11)	1.513(4)	N(1)-N(2)	1.360(3)
Molecule B			
C(22)-N(6)	1.331(3)	C(32)-N(9)	1.477(3)
C(22)-C(28)	1.409(4)	C(33)-N(9)	1.460(3)
C(22)-C(29)	1.471(4)	C(33)-C(34)	1.499(4)
C(23)-N(7)	1.368(4)	C(34)-N(10)	1.454(4)
C(23)-C(24)	1.392(4)	C(35)-C(36)	1.389(4)
C(23)-C(28)	1.398(4)	C(35)-C(40)	1.392(4)
C(24)-C(25)	1.370(4)	C(35)-N(10)	1.407(3)
C(25)-C(26)	1.401(5)	C(36)-C(37)	1.366(4)
C(26)-C(27)	1.372(4)	C(37)-C(38)	1.370(5)
C(27)-C(28)	1.397(4)	C(38)-C(39)	1.367(5)
C(29)-O(2)	1.224(4)	C(39)-C(40)	1.385(4)
C(29)-N(8)	1.338(4)	C(41)-N(10)	1.452(3)
C(30)-N(8)	1.444(3)	C(41)-C(42)	1.499(4)
C(30)-C(31)	1.523(4)	C(42)-N(9)	1.464(4)
C(31)-C(32)	1.478(4)	N(6)-N(7)	1.349(3)
Solvent molecule			
C(43)-N(11)	1.100(5)	C(43)-C(44)	1.405(5)
Torsion angles (º)			
N(4)-C(12)-C(13)-N(5)	57.3(3)	N(9)-C(33)-C(34)-N(10)	58.3(4)
N(5)-C(20)-C(21)-N(4)	-57.8(4)	N(10)-C(41)-C(42)-N(9)	-58.3(3)
C(20)-C(21)-N(4)-C(12)	58.9(3)	C(34)-C(33)-N(9)-C(42)	-58.9(4)
C(13)-C(12)-N(4)-C(21)	-58.4(3)	C(41)-C(42)-N(9)-C(33)	59.2(3)
C(12)-C(13)-N(5)-C(20)	-53.4(3)	C(42)-C(41)-N(10)-C(34)	54.6(3)
C(21)-C(20)-N(5)-C(13)	53.6(4)	C(33)-C(34)-N(10)-C(41)	-54.9(4)

Table S2. Detailed experimental conditions employed in radioligand binding assays.

	*h*D$_1$	*h*D$_2$	*h*D$_3$	*h*5-HT$_{1A}$	*h*5-HT$_{2A}$	*h*5-HT$_7$	*h*H$_1$	*h*M$_1$
Protein per well	12 µg	35 µg	2 µg	10 µg	130 µg	2 µg	50 µg	2.5 µg
Assay buffer	50 mM Tris-HCl, 5 mM $MgCl_2$ (pH = 7.4)	50 mM Tris-HCl, 120 mM NaCl, 5 mM KCl, 5 mM $MgCl_2$, 1 mM EDTA (pH = 7.4)	50 mM Tris-HCl, 5 mM $MgCl_2$ (pH = 7.4)	50 mM Tris-HCl, 5 mM $MgSO_4$ (pH = 7.4)	50 mM Tris-HCl (pH = 7.5)	50 mM Tris-HCl, 4 mM $CaCl_2$, 1 mM L-Ascorbic acid, 0.1 mM Pargyline hydrochloride (pH = 7.4)	41.4 mM Na_2HPO_4, 8.6 mM KH_2PO_4 (pH = 7.5)	10 mM HEPES, 100 mM NaCl, 10 mM $MgCl_2$, 0.2% BSA (pH = 7.4)
Radioligand	0.7 nM [^{3}H]-SCH-23390[a]	0.6 nM [^{3}H]-Spiperone[b]	1 nM [^{3}H]-Spiperone[b]	1 nM [^{3}H]-8-OH-DPAT[c]	1.25 nM [^{3}H]-Ketanserin[d]	2 nM [^{3}H]-SB269970[e]	4 nM [^{3}H]-Pyrilamine[f]	7 nM [^{3}H]-Pirenzepine[g]
Nonspecific binding	1 µM (+)-Butaclamol	100 µM Sulpiride	1 µM Haloperidol	10 µM Serotonin	1 µM Methysergide	25 µM Clozapine	10 µM Triprolidine	200 µM Pirenzepine
Incubation	27ºC/60 min	25ºC/120 min	25ºC/60 min	37ºC/120 min	37ºC/30 min	37ºC/60 min	27ºC/60 min	25ºC/90 min
Filter plate	GF/C[h]	GF/C[h]	GF/C[h]	GF/C[h]	GF/B[i]	GF/C[h]	GF/B[i]	GF/C[h]
Filter plate pre-treatment	presoaked with 0.5% PEI[j] for 1 h and washed with 250 µl incubation buffer	presoaked with 0.5% PEI[j] for 1 h and washed with 250 µl incubation buffer	presoaked with 0.5% PEI[j] for 1 h and washed with 250 µl incubation buffer	presoaked with 0.5% PEI[j] for 1 h and washed with 250 µl incubation buffer	presoaked with 0.5% PEI[j] for 1 h and washed with 250 µl incubation buffer	presoaked with 0.5% PEI[j] for 1 h and washed with 250 µl incubation buffer	presoaked with 0.1% Tween20 for 1 h and washed with 250 µl incubation buffer	presoaked with 0.1% Tween20 for 1 h and washed with 250 µl incubation buffer
Wash buffer	50 mM Tris-HCl (pH = 7.4)	50 mM Tris-HCl, 0.9% NaCl (pH = 7.4)	50 mM Tris-HCl (pH = 7.4)	50 mM Tris-HCl (pH = 7.4)	50 mM Tris-HCl (pH = 6.6)	50 mM Tris-HCl, 4 mM $CaCl_2$, 1 mM L-Ascorbic acid, 0.1 mM Pargyline hydrochloride (pH = 7.4)	41.4 mM Na_2HPO_4, 8.6 mM KH_2PO_4 (pH = 7.5)	10 mM HEPES, 0.1% BSA (pH = 7.4)
Washing steps	4 x 250 µl wash buffer	4 x 250 µl wash buffer	4 x 250 µl wash buffer	4 x 250 µl wash buffer	6 x 250 µl wash buffer	4 x 250 µl wash buffer	6 x 250 µl wash buffer	4 x 250 µl wash buffer

[a] 83.2 Ci/mmol, 1 mCi/ml, PerkinElmer NET930250UC. [b] 54.3 Ci/mmol, 1 mCi/ml, PerkinElmer NET1187250UC. [c] 200 Ci/mmol, 1 mCi/ml, PerkinElmer NET929250UC. [d] 47.3 Ci/mmol, 1 mCi/ml, PerkinElmer NET791250UC. [e] 32.7 Ci/mmol, 0.25 mCi/ml, PerkinElmer NET1198U250UC. [f] 20.1 Ci/mmol, 1 mCi/ml, PerkinElmer NET594250UC; [g] 85.0 Ci/mmol, 1 mCi/ml, PerkinElmer NET780250UC. [h] MultiScreen$_{HTS}$ FB Filter Plate, PerkinElmer MSFBN6. [i] MultiScreen$_{HTS}$ FB Filter Plate, PerkinElmer MSFCN6. [j] PEI, Polyethyleneimine.

Table S3. Details of data collection and structure refinement parameters for compound **1**.

CCDC	2236557
Temperature K	293(2)
Crystal system	triclinic
Space group	*P*–1
a (Å)	10.4997(7)
b (Å)	14.1627(11)
c (Å)	15.3117(12)
α (°)	86.183(6)
β(°)	74.924(6)
γ(°)	68.761(7
Volume (Å^3)	2048.2(3)
Z	2
Calculated density (g cm^{-3})	1.245
μ (mm^{-1})	0.080
Absorption correction	multi-scan
F(000)	820
Crystal size (mm)	0.500 x 0.200 x 0.100
θ range (º)	2.757–26.020
Reflections collected/unique	14479/8058
R_{int}	0.0485
Data/restraints/parameters	8058/0/527
GooF on F^2	0.968
Final R indices [I>2σ(I)]	R1 = 0.0641,<br>wR2 = 0.1015
R indices (all data)	R1 = 0.1786,<br>wR2 = 0.1424
Largest diff. peak/hole, e Å^{-3}	0.164/-0.177

N-(3-(4-phenylpiperazin-1-yl)propyl)-1*H*-indazole-3-carboxamide (1)

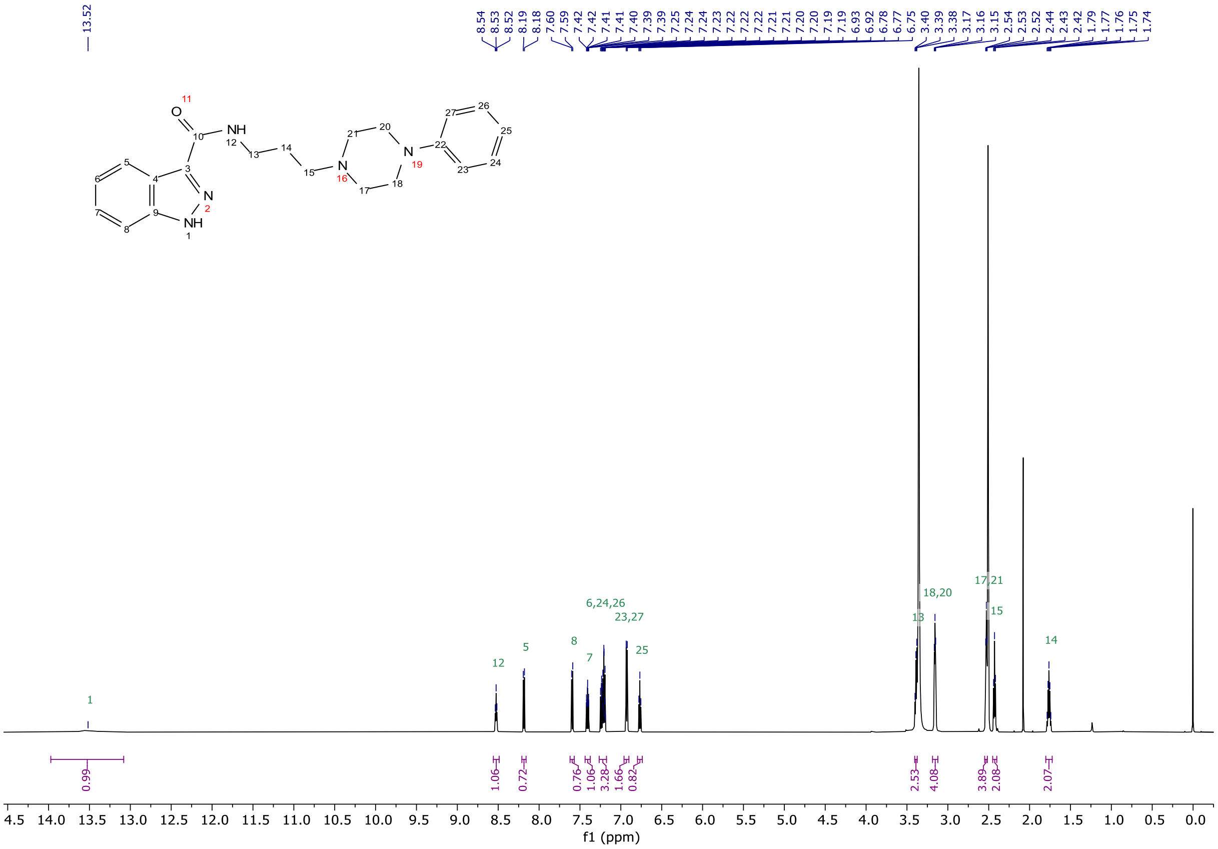

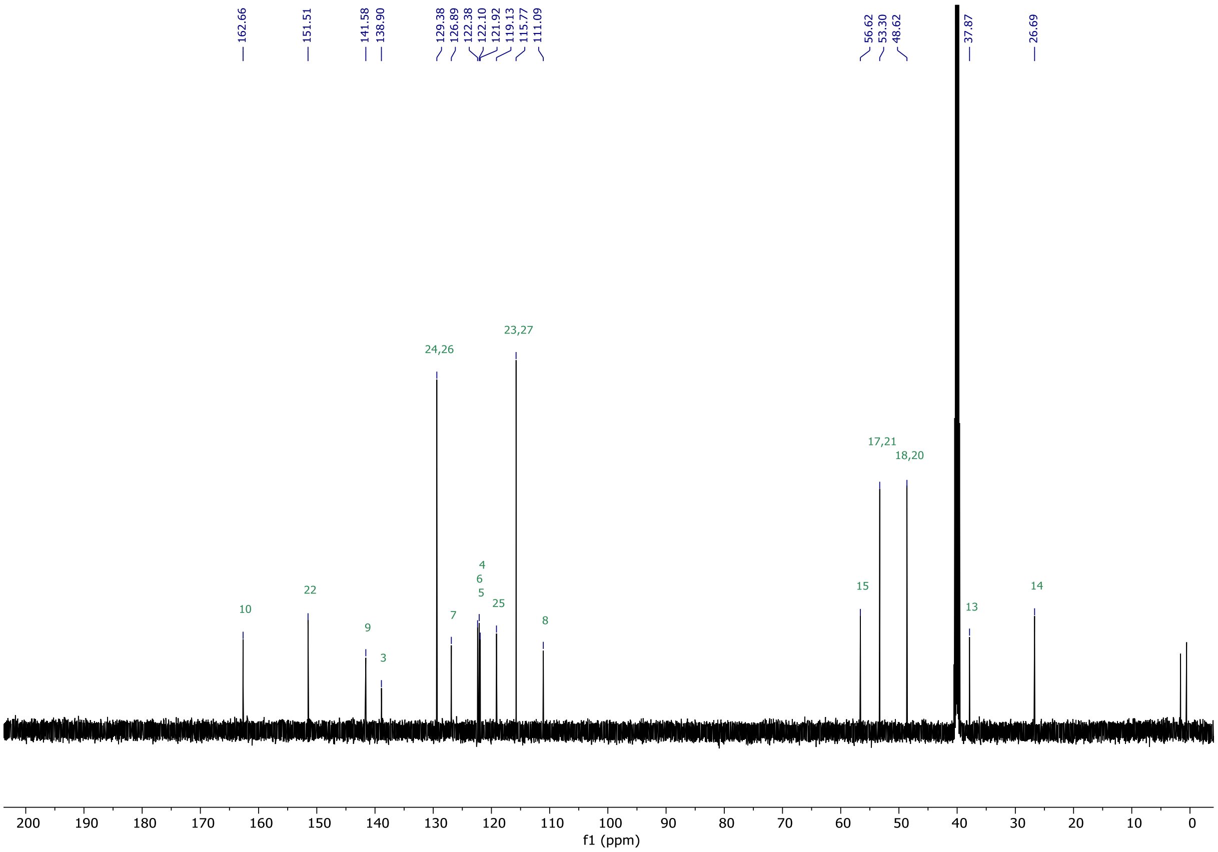

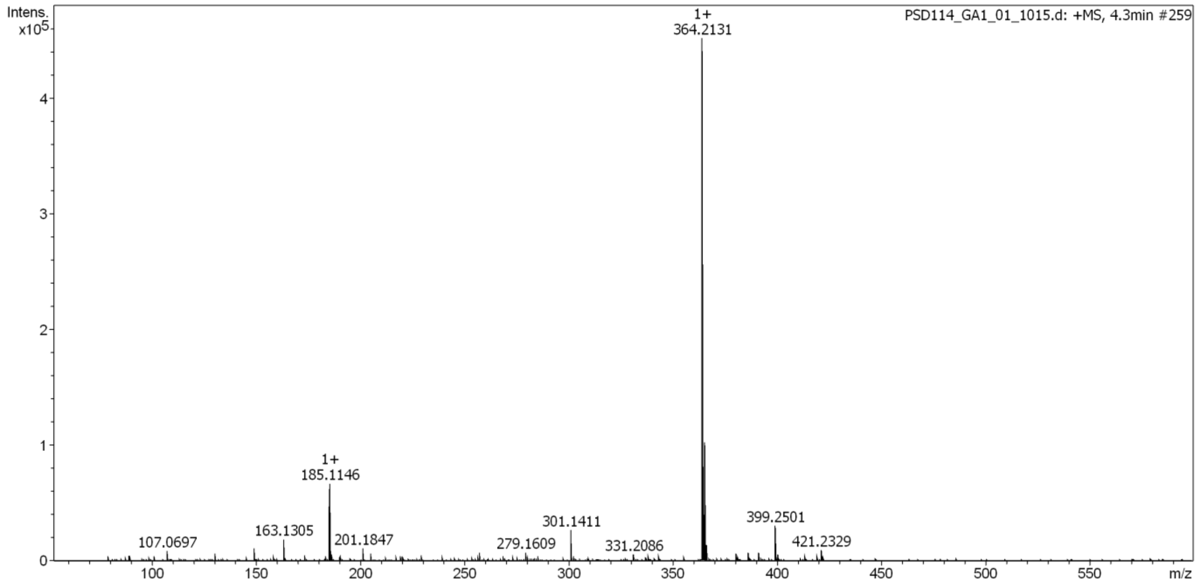

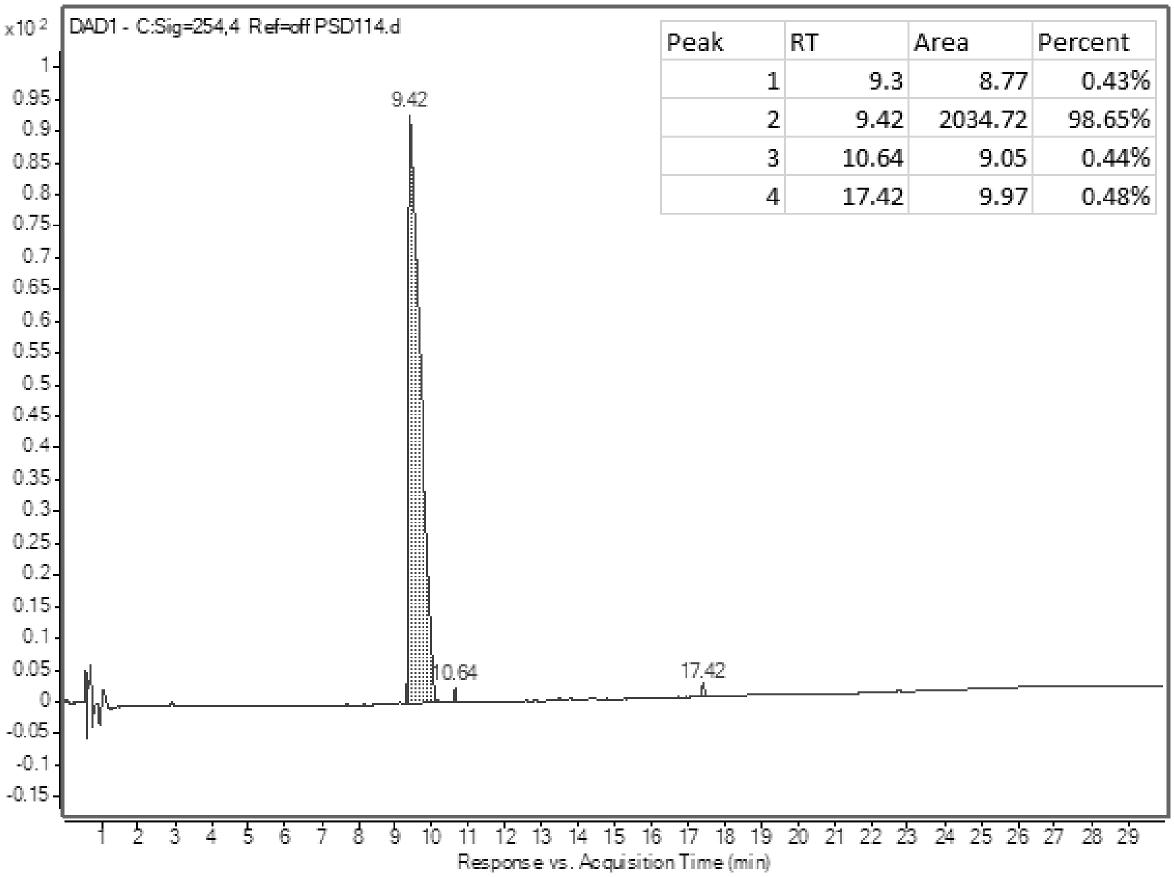

Peak	RT	Area	Percent
1	9.3	8.77	0.43%
2	9.42	2034.72	98.65%
3	10.64	9.05	0.44%
4	17.42	9.97	0.48%

N-(3-(4-(2-methoxyphenyl)piperazin-1-yl)propyl)-1*H*-indazole-3-carboxamide (2)

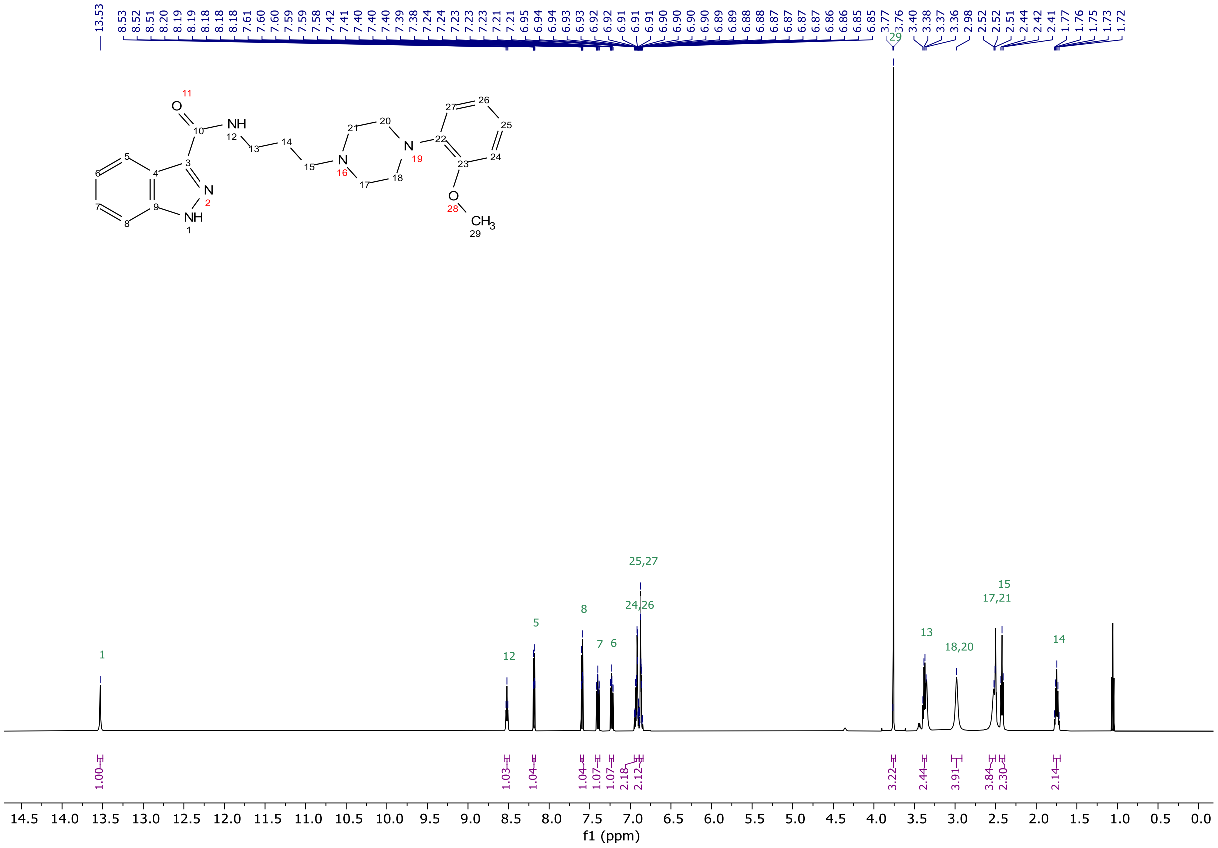

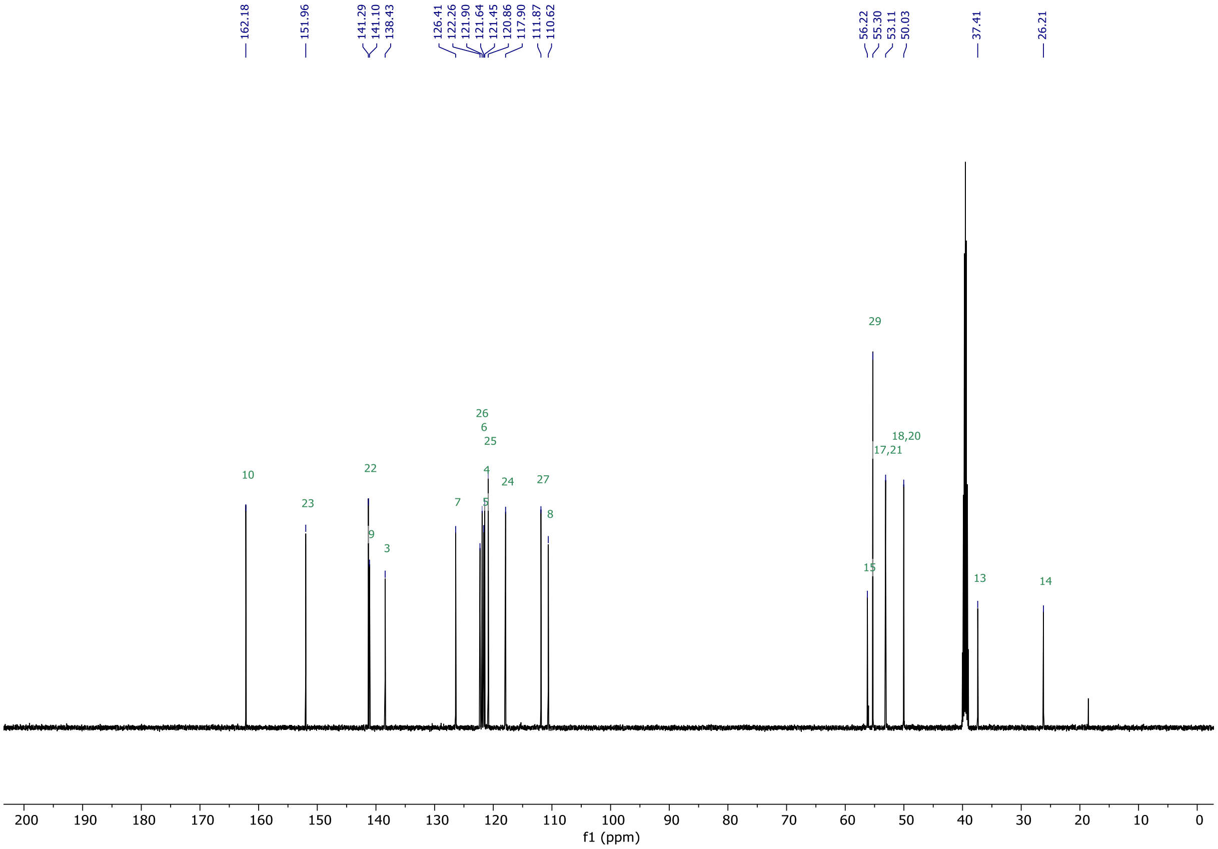

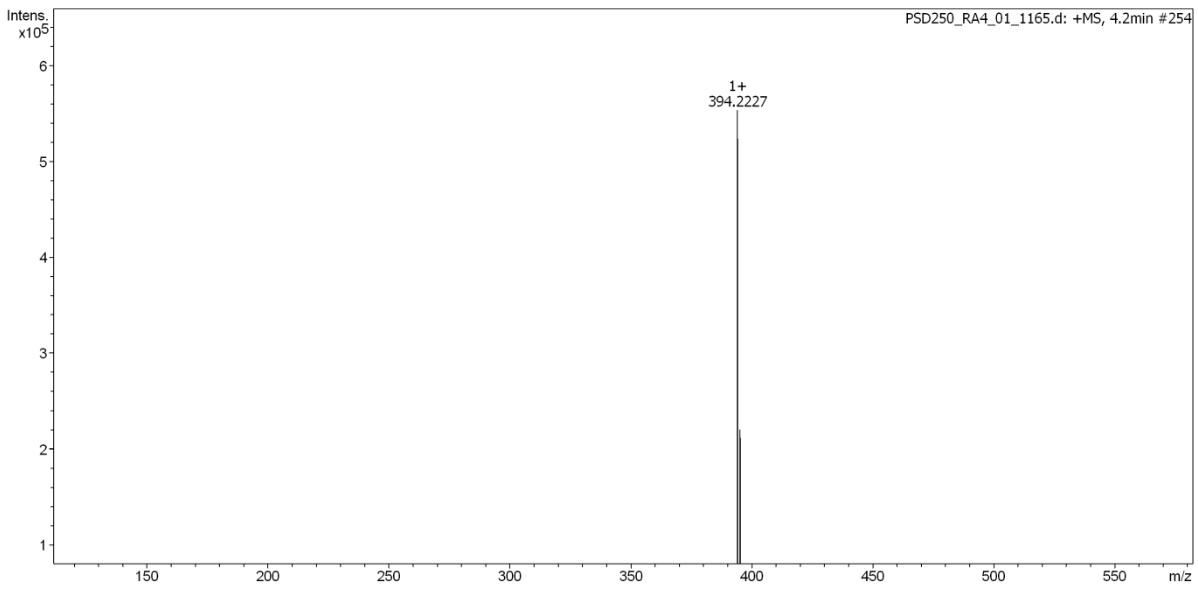
Intens.
x10^5
PSD250_RA4_01_1165.d: +MS, 4.2min #254
1+
394.2227
m/z

N-(3-(4-(3-methoxyphenyl)piperazin-1-yl)propyl)-1H-indazole-3-carboxamide (3)

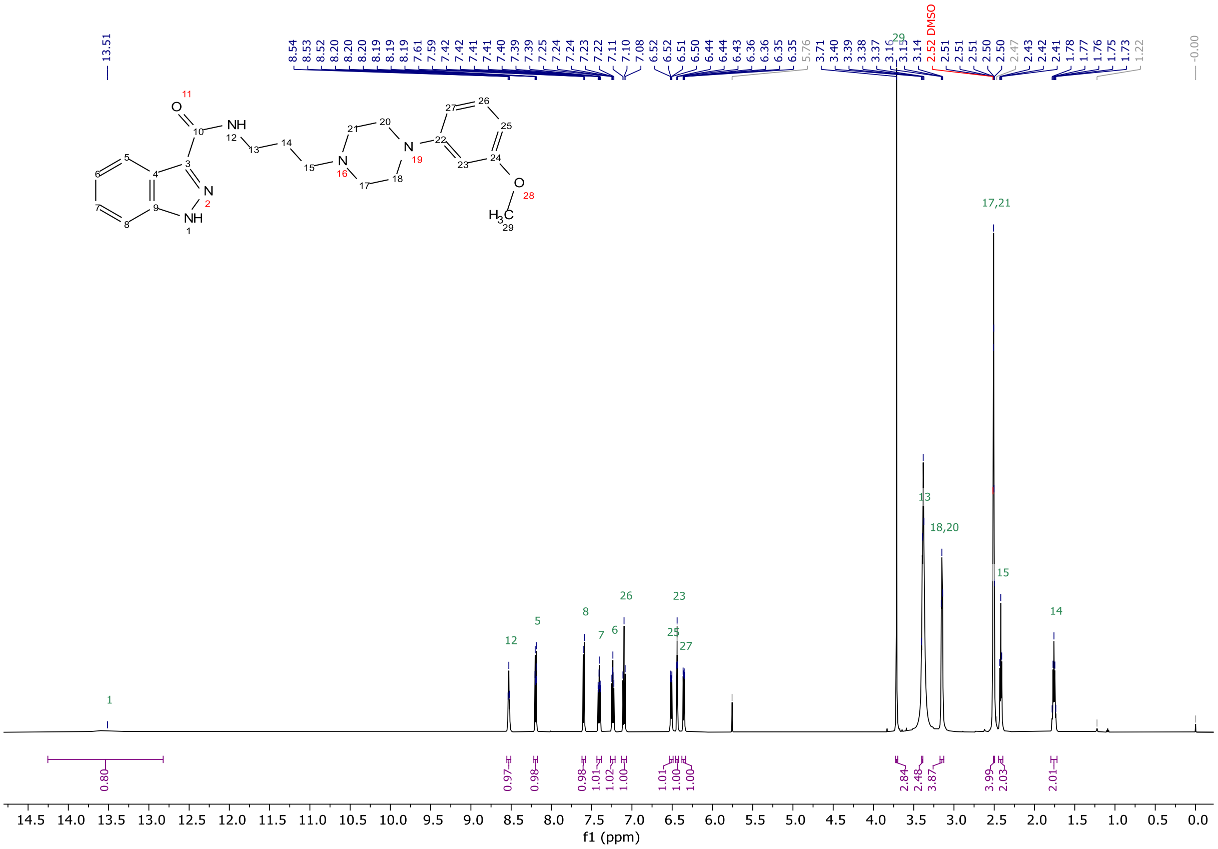

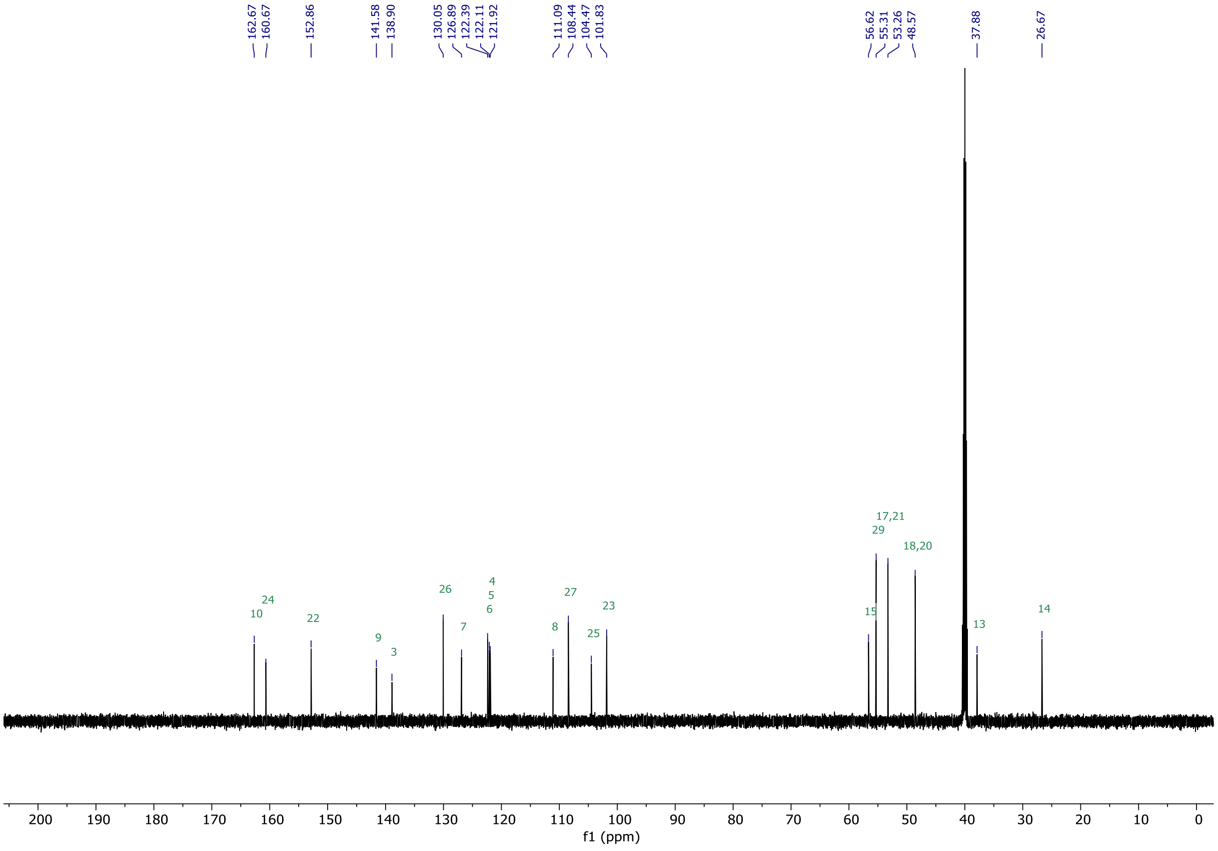

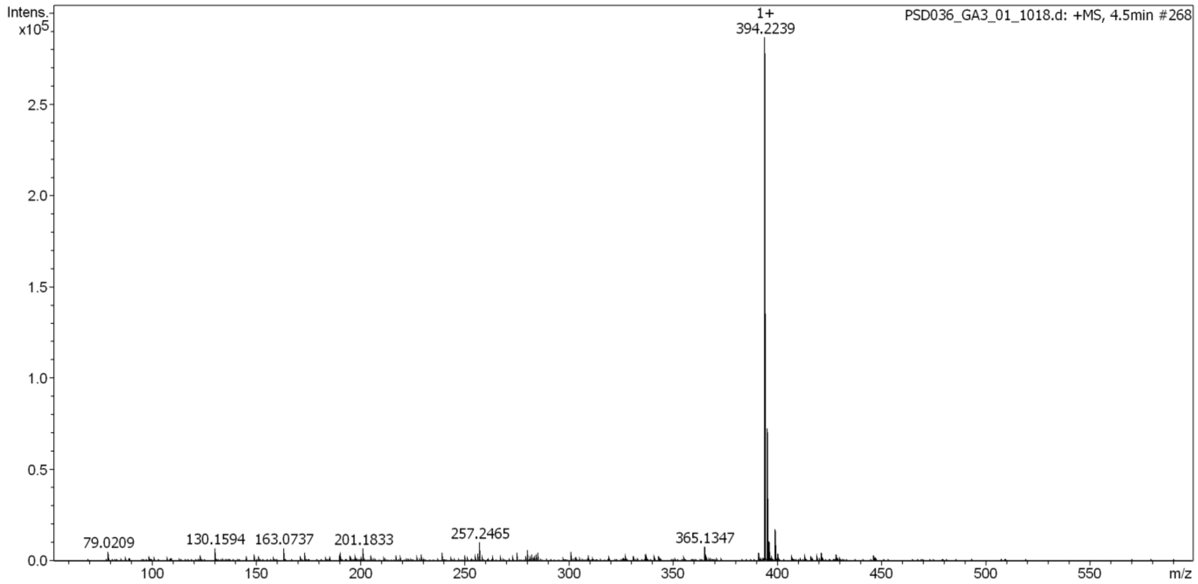

PSD036_GA3_01_1018.d: +MS, 4.5min #268
Intens.
x10^5
1+
394.2239
365.1347
257.2465
201.1833
163.0737
130.1594
79.0209
m/z

***N*-(3-(4-(4-methoxyphenyl)piperazin-1-yl)propyl)-1*H*-indazole-3-carboxamide (4)**

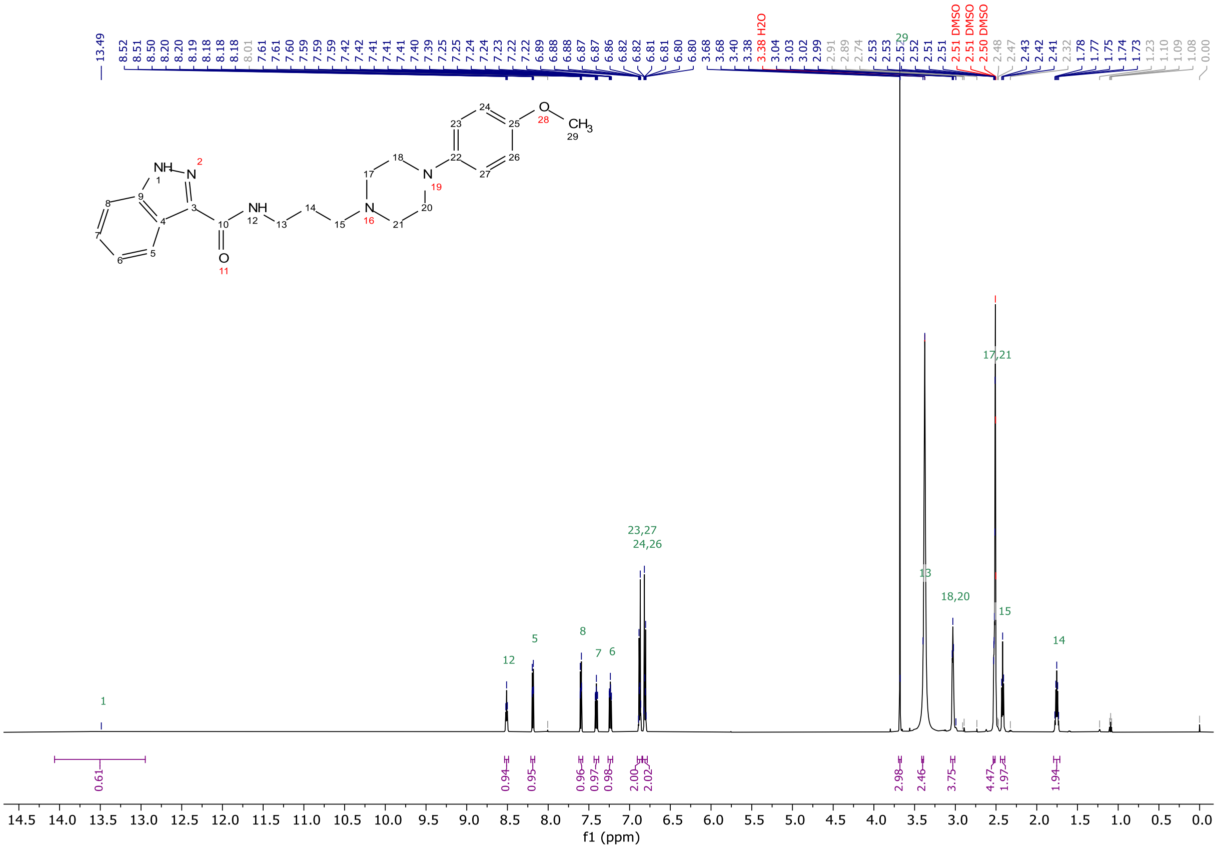

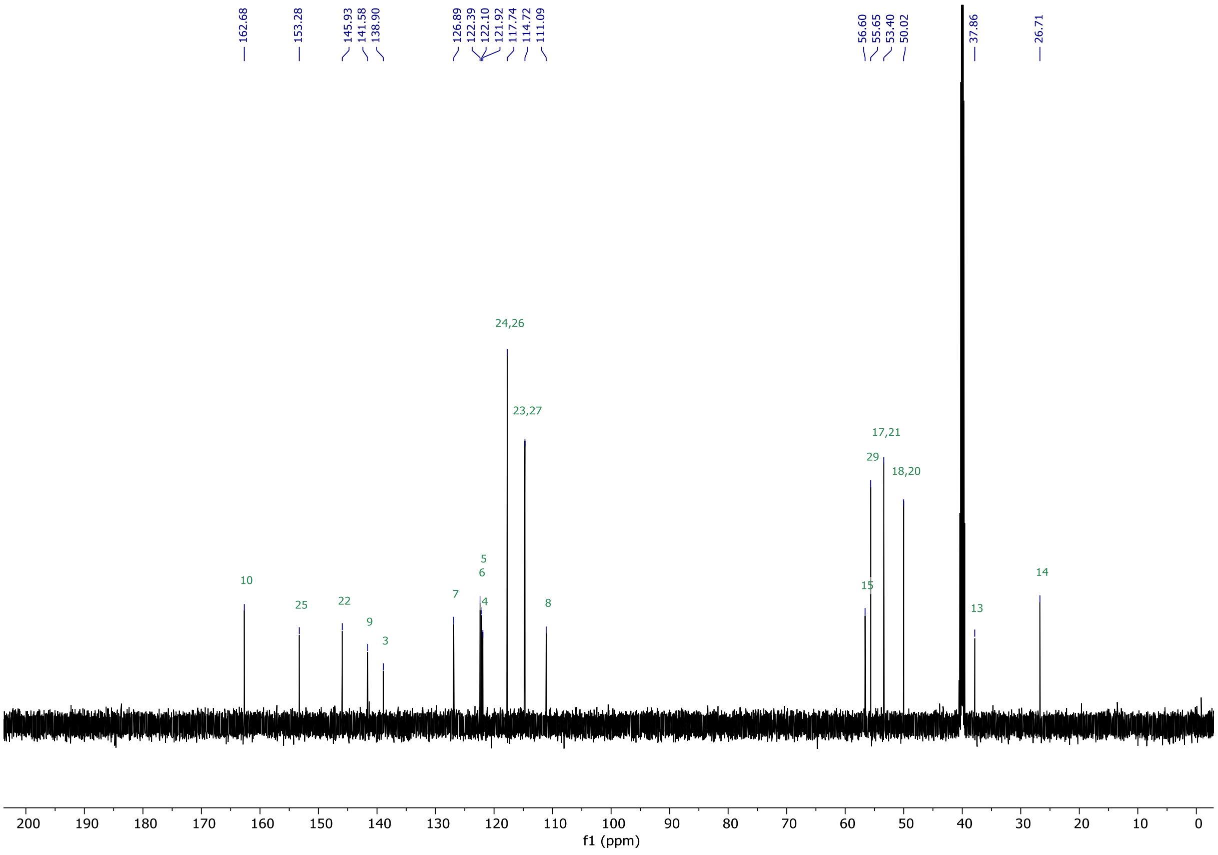

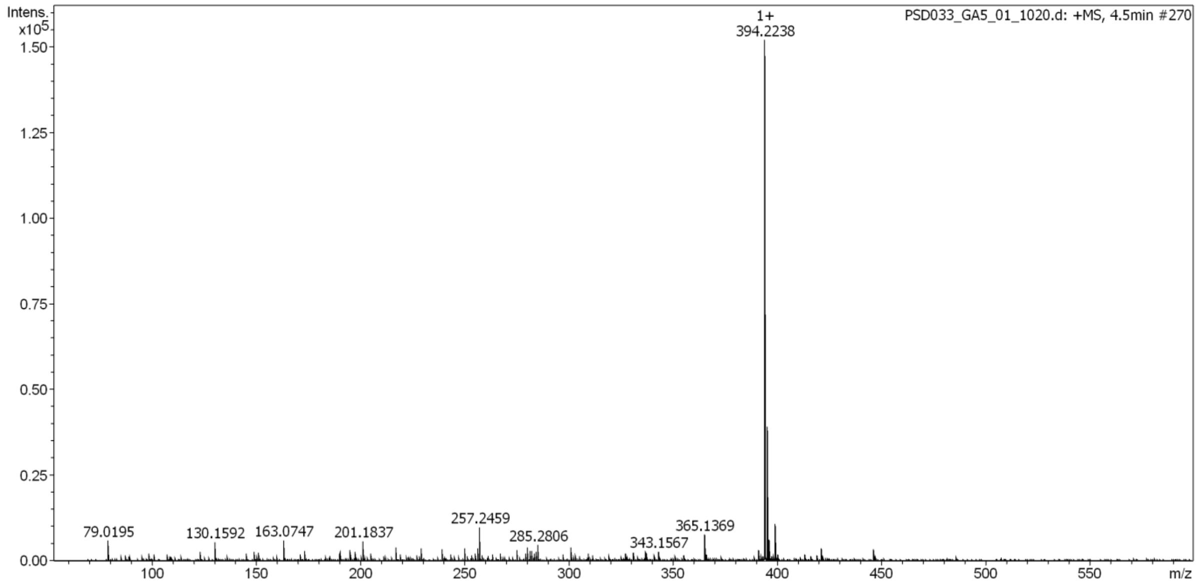
Intens.
x10^5
PSD033_GA5_01_1020.d: +MS, 4.5min #270
1+
394.2238
79.0195
130.1592
163.0747
201.1837
257.2459
285.2806
343.1567
365.1369
m/z

***N*-(3-(4-(pyridin-2-yl)piperazin-1-yl)propyl)-1*H*-indazole-3-carboxamide (5)**

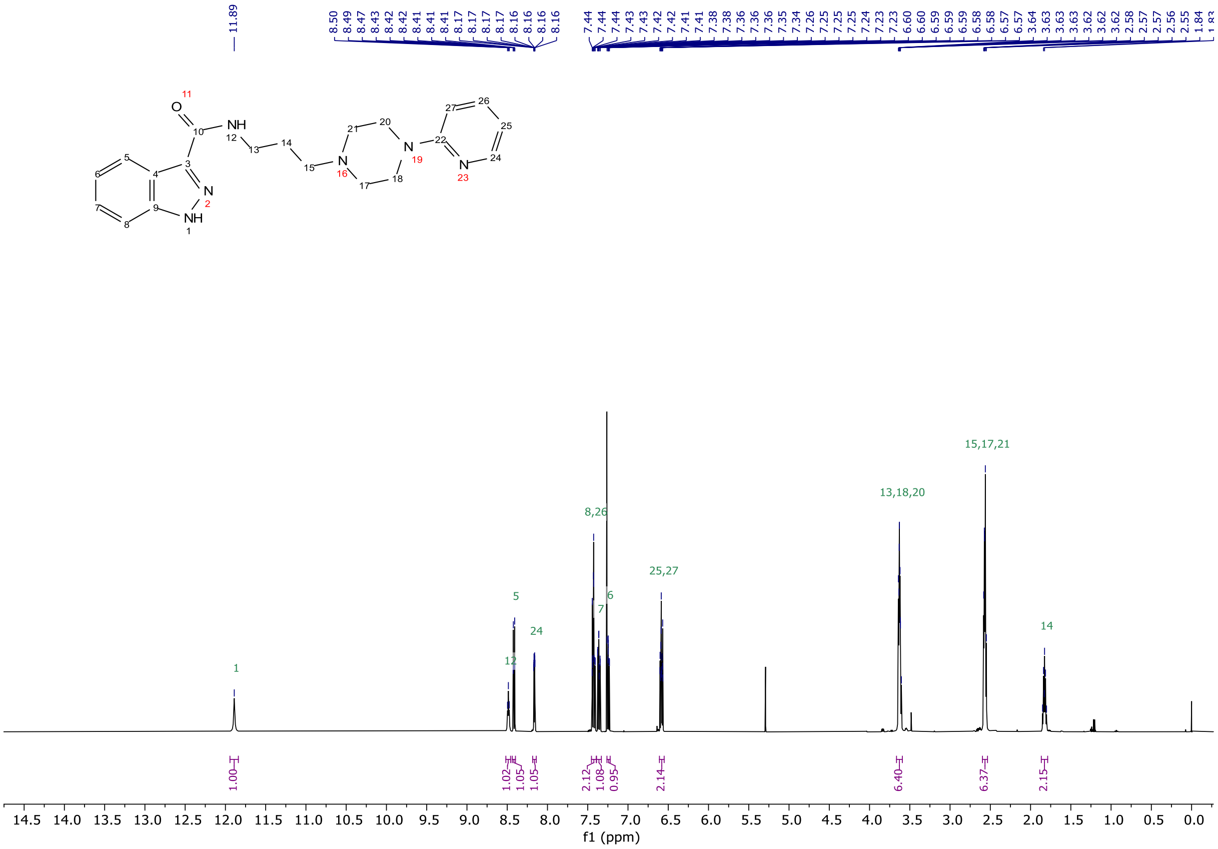

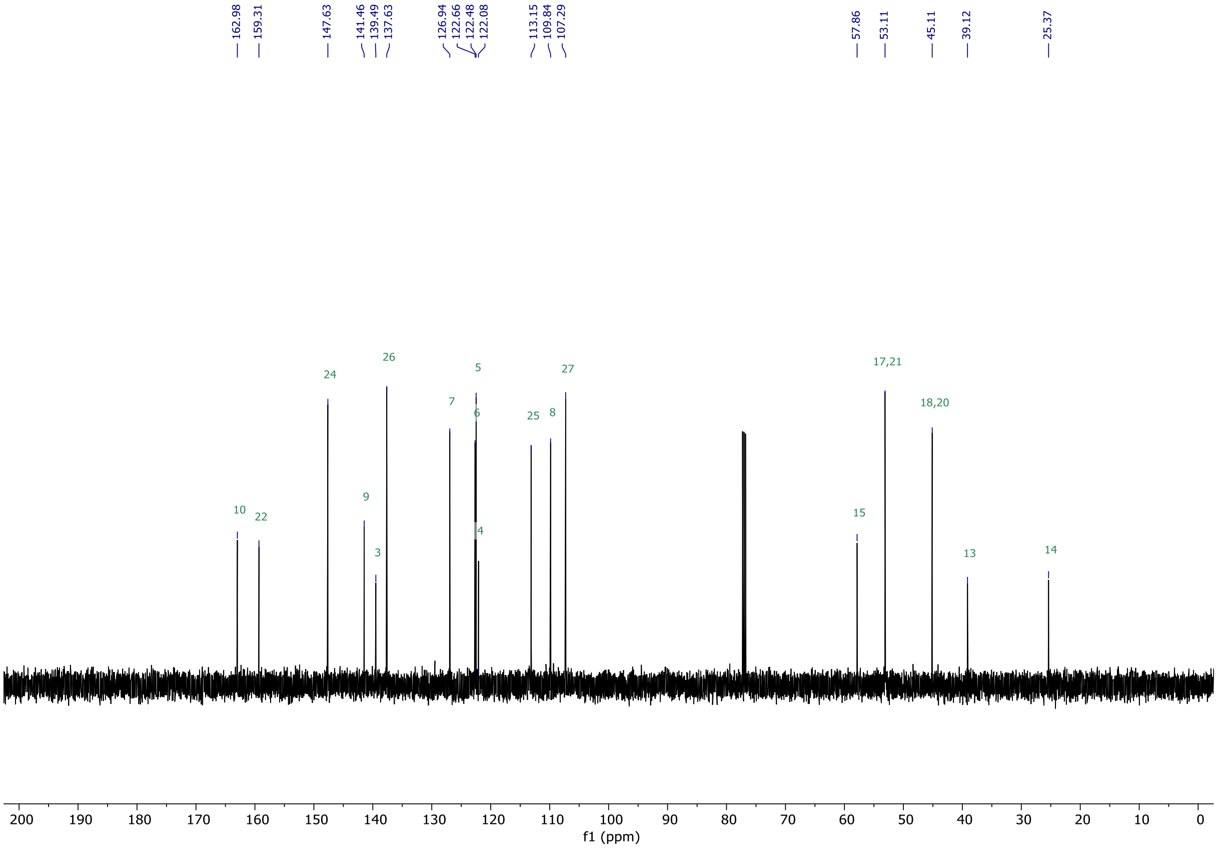

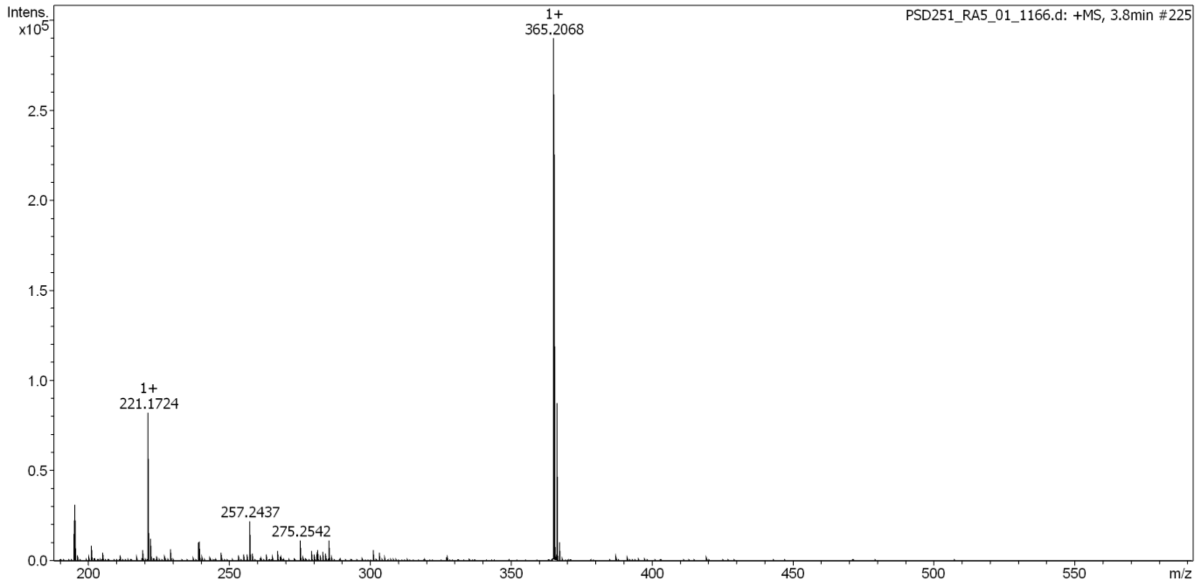
Intens.
x10^5
PSD251_RA5_01_1166.d: +MS, 3.8min #225
1+
365.2068
1+
221.1724
257.2437
275.2542
2.5
2.0
1.5
1.0
0.5
0.0
200
250
300
350
400
450
500
550
m/z

N-(3-(4-(pyridin-3-yl)piperazin-1-yl)propyl)-1*H*-indazole-3-carboxamide (6)

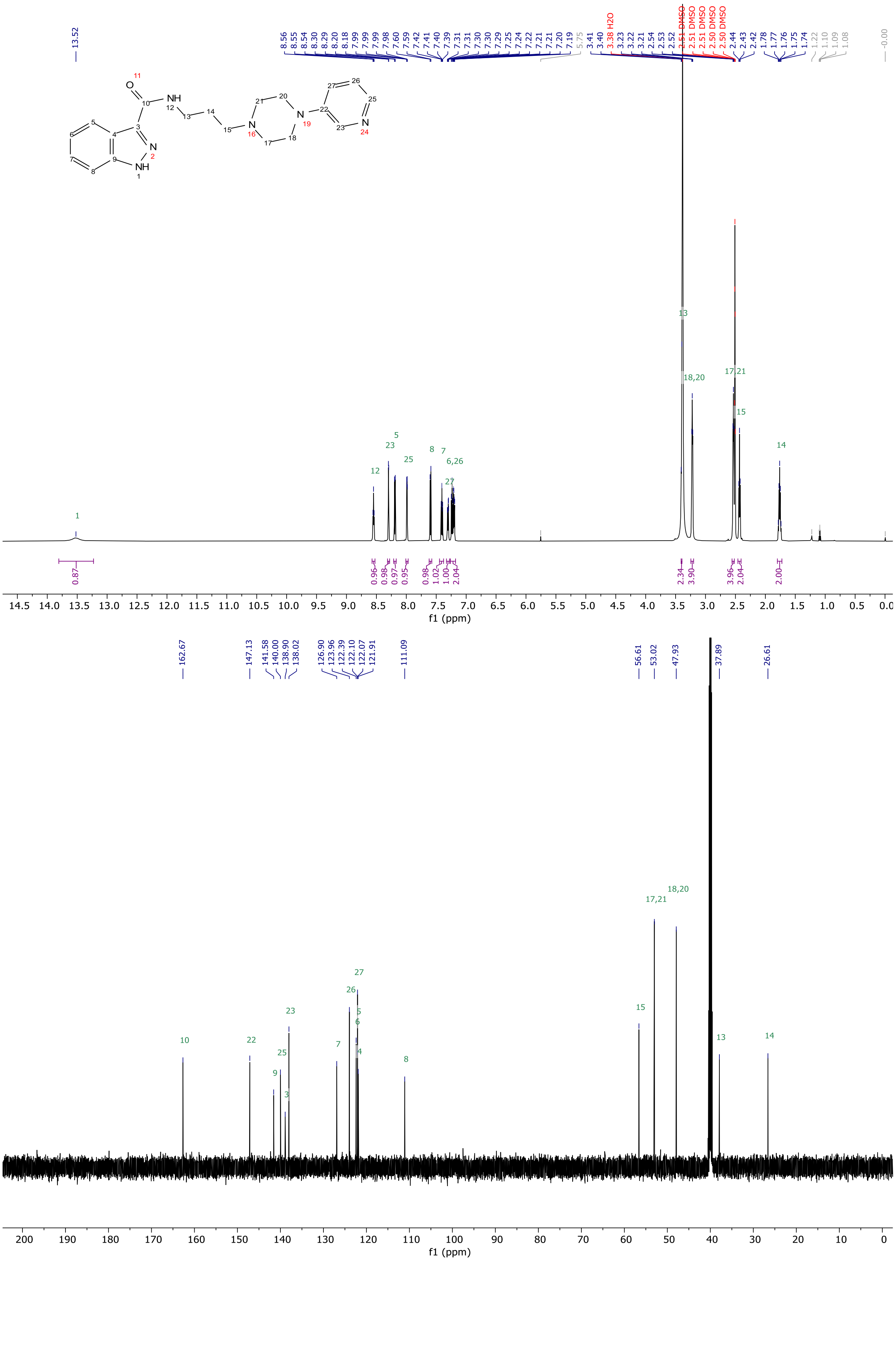

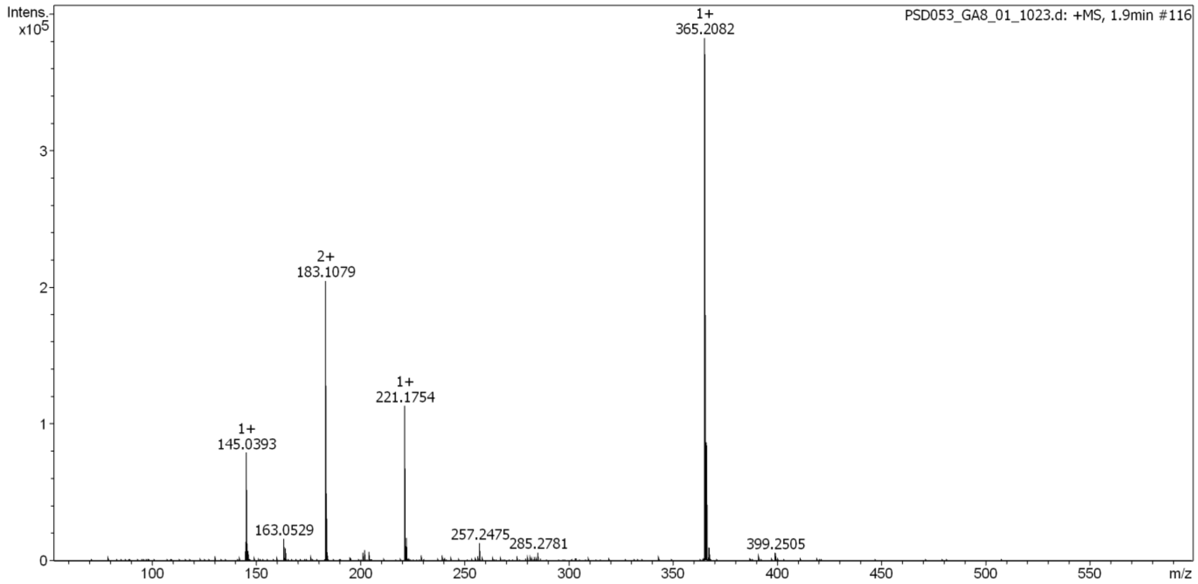

Intens.
x10^5
PSD053_GA8_01_1023.d: +MS, 1.9min #116
1+
365.2082
2+
183.1079
1+
221.1754
1+
145.0393
163.0529
257.2475
285.2781
399.2505
3
2
1
0
100
150
200
250
300
350
400
450
500
550
m/z

N-(3-(4-(pyridin-4-yl)piperazin-1-yl)propyl)-1*H*-indazole-3-carboxamide (7)

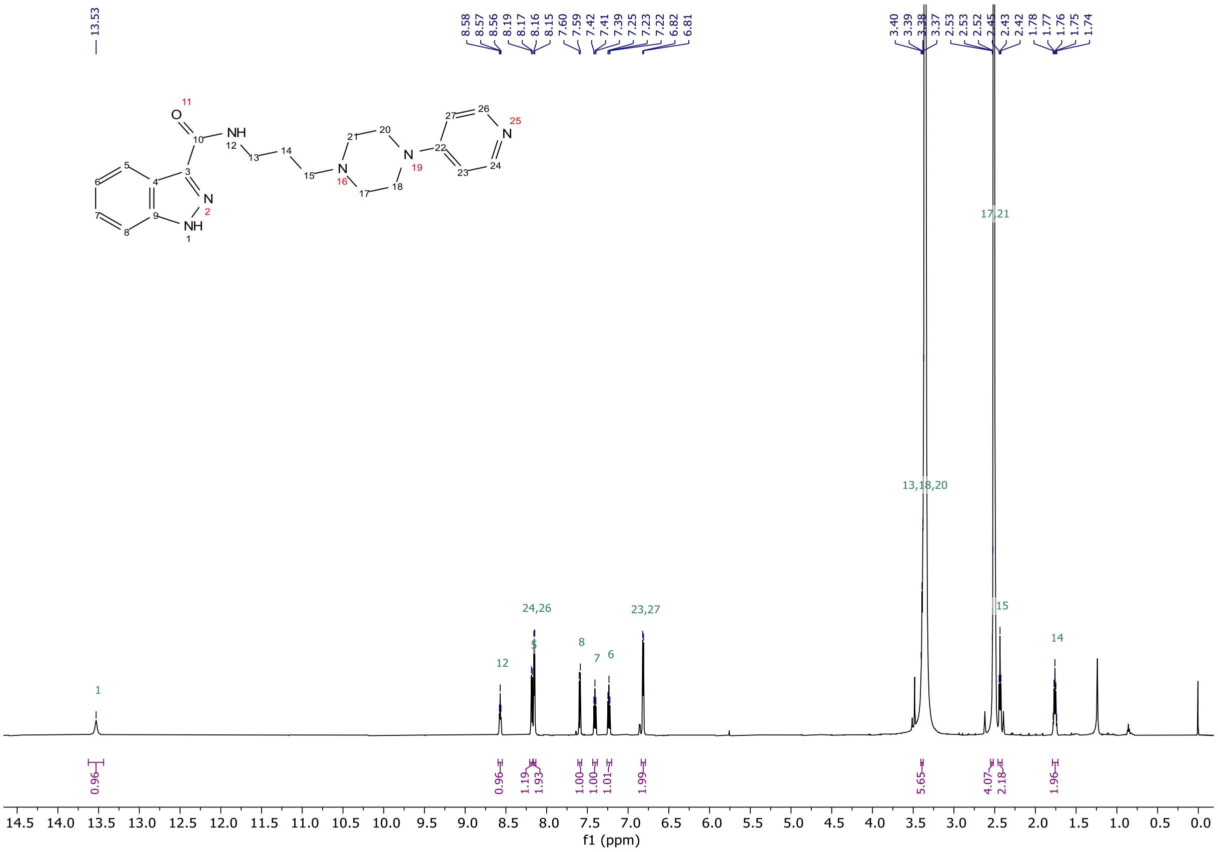

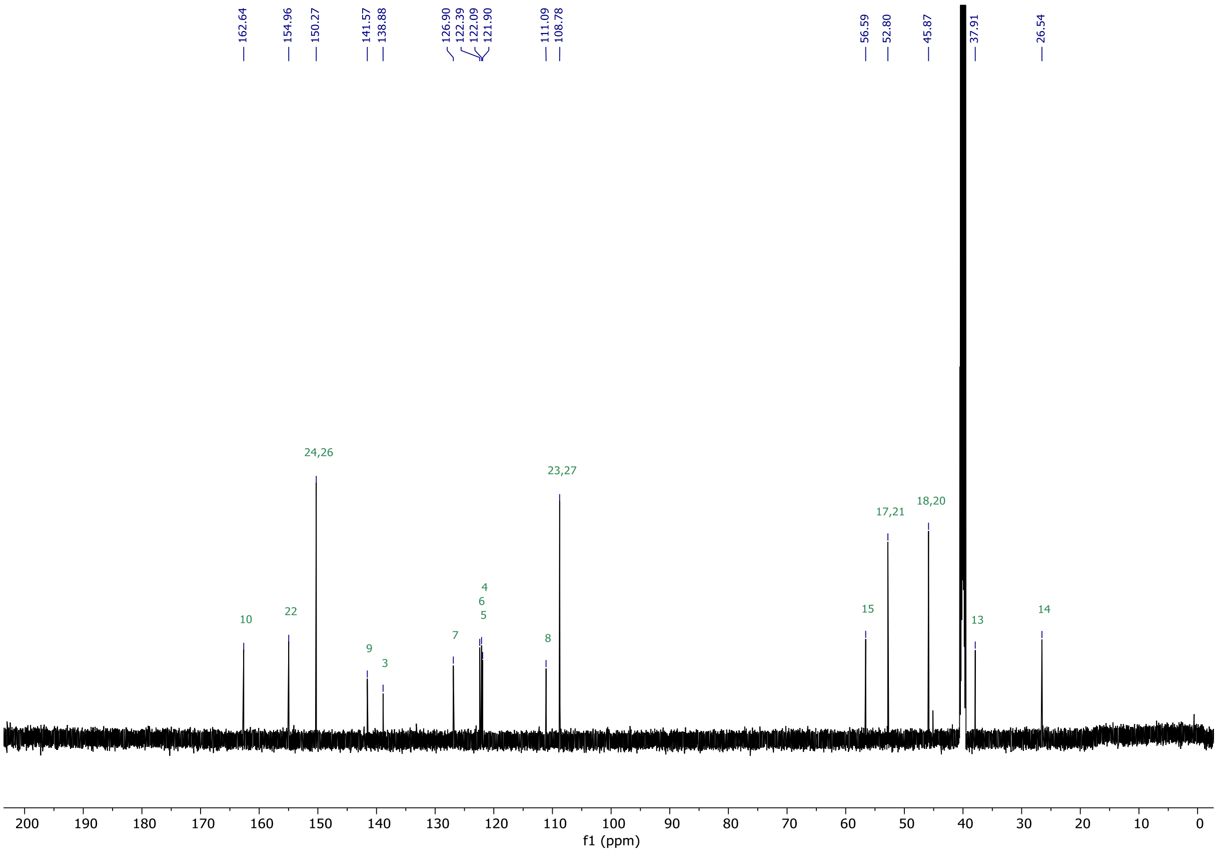

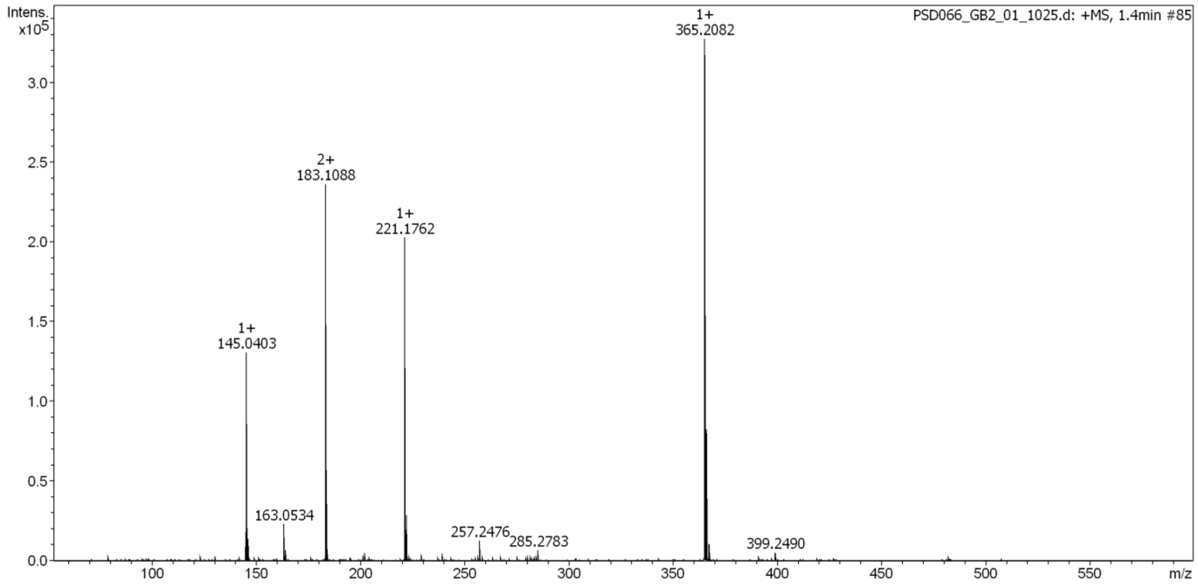

Intens.
x10^5
PSD066_GB2_01_1025.d: +MS, 1.4min #85
1+
365.2082
2+
183.1088
1+
221.1762
1+
145.0403
163.0534
257.2476
285.2783
399.2490
3.0
2.5
2.0
1.5
1.0
0.5
0.0
100
150
200
250
300
350
400
450
500
550
m/z

N-(3-(4-(pyrimidin-2-yl)piperazin-1-yl)propyl)-1*H*-indazole-3-carboxamide (8)

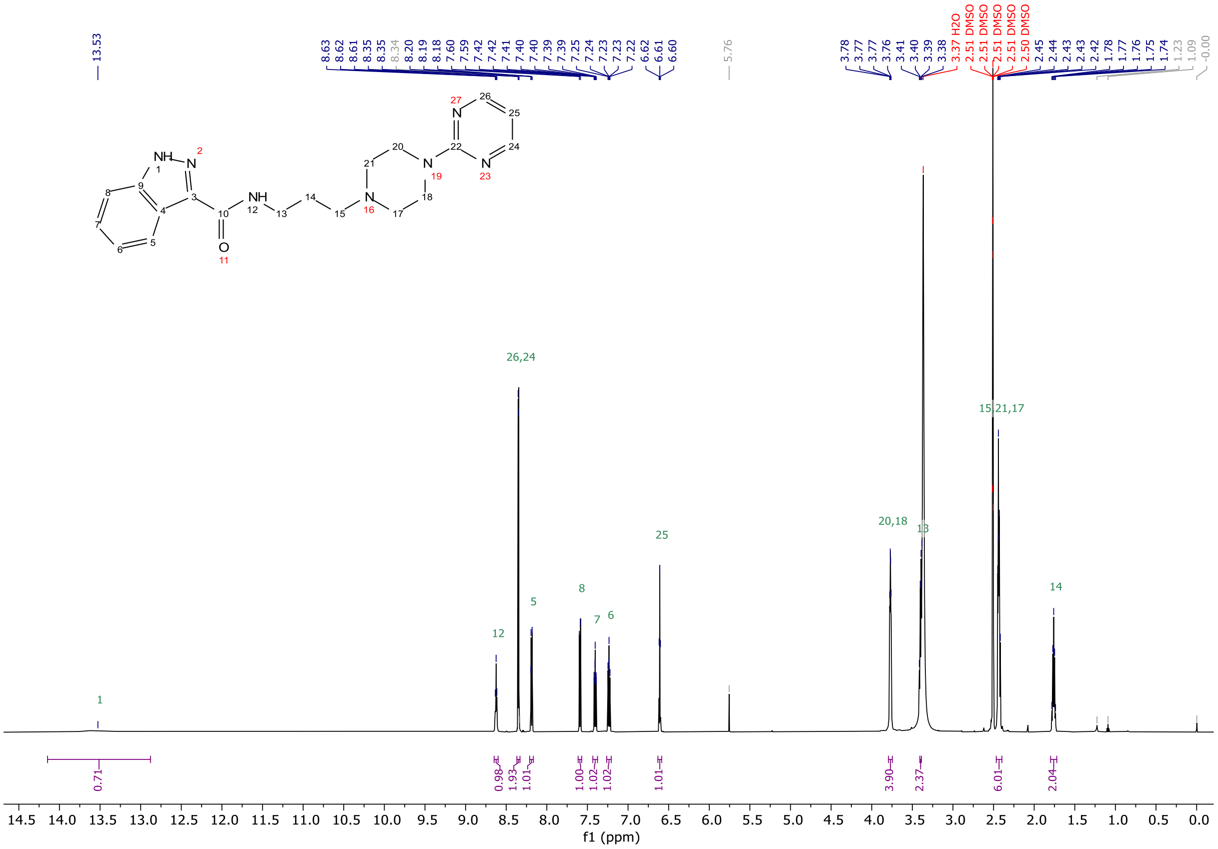

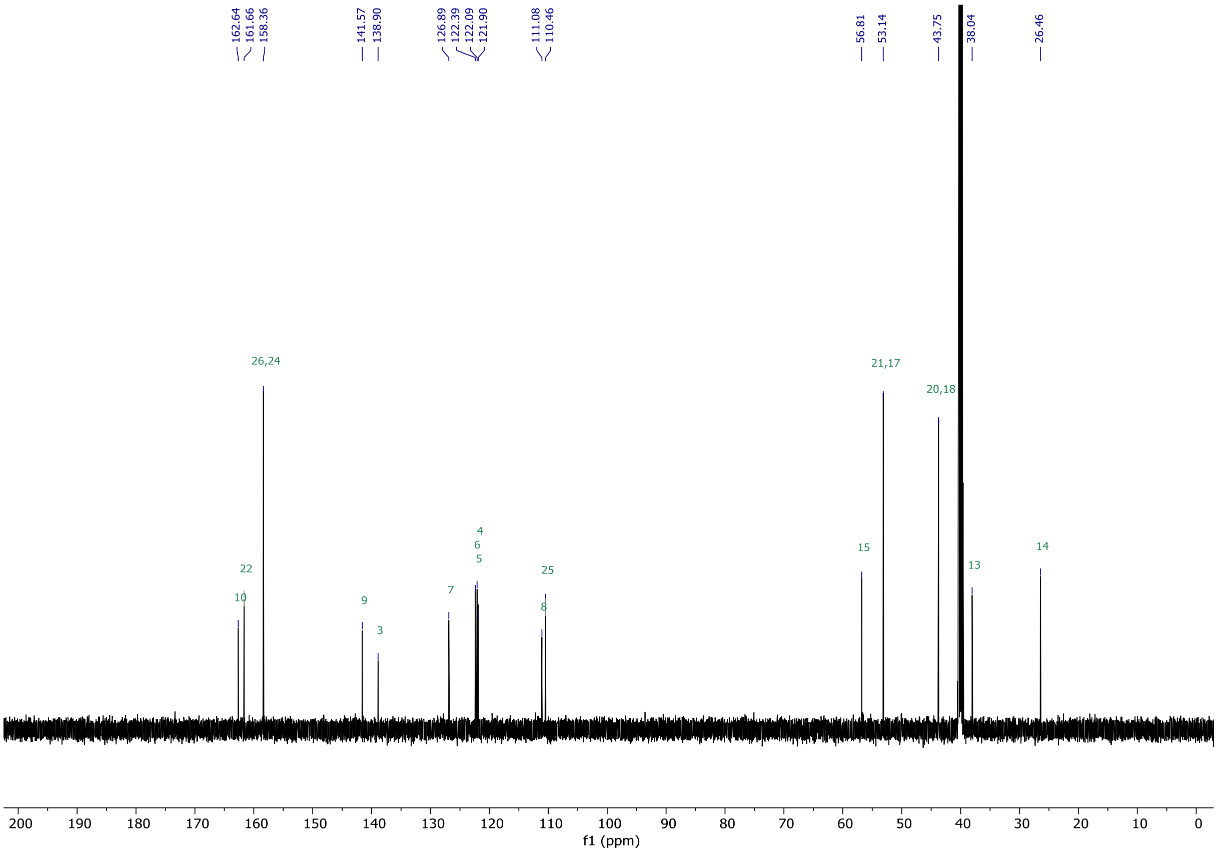

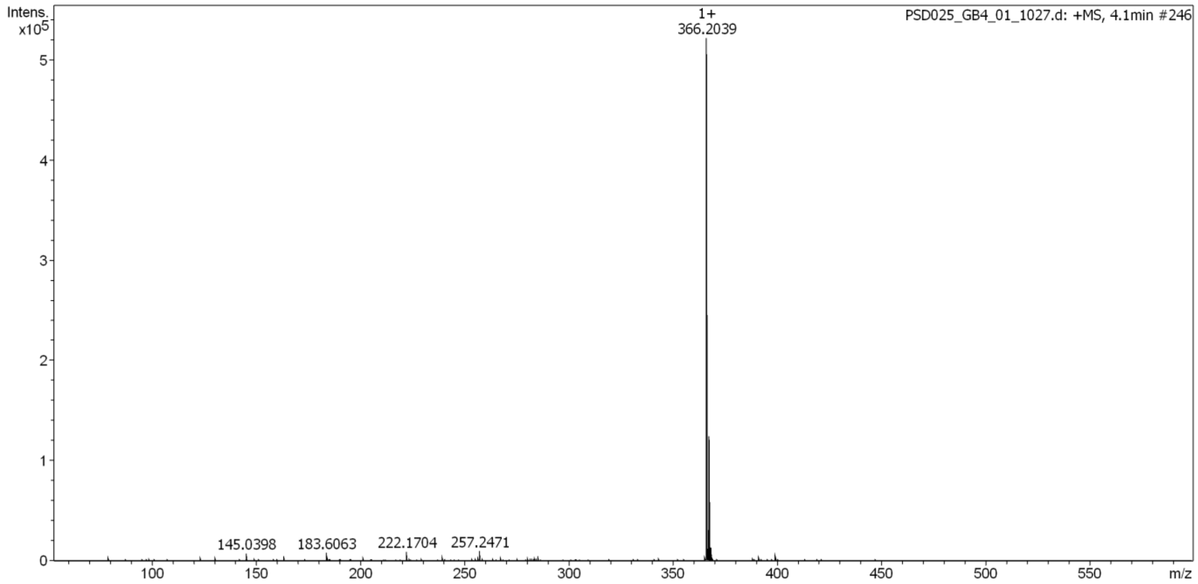

Intens.
x10^5
PSD025_GB4_01_1027.d: +MS, 4.1min #246
1+
366.2039
145.0398
183.6063
222.1704
257.2471
m/z

N-(3-(4-(2-fluorophenyl)piperazin-1-yl)propyl)-1*H*-indazole-3-carboxamide (9)

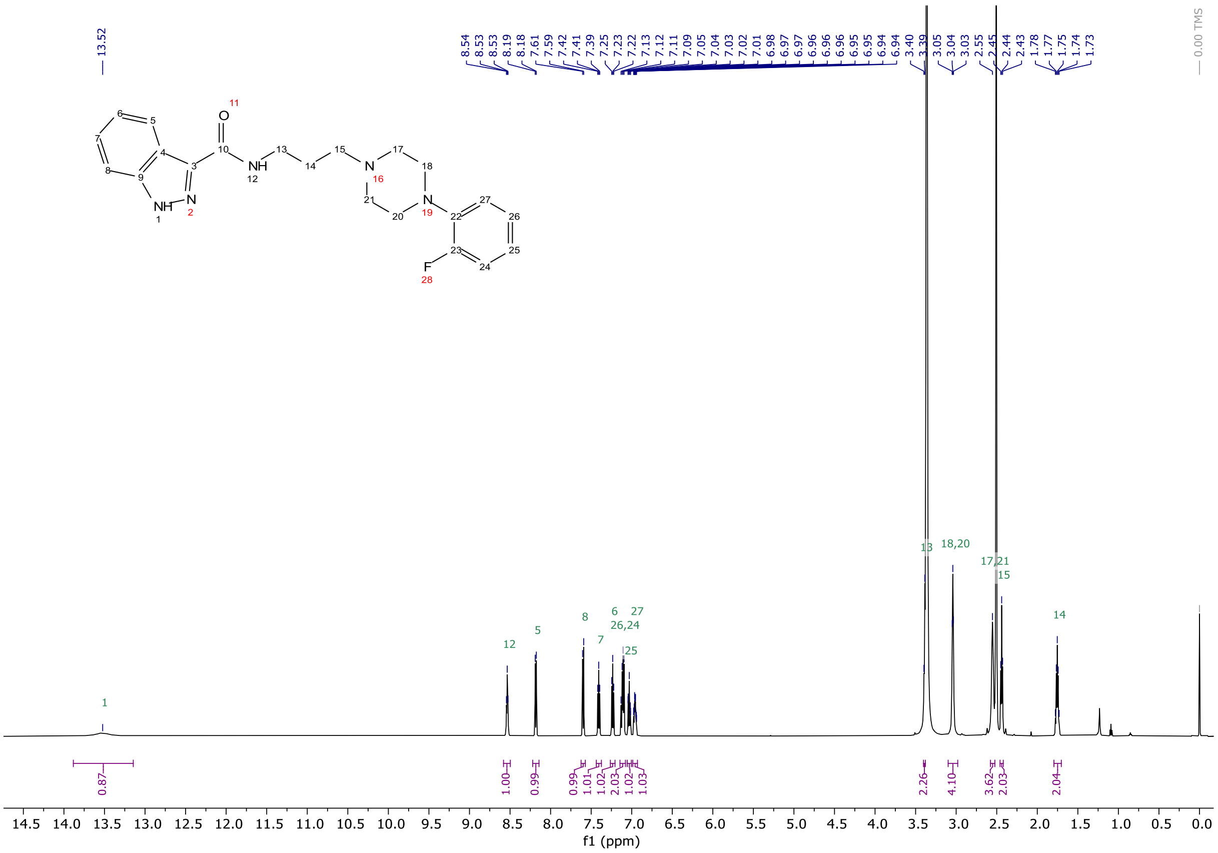

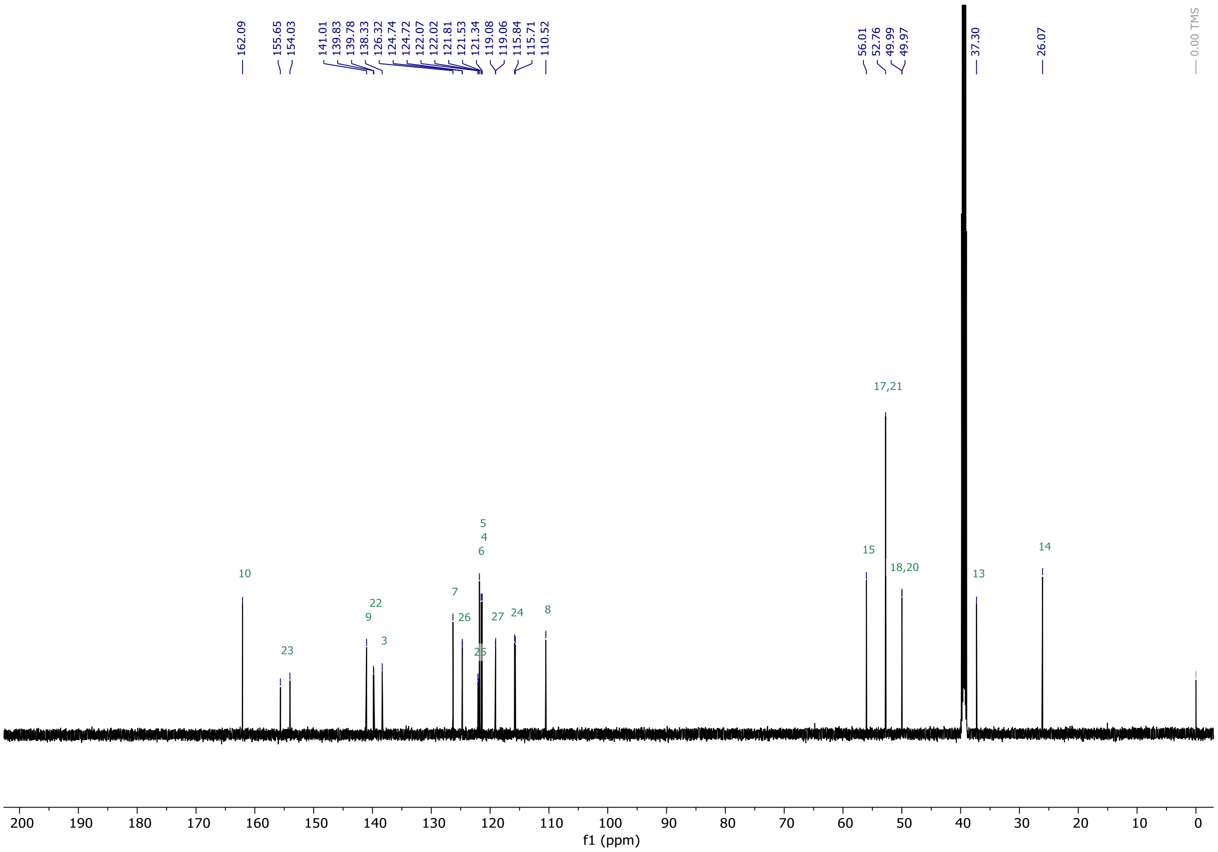

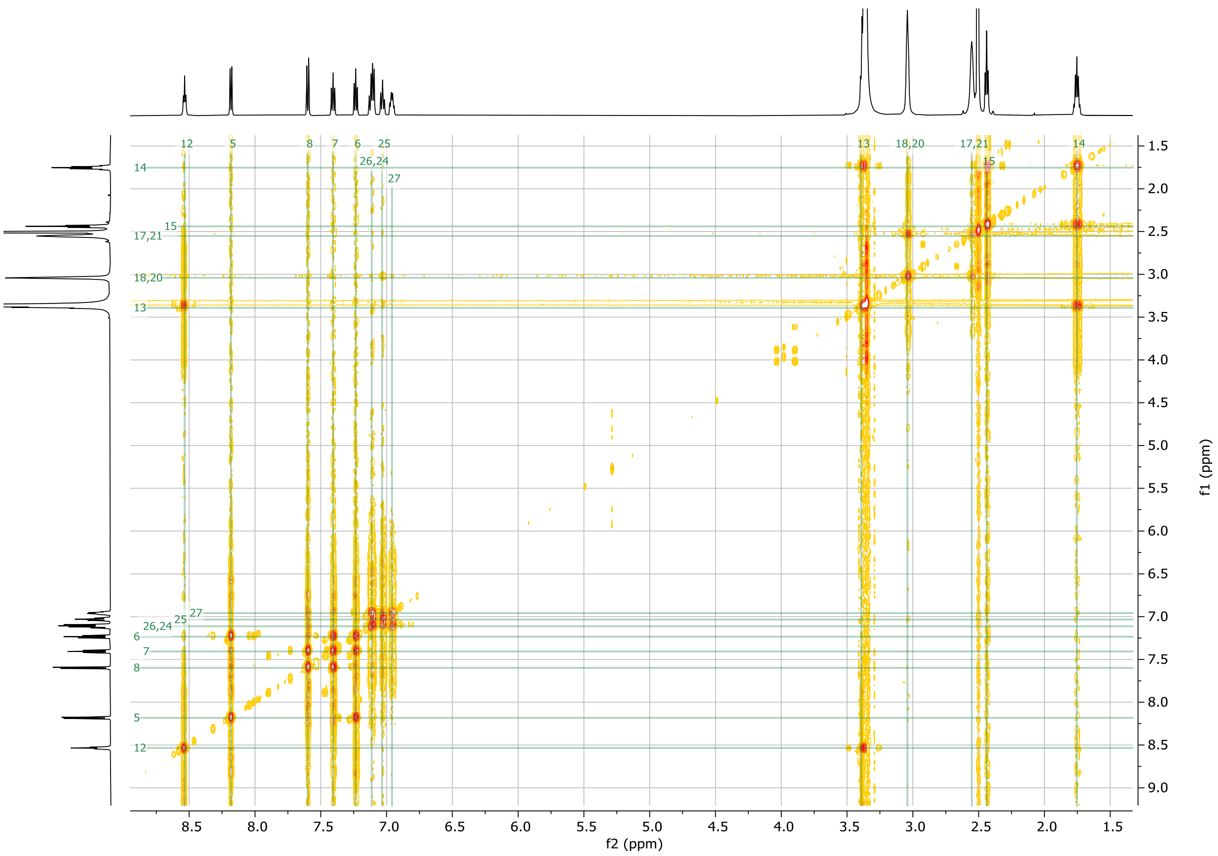

12
5
8
7
6
25
26,24
27
13
18,20
17,21
15
14
f2 (ppm)
f1 (ppm)

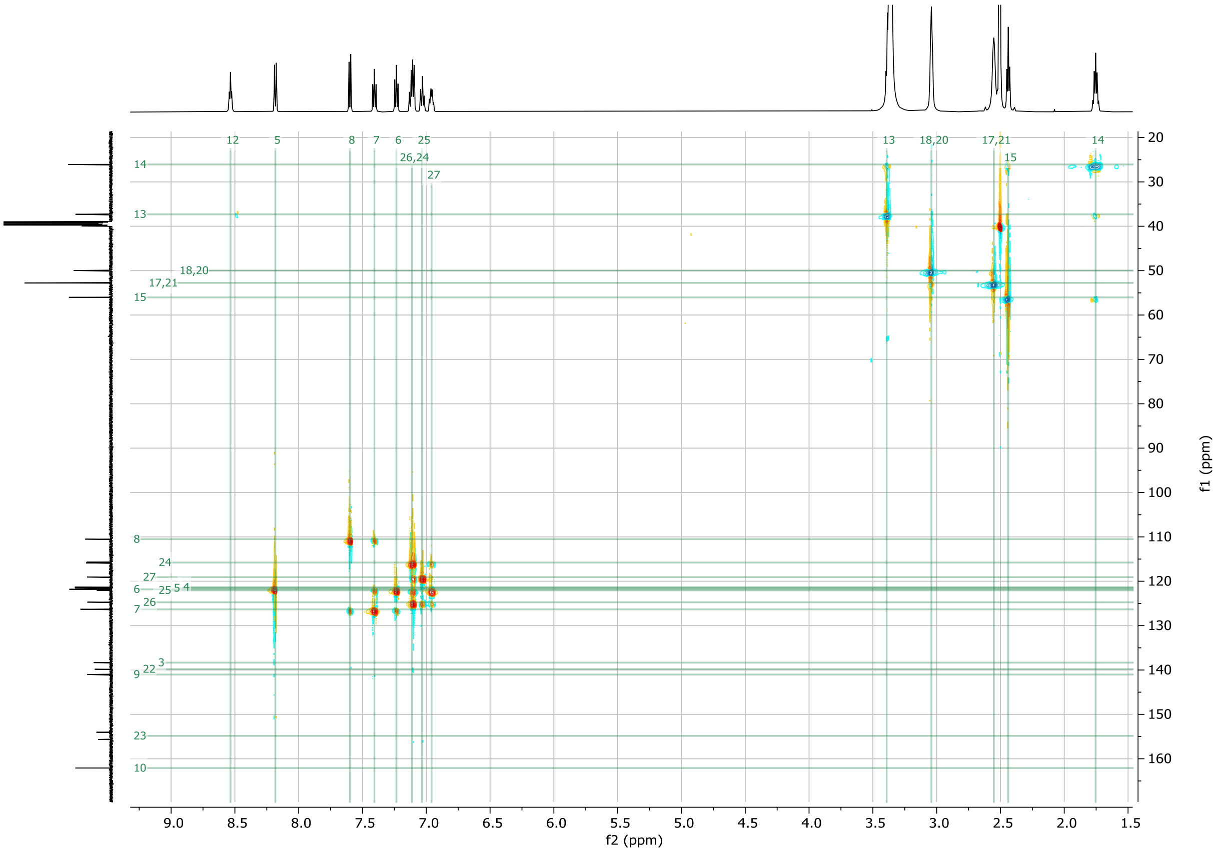

12
5
8
7
6
25
26,24
27
13
18,20
17,21
15
14
24
5 4
26
3
9
22
23
10
f2 (ppm)
f1 (ppm)

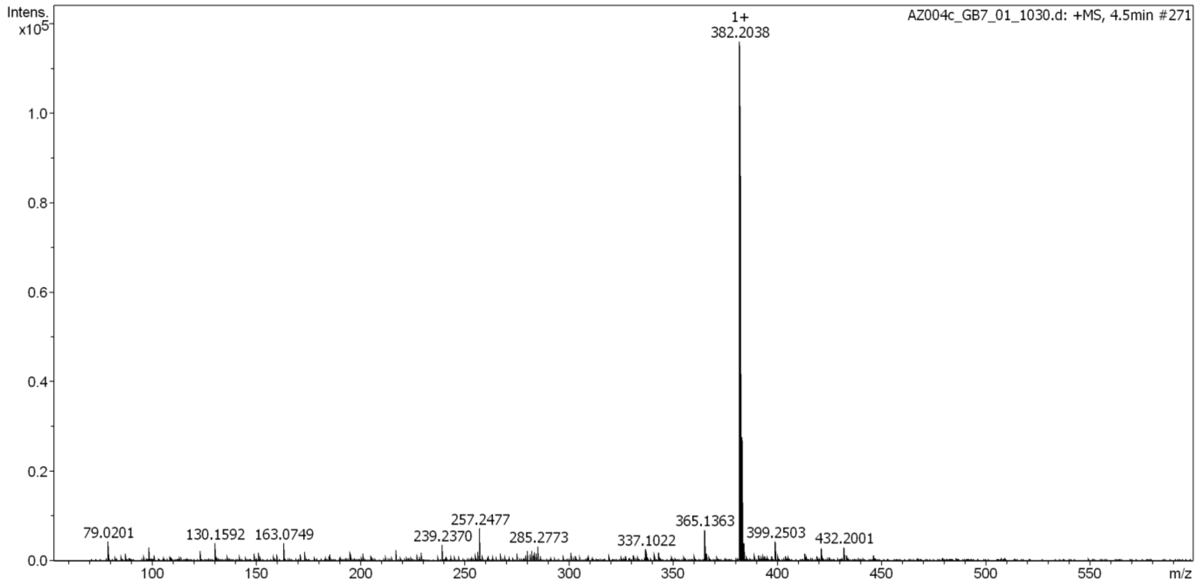

Intens.
x10^5
AZ004c_GB7_01_1030.d: +MS, 4.5min #271
1+
382.2038
1.0
0.8
0.6
0.4
0.2
0.0
79.0201
130.1592
163.0749
239.2370
257.2477
285.2773
337.1022
365.1363
399.2503
432.2001
100
150
200
250
300
350
400
450
500
550
m/z

N-(3-(4-(3-fluorophenyl)piperazin-1-yl)propyl)-1*H*-indazole-3-carboxamide (10)

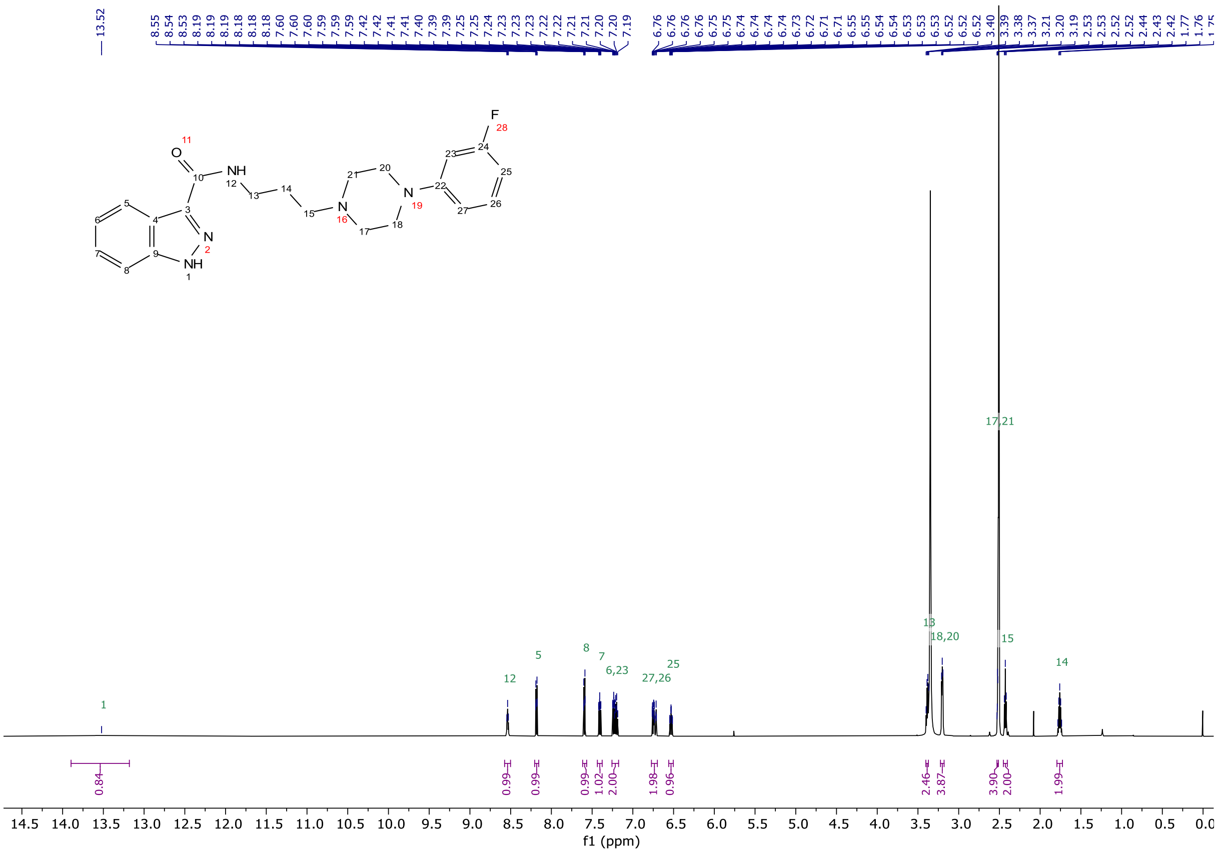

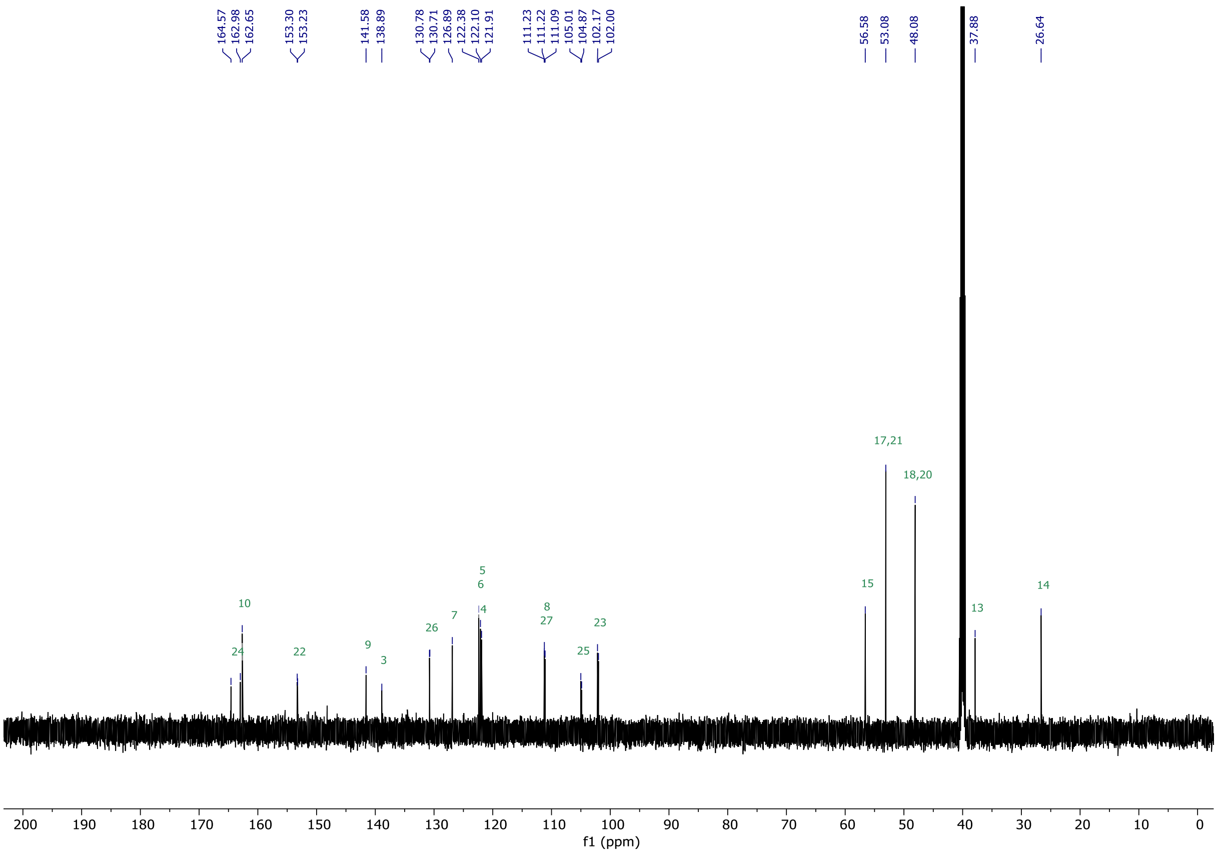

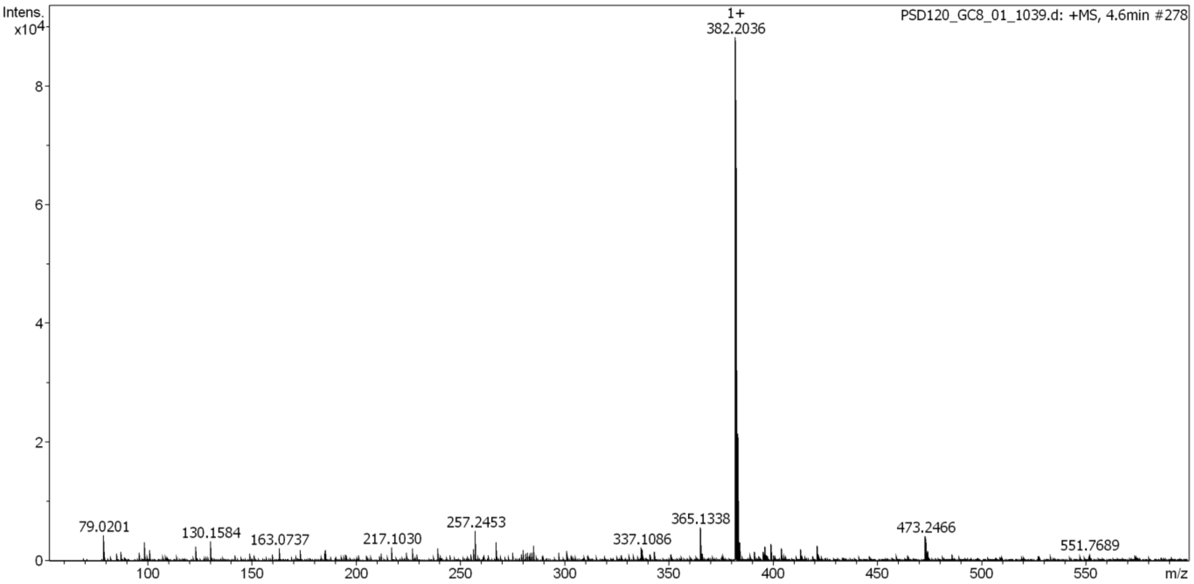

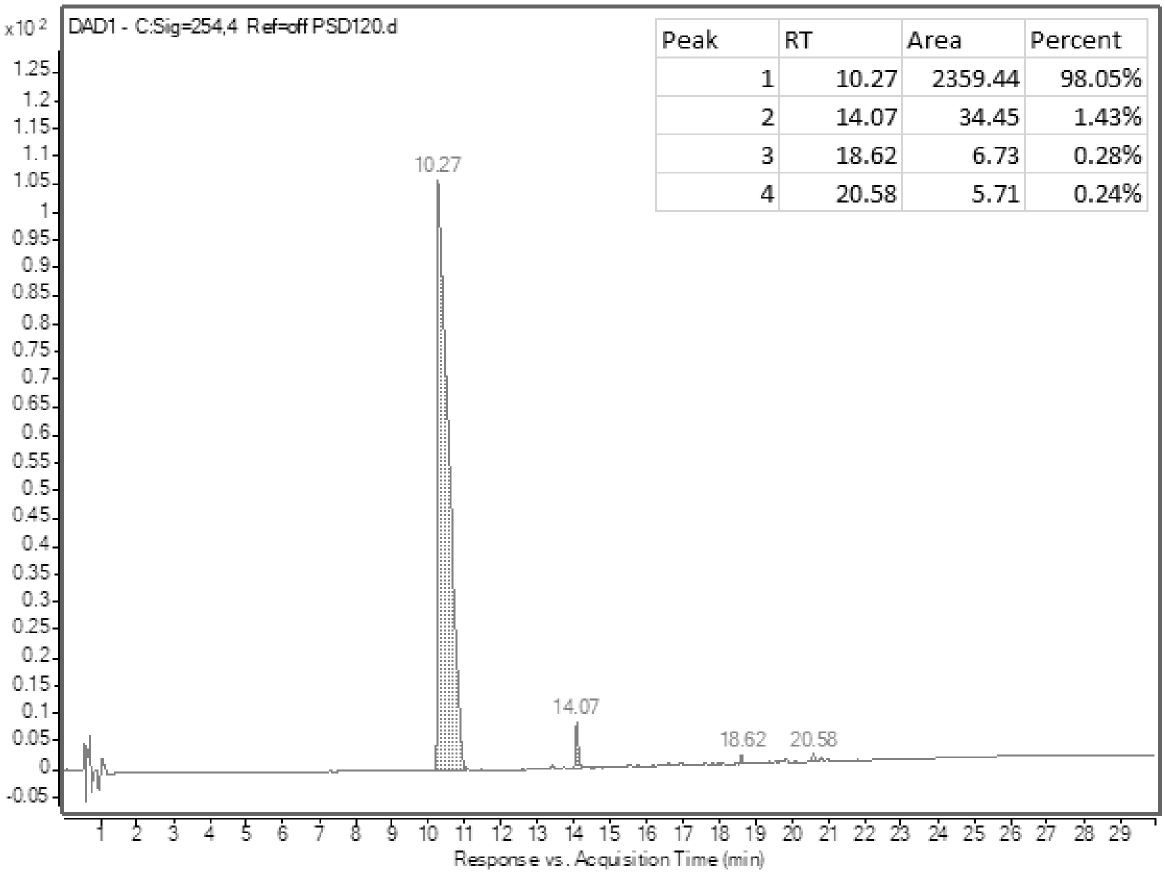

Peak	RT	Area	Percent
1	10.27	2359.44	98.05%
2	14.07	34.45	1.43%
3	18.62	6.73	0.28%
4	20.58	5.71	0.24%

N-(3-(4-(4-fluorophenyl)piperazin-1-yl)propyl)-1*H*-indazole-3-carboxamide (11)

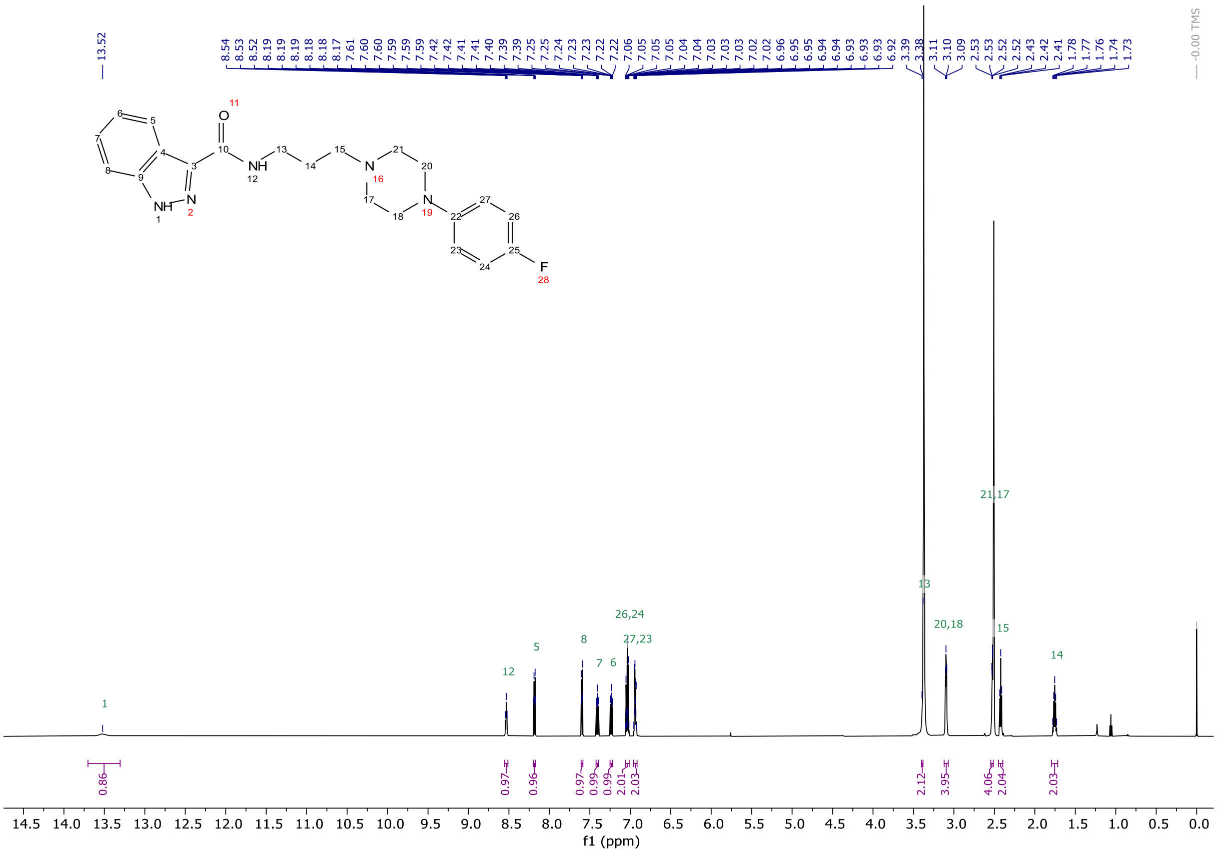

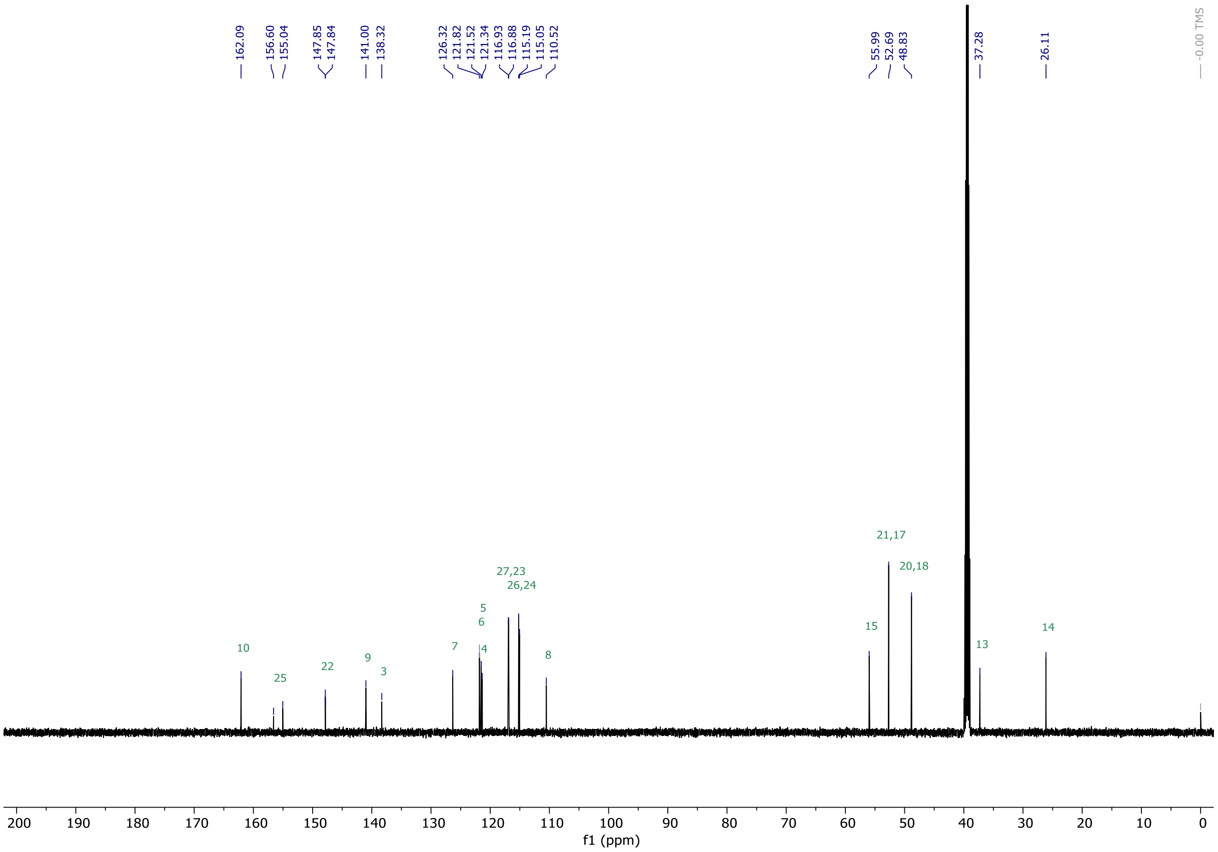

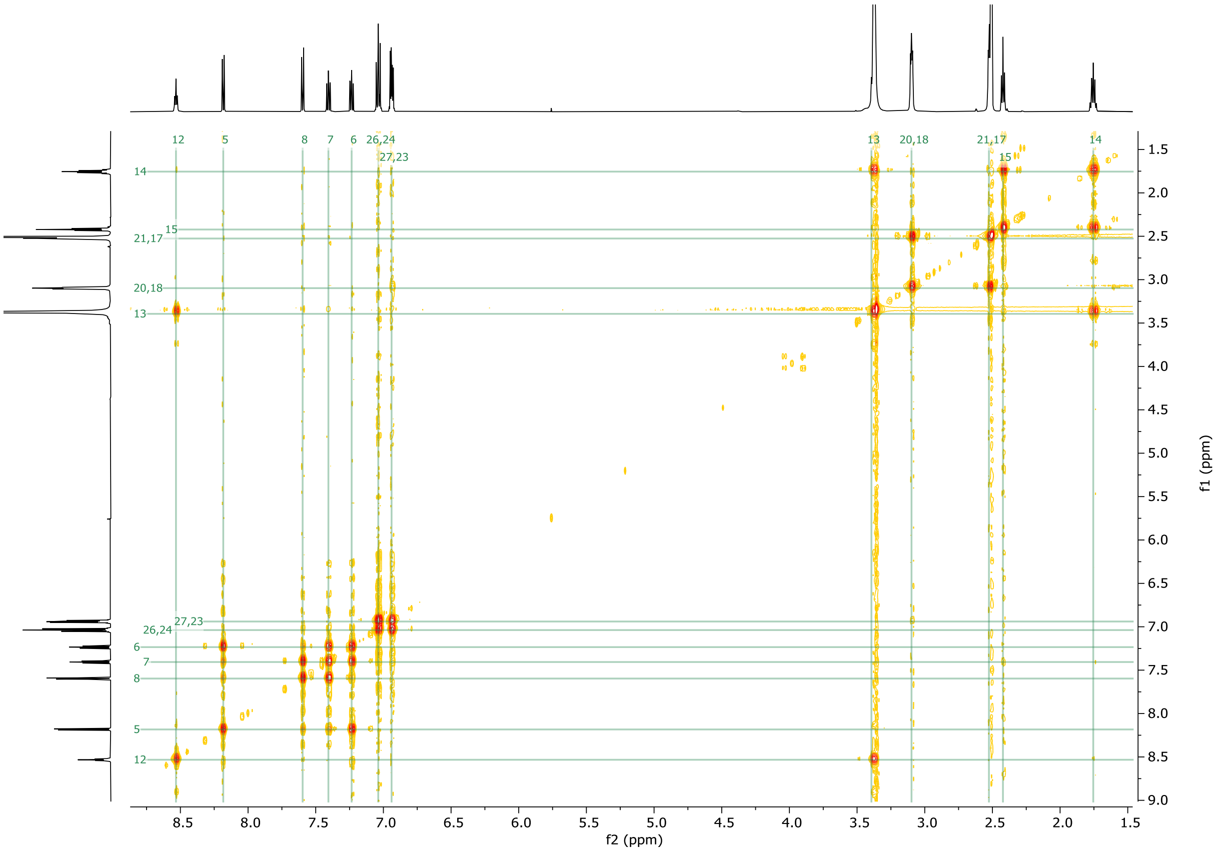
12
5
8
7
6
26,24
27,23
13
20,18
21,17
15
14
f2 (ppm)
f1 (ppm)

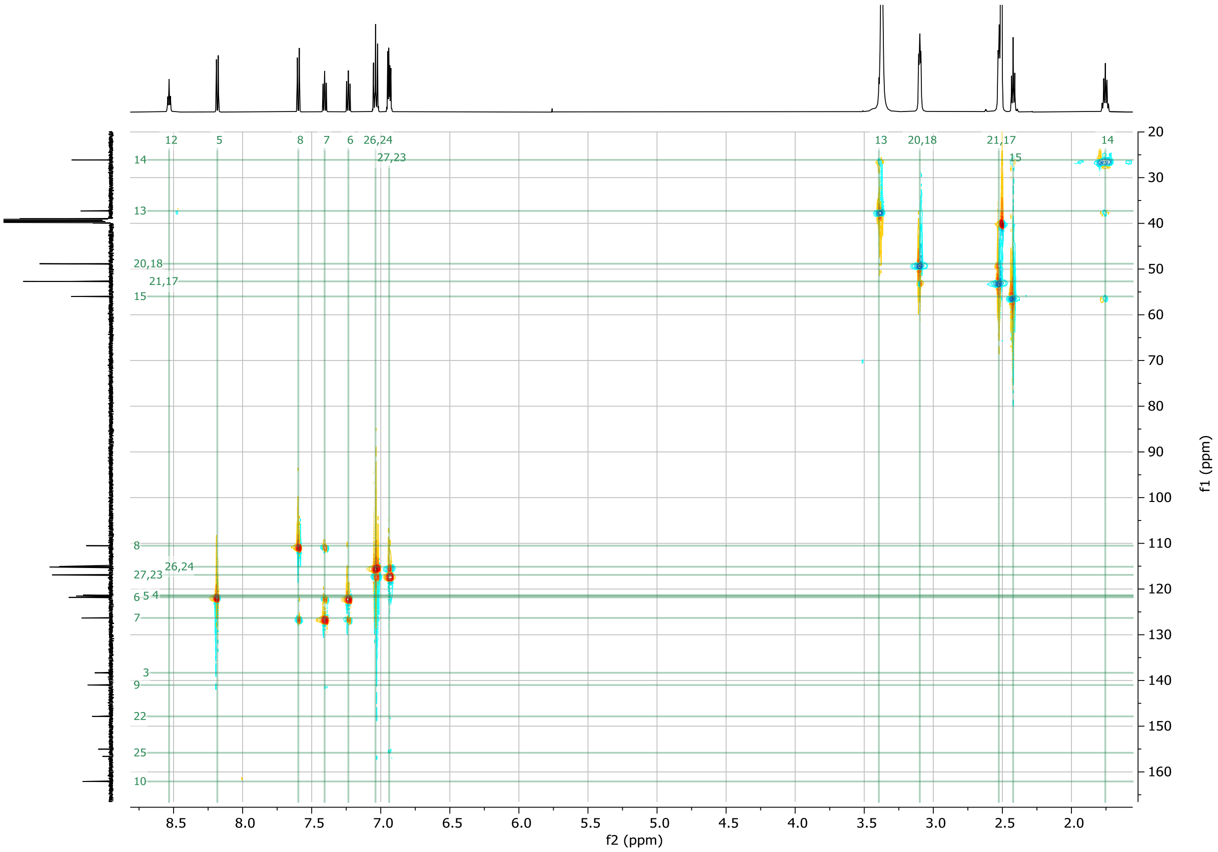
12
5
8
7
6
26,24
27,23
13
20,18
21,17
15
14
6 5 4
3
9
22
25
10
f2 (ppm)
f1 (ppm)

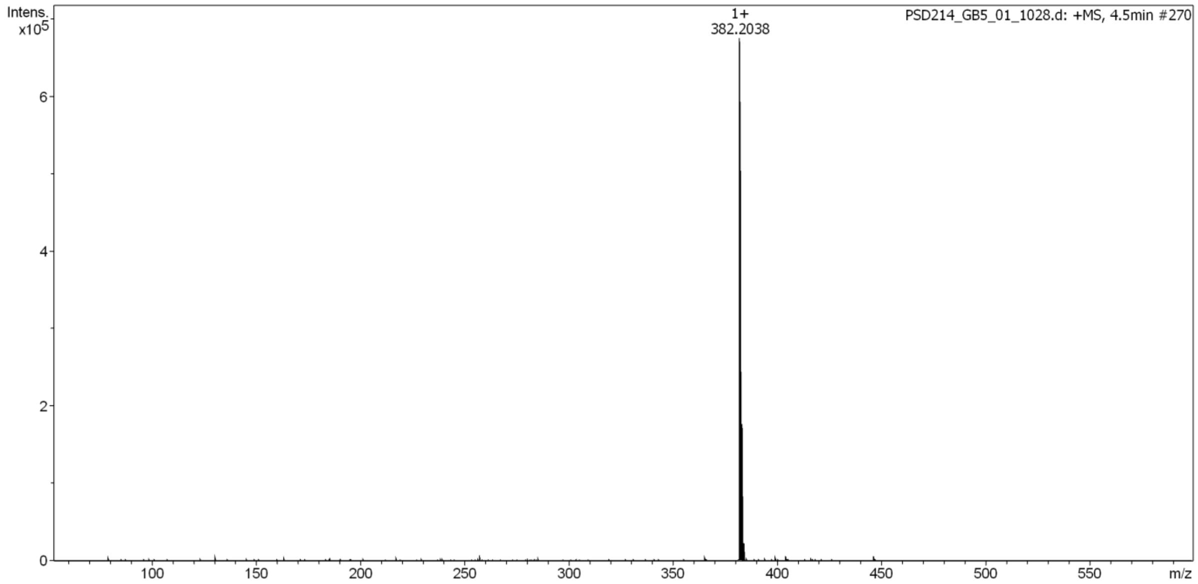

Intens.
x10^5
1+
382.2038
PSD214_GB5_01_1028.d: +MS, 4.5min #270
6
4
2
0
100
150
200
250
300
350
400
450
500
550
m/z

N-(3-(4-(2-(trifluoromethyl)phenyl)piperazin-1-yl)propyl)-1*H*-indazole-3-carboxamide (12)

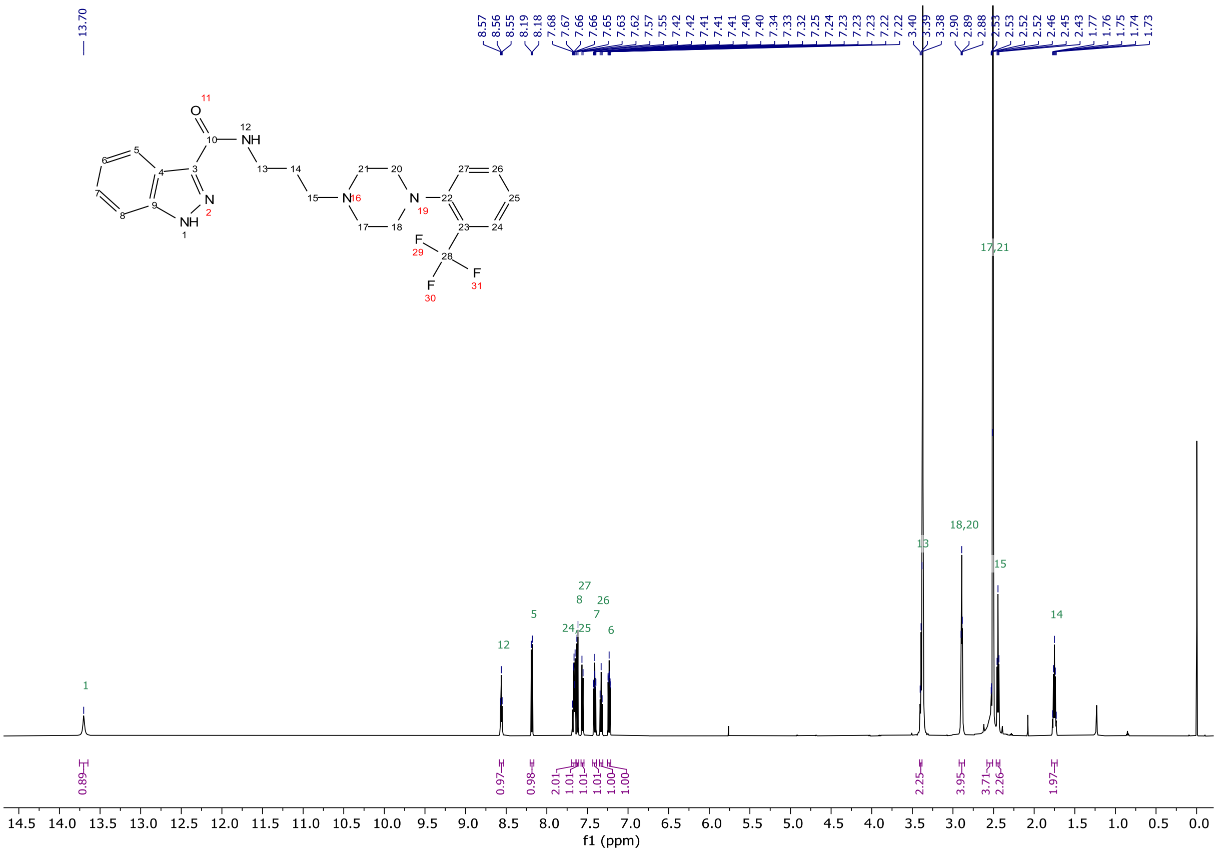

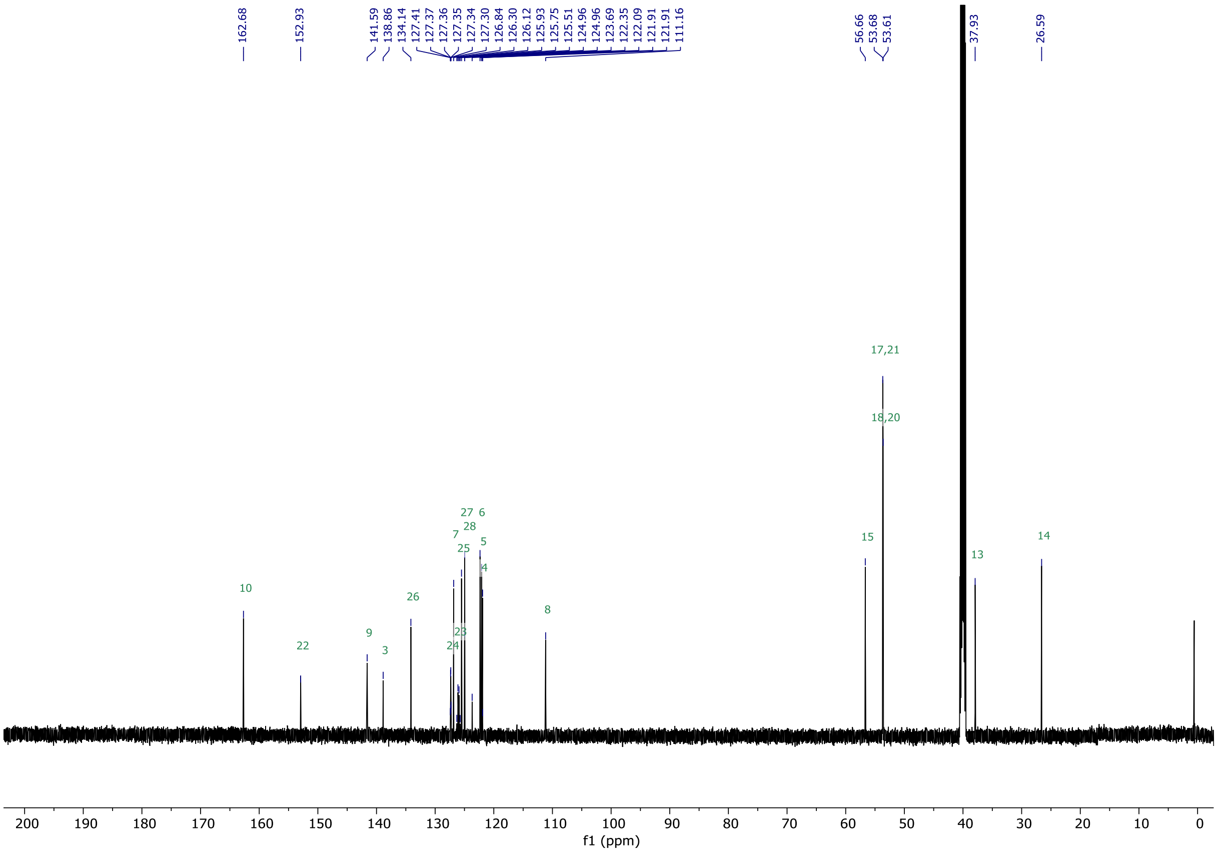

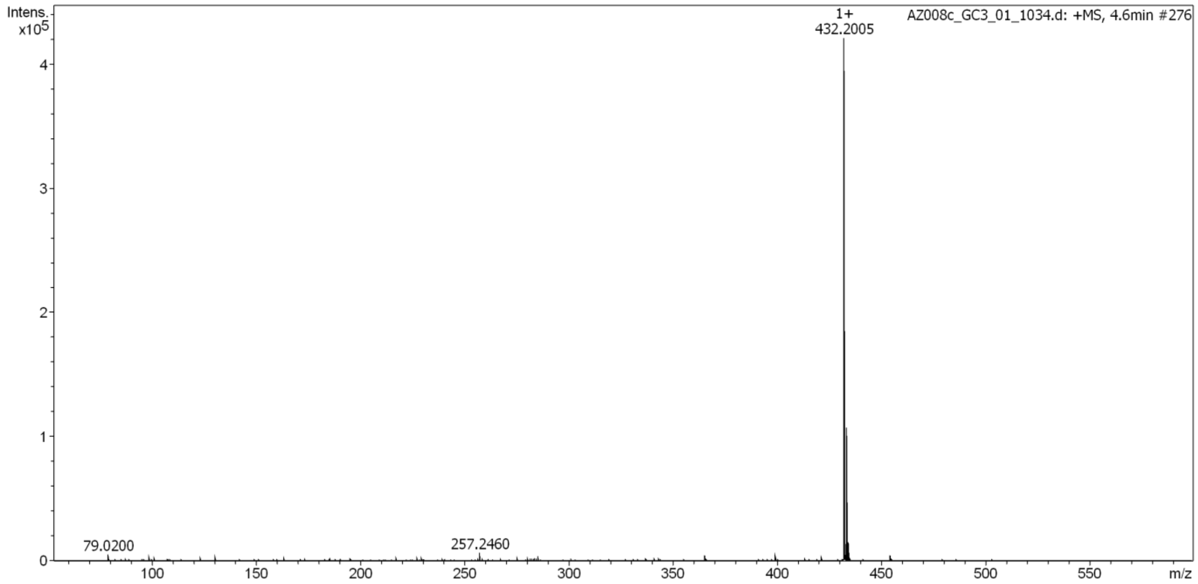
Intens.
x10^5
4
3
2
1
0
1+
432.2005
AZ008c_GC3_01_1034.d: +MS, 4.6min #276
79.0200
257.2460
100
150
200
250
300
350
400
450
500
550
m/z

N-(3-(4-(2-chlorophenyl)piperazin-1-yl)propyl)-1*H*-indazole-3-carboxamide (13)

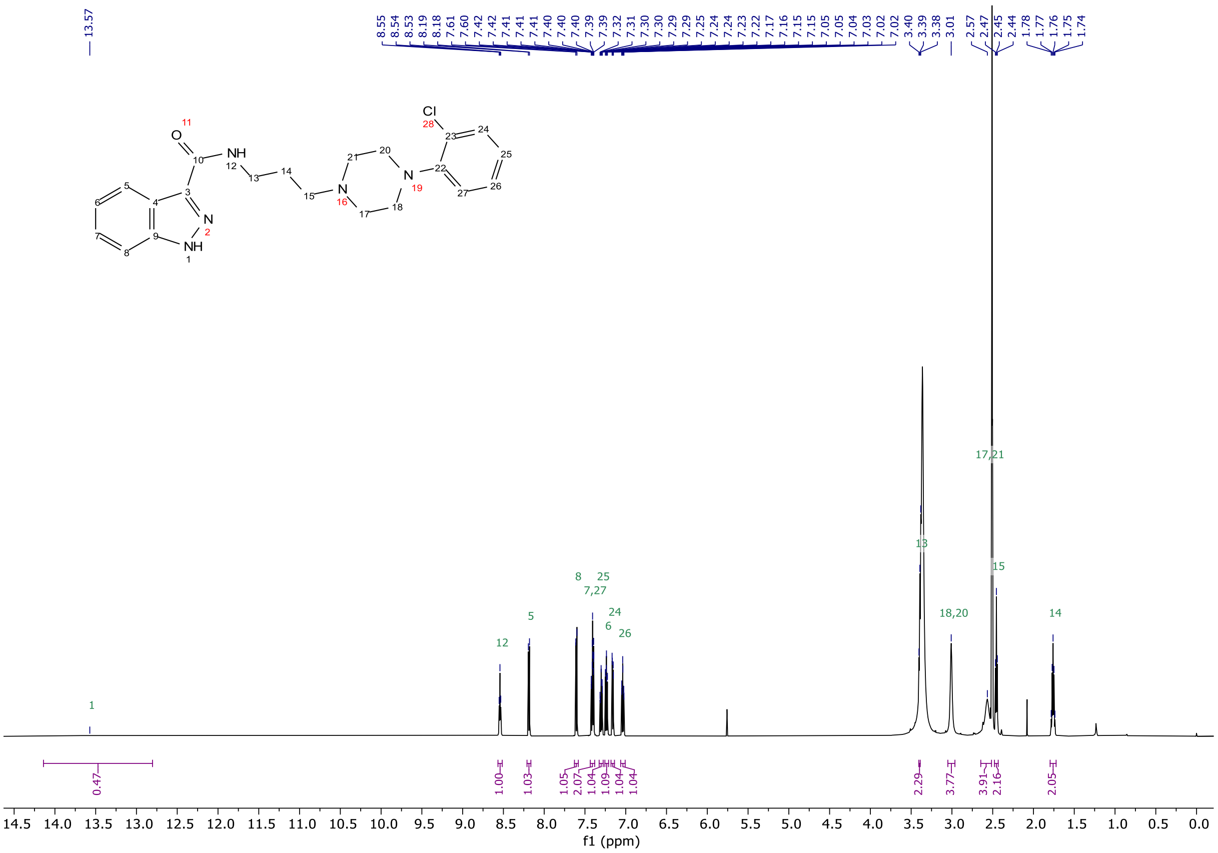

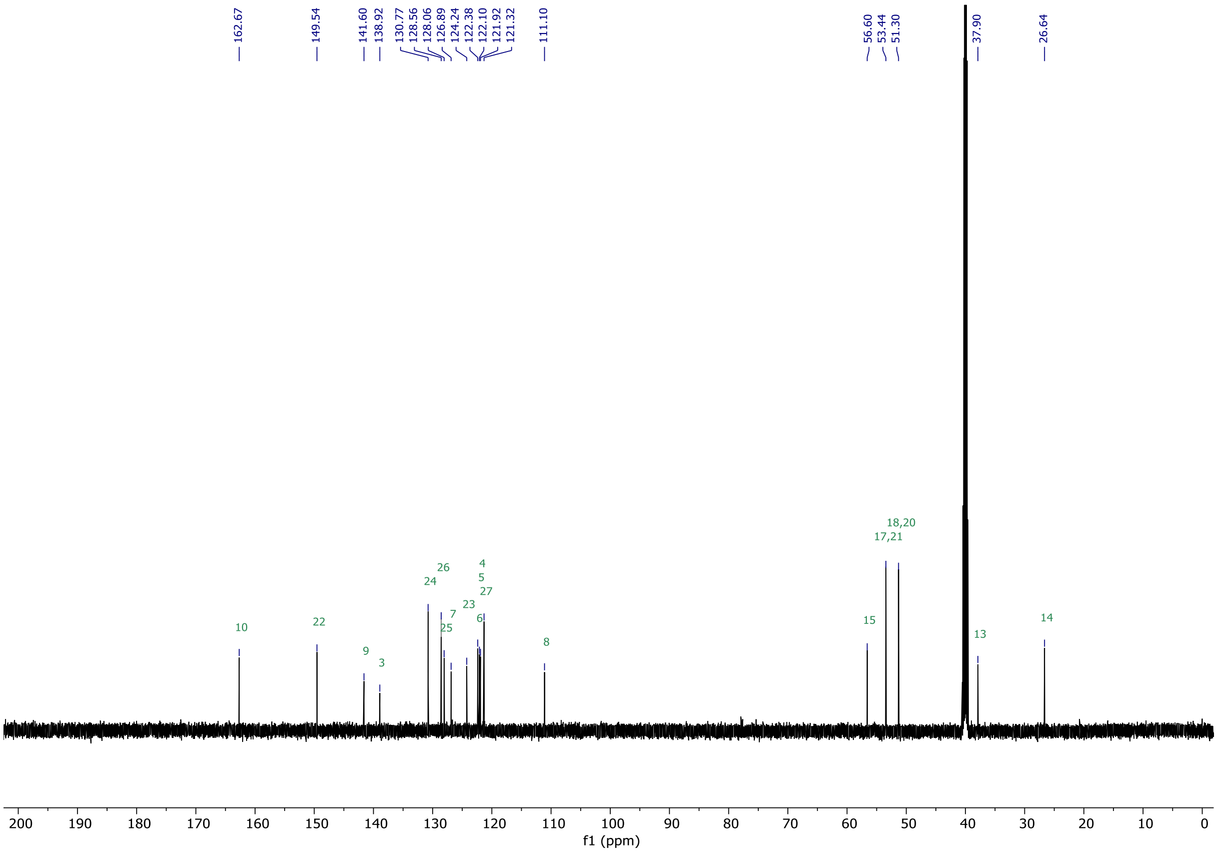

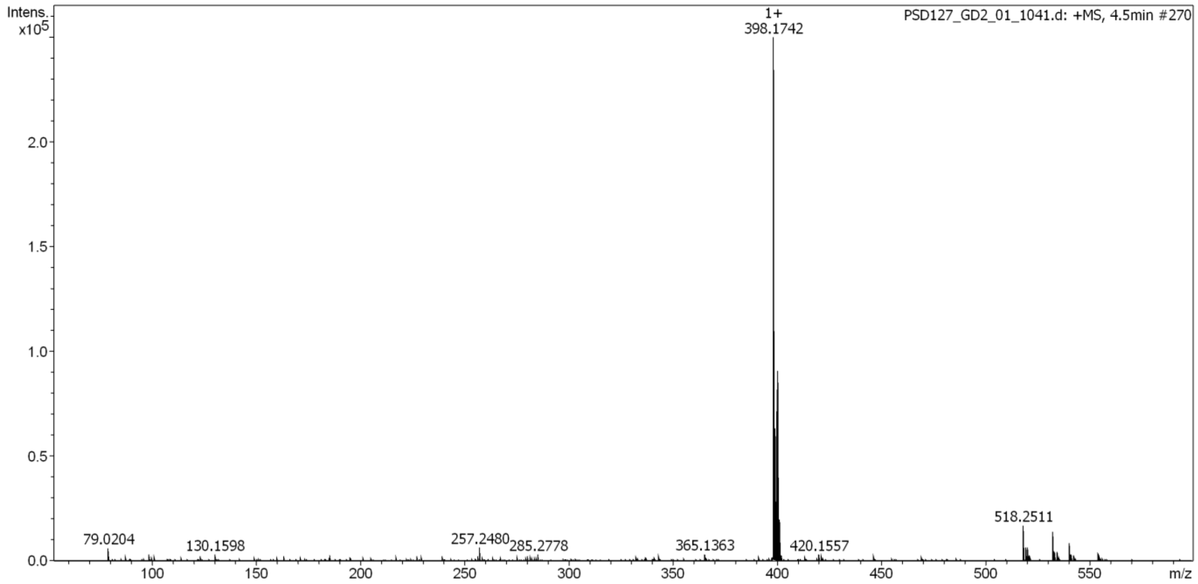
Intens.
x10^5
PSD127_GD2_01_1041.d: +MS, 4.5min #270
1+
398.1742
518.2511
79.0204
130.1598
257.2480
285.2778
365.1363
420.1557
2.0
1.5
1.0
0.5
0.0
100
150
200
250
300
350
400
450
500
550
m/z

N-(3-(4-(2-(methylthio)phenyl)piperazin-1-yl)propyl)-1*H*-indazole-3-carboxamide (14)

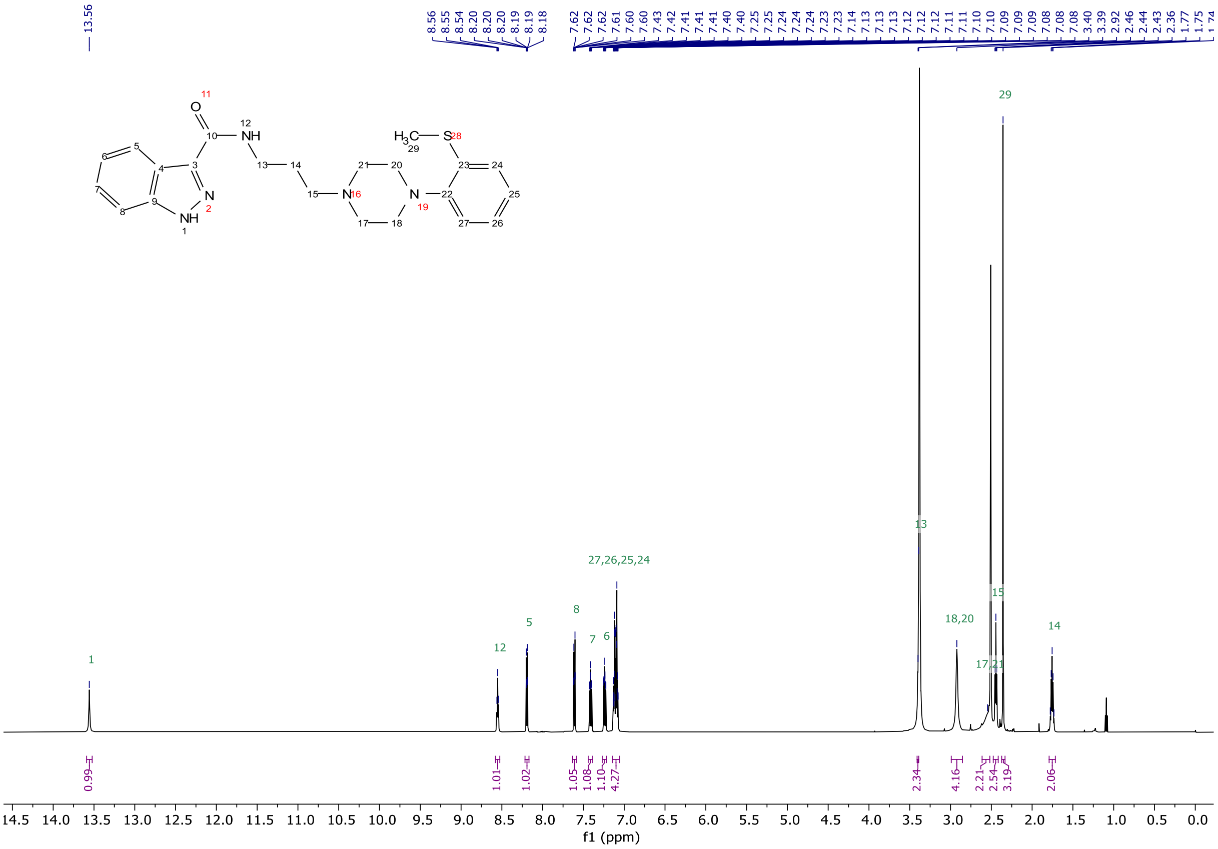

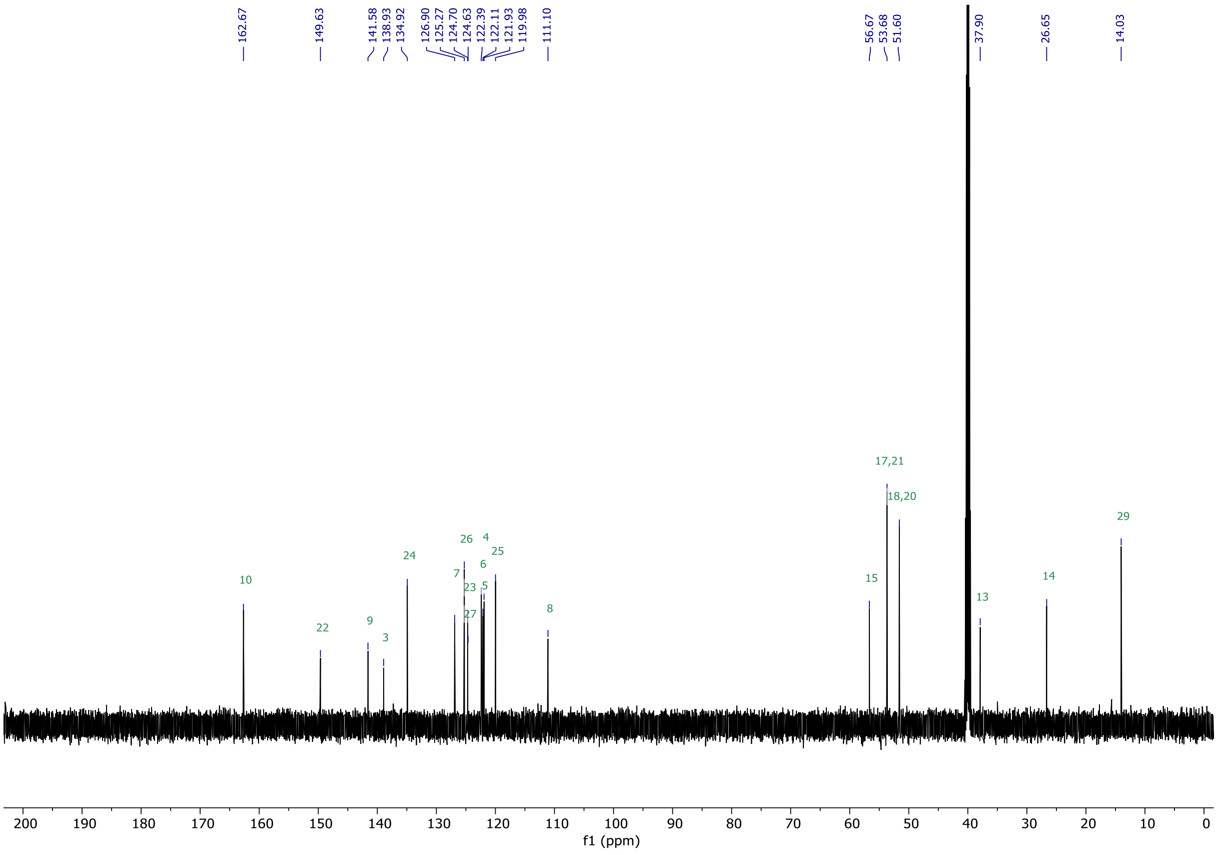

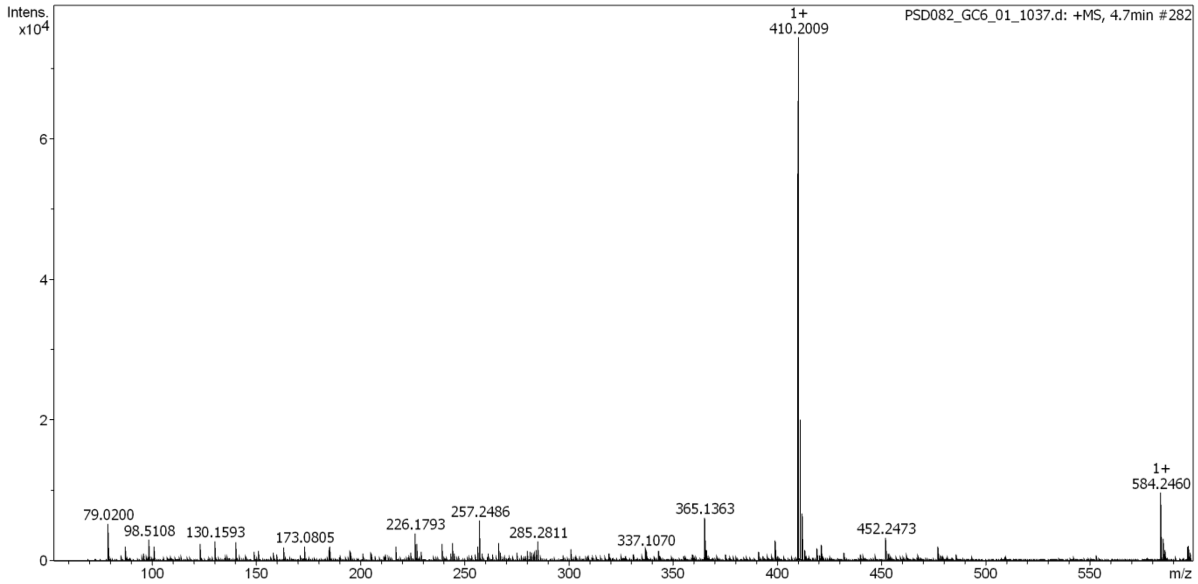

Intens.
x10^4
PSD082_GC6_01_1037.d: +MS, 4.7min #282
1+
410.2009
1+
584.2460
79.0200
98.5108
130.1593
173.0805
226.1793
257.2486
285.2811
337.1070
365.1363
452.2473
0
2
4
6
100
150
200
250
300
350
400
450
500
550
m/z

N-(3-(4-(2-ethoxyphenyl)piperazin-1-yl)propyl)-1*H*-indazole-3-carboxamide (15)

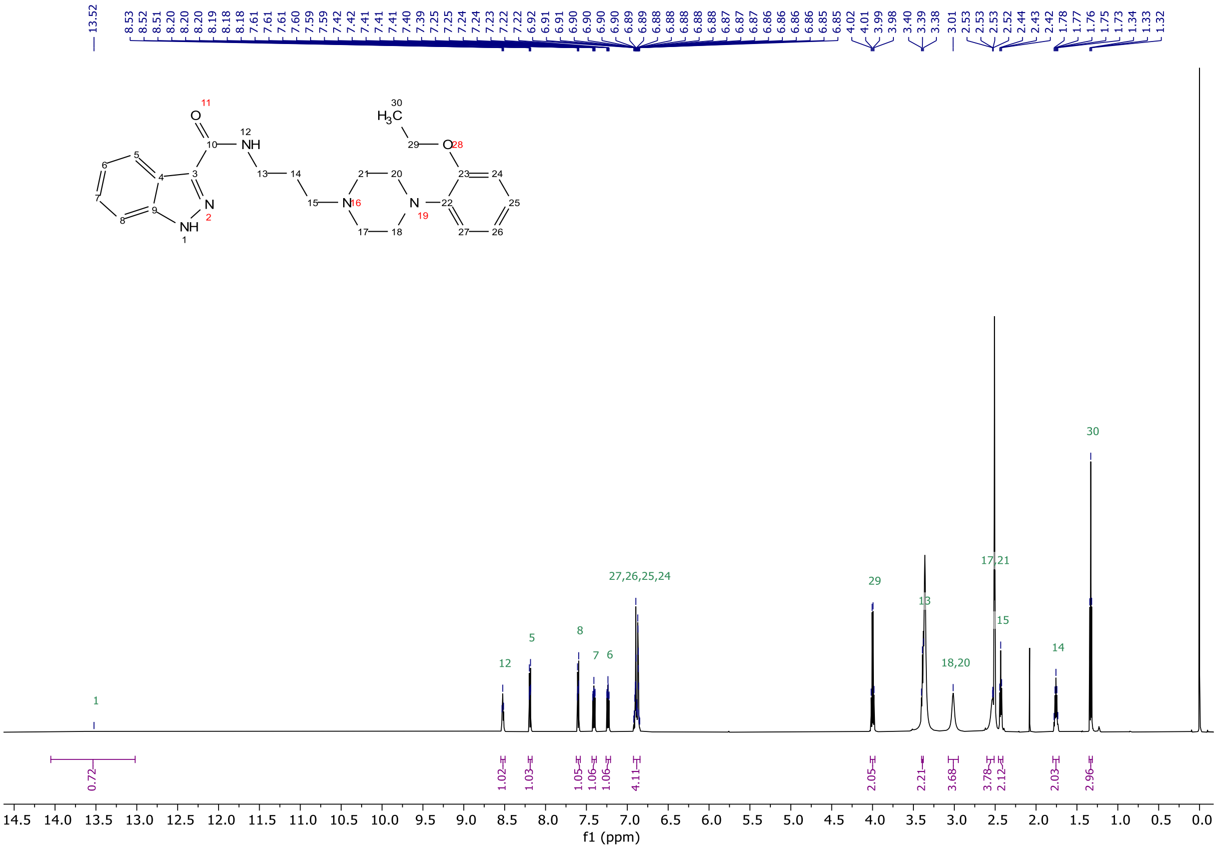

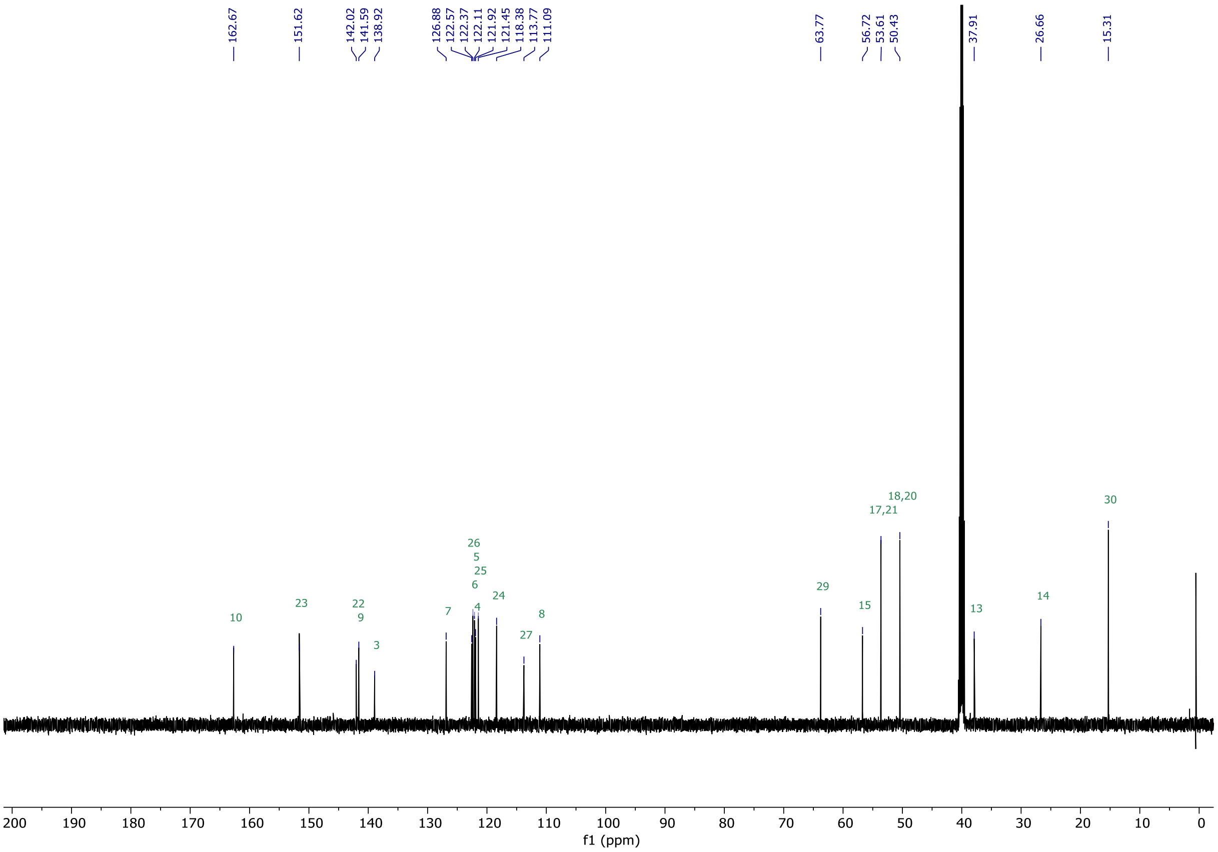

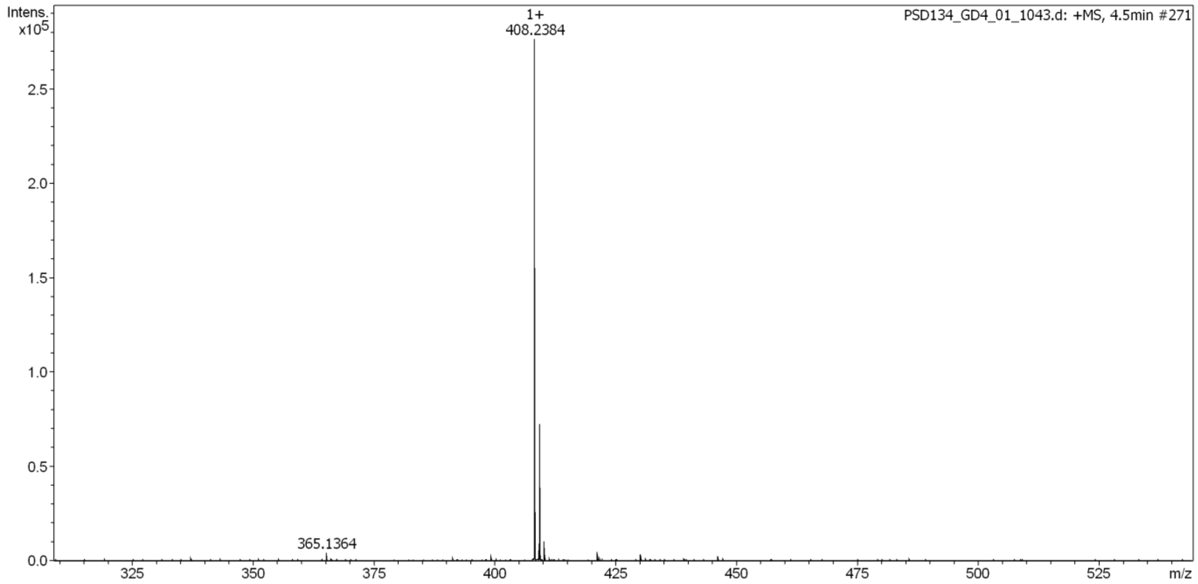

Intens.
x10^5
PSD134_GD4_01_1043.d: +MS, 4.5min #271
1+
408.2384
365.1364
2.5
2.0
1.5
1.0
0.5
0.0
325
350
375
400
425
450
475
500
525
m/z

***N*-(3-(4-cyclohexylpiperazin-1-yl)propyl)-1*H*-indazole-3-carboxamide (16)**

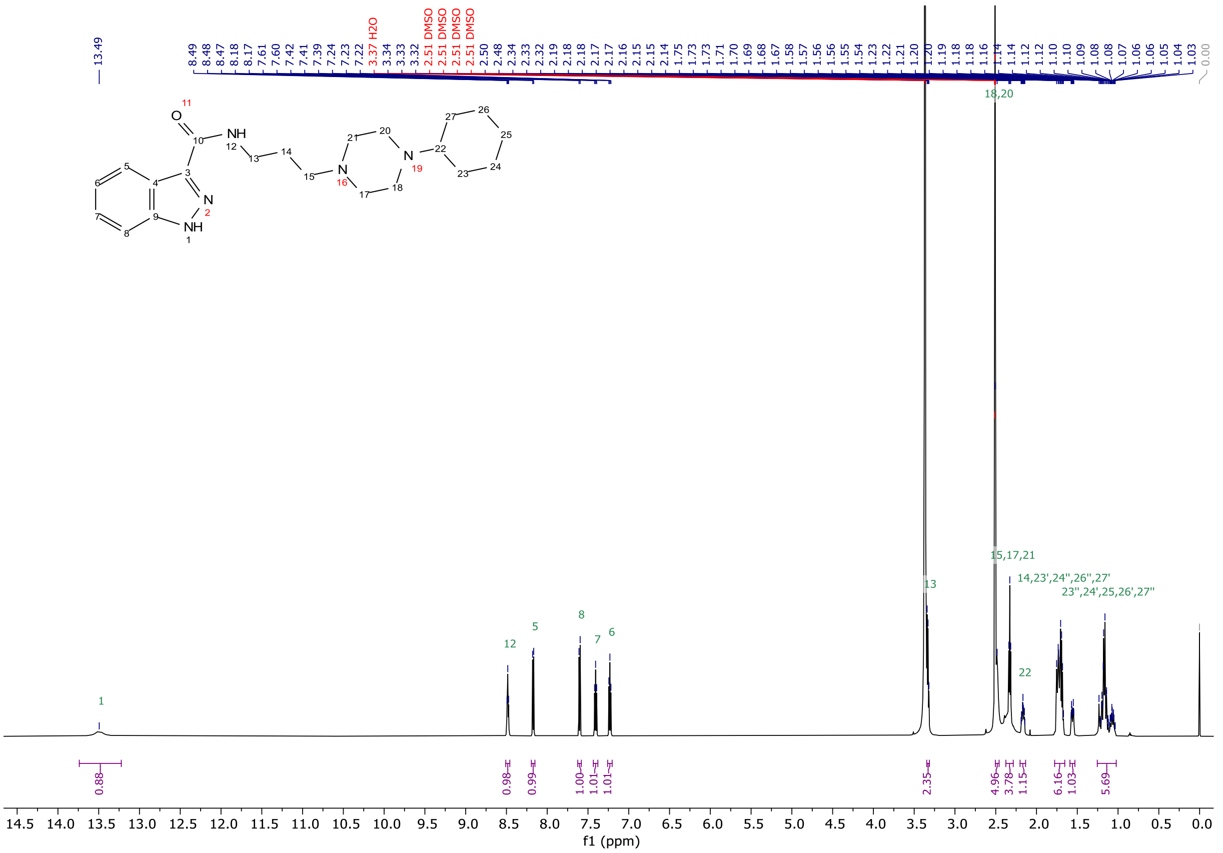

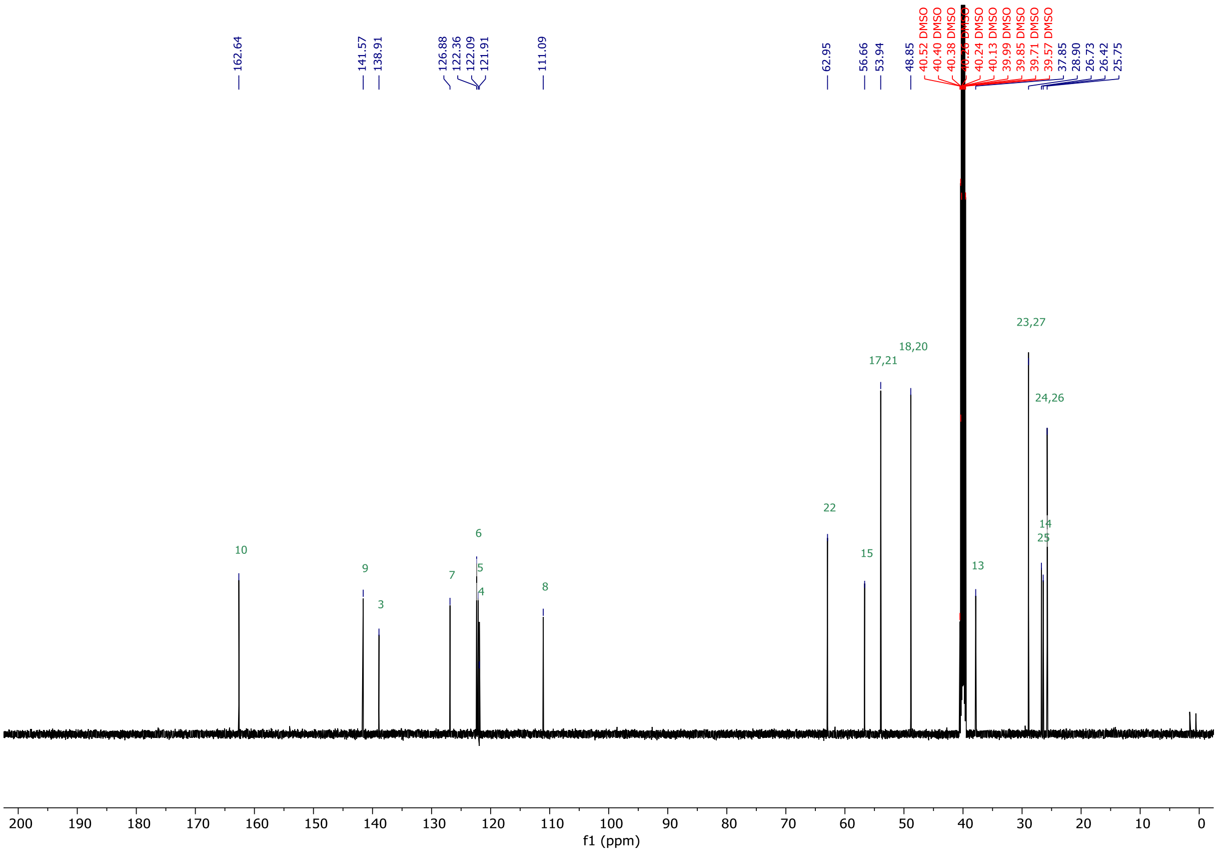

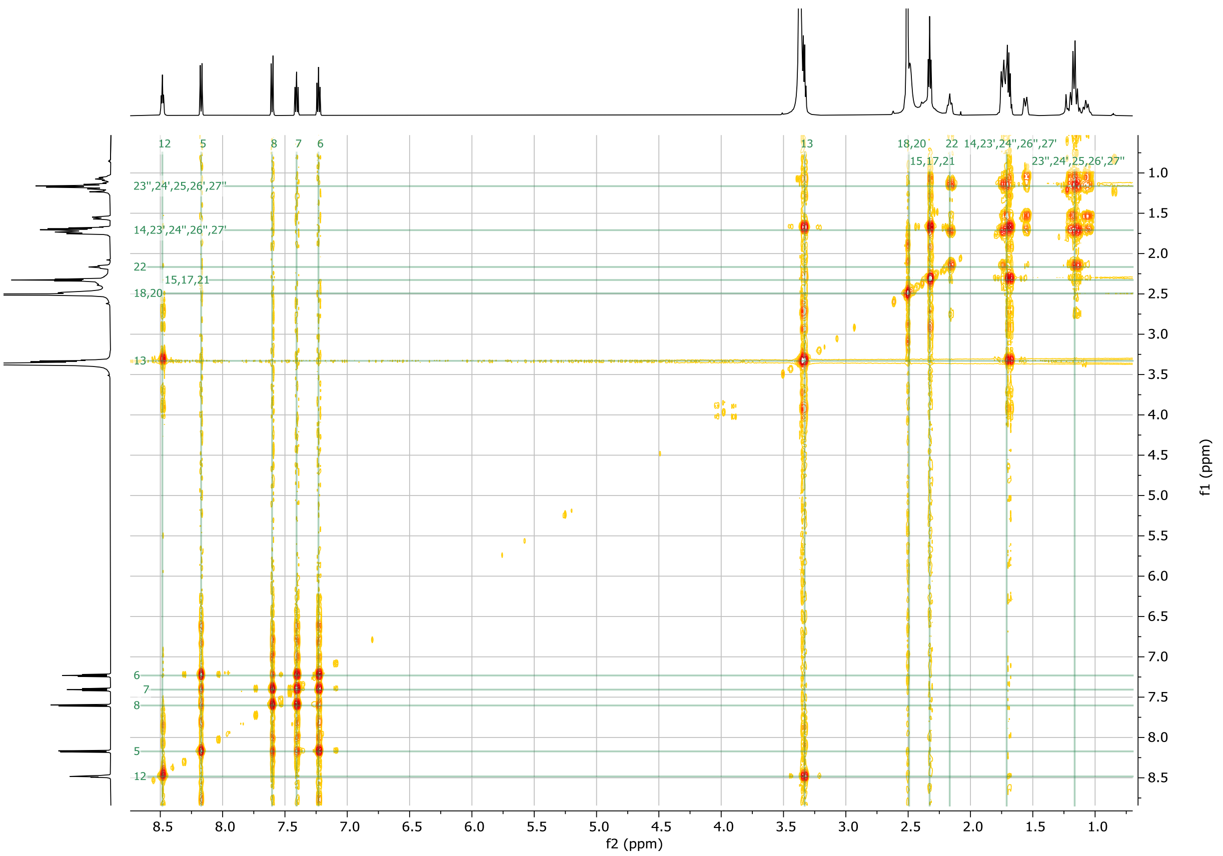
12
5
8
7
6
13
18,20
15,17,21
22
14,23',24",26",27'
23",24',25,26',27"
f2 (ppm)
f1 (ppm)

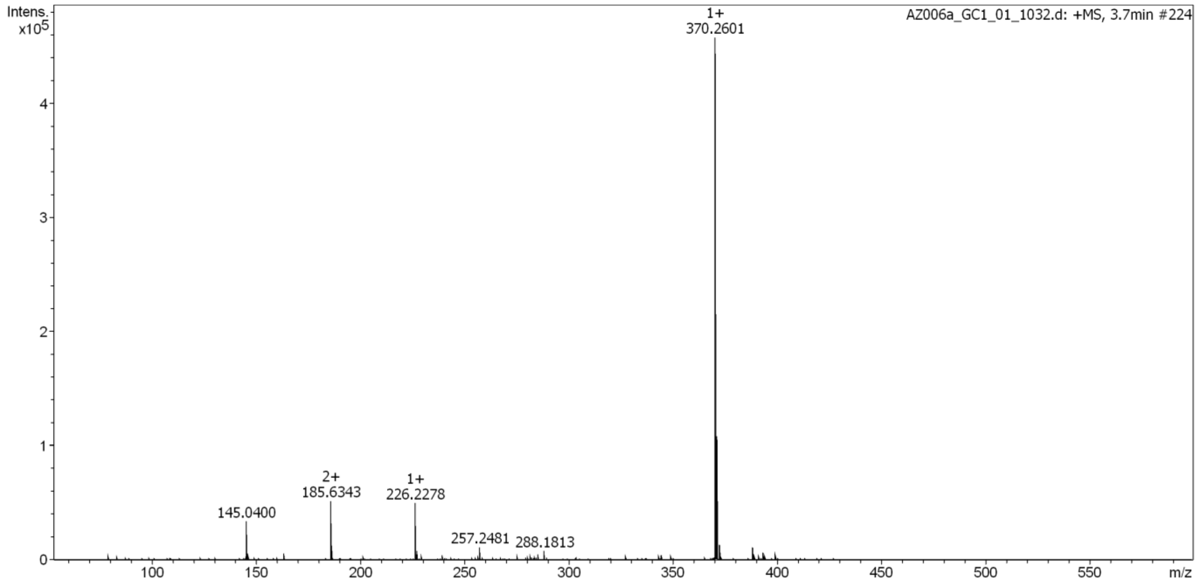
Intens.
x10^5
AZ006a_GC1_01_1032.d: +MS, 3.7min #224
1+
370.2601
145.0400
2+
185.6343
1+
226.2278
257.2481
288.1813
m/z
